# Polyoxygenated Terpenoids and Polyketides from the Roots of *Flueggea virosa* and Their Inhibitory Effect against SARS-CoV-2-Induced Inflammation

**DOI:** 10.3390/molecules27238548

**Published:** 2022-12-05

**Authors:** Ju-Chien Cheng, Yi-Ju Chen, Chi-Wen Chuang, Ya-Hsuan Chao, Hui-Chi Huang, Chia-Chi Lin, Chih-Hua Chao

**Affiliations:** 1 Department of Medical Laboratory Science and Biotechnology, China Medical University, Taichung 40402, Taiwan; 2 Core Facility Center, Office of Research and Development, Taipei Medical University, Taipei 110301, Taiwan; 3 School of Pharmacy, China Medical University, Taichung 406040, Taiwan; 4 Department of Chinese Pharmaceutical Sciences and Chinese Medicine Resources, China Medical University, Taichung 40402, Taiwan; 5 Chinese Medicine Research and Development Center, China Medical University Hospital, Taichung 404332, Taiwan

**Keywords:** SARS-CoV-2, anti-inflammatory, podovirosanes A–F, *Flueggea virosa*, DP4+

## Abstract

Six new polyoxygenated terpenoids, podovirosanes A–F (**1**–**6**), and two known polyketides (**7** and **8**) were isolated from the roots of *F. virosa*. Their structures, along with absolute configurations, were deduced using spectroscopic analysis as well as computational calculations, including TDDFT calculation of ECD spectra and GIAO NMR calculations combined with DP4+ probability analysis. Compounds **2**, **3**, **5**, and **8** were found to reduce the phosphorylation levels of NF-*κ*B p65 in SARS-CoV-2 pseudovirus-stimulated PMA-differentiated THP-1 cells.

## 1. Introduction

Coronavirus-induced disease COVID-19, a highly infectious disease caused by severe acute respiratory syndrome coronavirus 2 (SARS-CoV-2), has resulted in a major public health crisis since 2019 [1]. Although most of the infectious patients are mildly symptomatic or asymptomatic, the acute infectious case might result in an aggressive lung inflammation initiated by increased production of inflammatory cytokines from innate immune cells [2,3].

**Figure 1:**
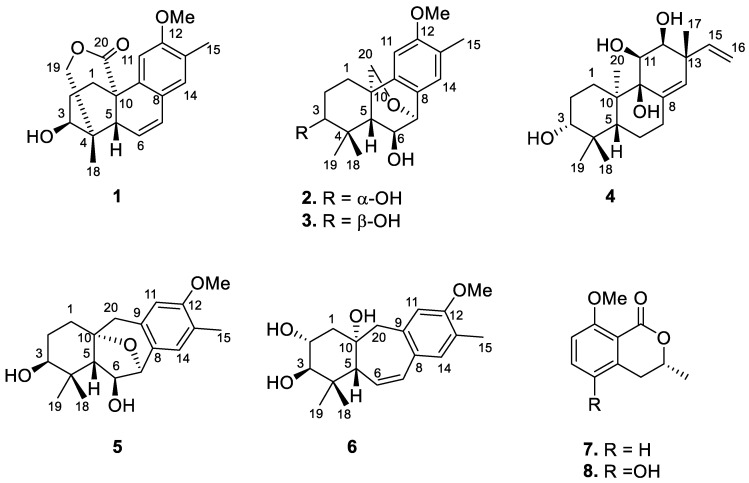
Structures of compounds **1**–**8**.

*Flueggea* plants, formerly classified asthe genus *Securinega*, were known to have 16 different species that were widely spread in Asia, Africa, and South America [4,5]. *Flueggea virosa*, belonging to the Euphorbiaceae family, were traditionally used as folk medicines in Asia and China and have been used for treating diverse ailments [4]. Although the anti-virus constituents have been reported from the title plants [6,7,8], the constituents for anti-inflammatory properties remain largely uninvestigated. Our group has developed an assay for screening the anti-inflammatory activity of compounds against SARS-CoV-2-induced inflammation, which was performed in this study. As part of our ongoing search for bioactive constituents from natural sources [9,10,11,12], six new *ent*-podocarpane-related terpenoids and two known polyketides (Figure 1) were isolated from the roots of *F. virosa*. Their structures were characterized using spectroscopic methods as well as quantum chemical calculations, including ECD and gauge-independent atomic orbital (GIAO) NMR calculations, of which the latter was combined with DP4+ analysis. Herein, the isolation, the structural elucidation, and the ability of isolates against SARS-CoV-2-induced inflammatory response were described.

## 2. Results

The molecular formula of podovirosane A (**1**) was established as C_19_H_22_O_4_ by analysis of its NMR data and HREIMS, equating to nine indices of hydrogen deficiency (Figures S1–S3). The IR spectrum revealed the presence of hydroxy (3456 cm^−1^), carbonyl group (1713 cm^−1^), and aromatic (1611 and 1567 cm^−1^) functionalities. The latter was evidenced by the UV maxima at 217 and 278 nm. The ^1^H NMR spectrum (Table 1) displayed signals of two phenyl singlets at δ_H_ 6.89 and 6.93, two methyl singlets at δ_H_ 2.18 and 1.19, a methoxy group at δ_H_ 3.88, an oxygenated methine at δ_H_ 3.81 (1H, br s), oxygenated methylene at δ_H_ 4.04 (1H, dd, *J* = 12.2, 1.7 Hz) and 4.37 (1H, d, *J* = 12.2 Hz) as well as two mutually coupled, olenfinic protons at δ_H_ 5.90 (1H, br d, *J* = 9.8 Hz) and 6.60 (1H, dd, *J* = 9.8, 2.9 Hz). The ^13^C NMR spectrum (Table 2) showed two oxygenated sp^3^ carbons [δ_C_ 75.4 (CH_2_) and 74.5 (CH)], a methoxy carbon [δ_C_ 55.4 (CH_3_)], eight sp^2^ carbons [δ_C_ 122.8 (CH), 131.2 (CH), 125.2 (qC), 134.8 (qC), 108.1 (CH), 157.4 (qC), 125.9 (qC), and 129.0 (CH)], and an ester carbonyl [δ_C_ 174.5 (qC)]. The above data represent five indices of hydrogen deficiency, while the other four suggested the tetracyclic nature of **1**.

**Figure 2:**
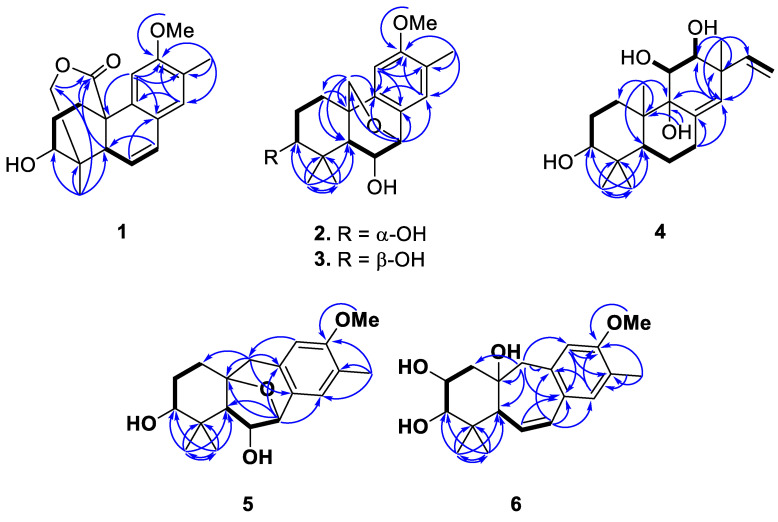
Selected ^1^H-^1^H COSY (▬) and HMBC (→) correlations of **1**–**6**.

**Figure 3:**
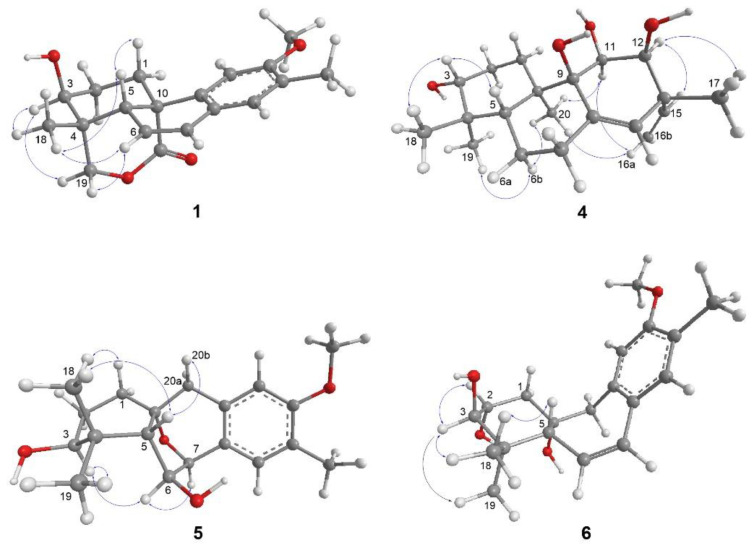
Selected NOE correlations of **1** and **4**–**6** (a: deshielded proton; b: shielded proton).

**Figure 4:**
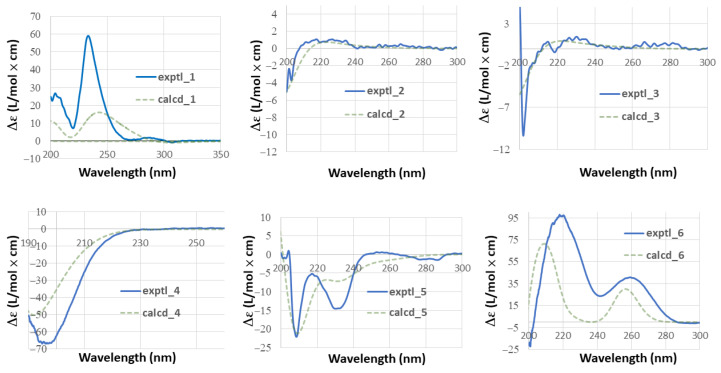
Experimental and calculated ECD spectra of **1**–**6**.

A 2-methyl-4,5-disubstituted anisole fragment was evidenced by HMBC correlations, which were observed from H_3_-15 to C-12, C-13, and C-14, from methoxy protons to C-12, from H-14 to C-9 and C-12, and from H-11 to C-8, C-12, and C-13 (Figure 2 and Figure S6). In combination with the two spin systems from H_2_-1 to H-3 and H-5 to H-7 established by analysis of COSY correlations, the HMBC correlations from H-14 to C-7, from H-11 to C-10, from H_3_-18 to C-3, C-4, C-5, and C-19, and from H_2_-1 to C-20, C-5, and C-10 allowed the establishment of a 13-methylpodocarpane framework in **1** (Figure 2 and Figure S7). Moreover, the HMBC correlations from oxymethylene protons (H_2_-19) to the carbonyl carbon (C-20) allowed the connectivity of the ester linkage between C-19 and C-20 (Figure 2). The relative configuration of the ester bridge was assigned as α-oriented being opposite to H-5 according to the NOE correlation of H-5/H_3_-18 (Figure 3 and Figure S8). The small ^3^*J*_H,H_ value of H-3 and its NOE correlations with both H_3_-18 and one proton of H_2_-19 (δ_H_ 4.04), of which the latter displayed a ^4^*J* W-coupling (1.7 Hz) with H-5, suggested that H-3 is located on equatorial position and assigned as α-proton. The relative configuration of C-3 was further confirmed by GIAO NMR calculations of the two possible candidates with 3β-OH and 3α-OH substituents (Figure S48), followed by DP4+ probability analysis [13]. The result revealed that the all-data probability of the 3β-OH candidate was found to be 100% (Table 3 and Table S1) and thus suggested it to be the correct structure. Finally, a comparison of experimental and calculated ECD data established the absolute configuration of **1** as 3*S*, 4*R*, 5*R*, 10*S* (Figure 4).

Podovirosane B (**2**) has a molecular of C_19_H_26_O_4_ as determined from the (–)-HRAPCIMS and NMR data (Figures S9–S12). The ^1^H and ^13^C NMR data of **2** (Table 1 and Table 2) showed remarkable similarity to a known analog, 7α,20-epoxy-3α-hydroxy-12-methoxy-13-methyl-*ent*-podocarp-8,11,13-triene (**2a**), isolated from the same plant [6], and the obvious differences between these two compounds were the chemical shifts of C-6, of which the aliphatic methylene (δ_C_ 29.9; δ_H_ 2.13, 1.58) in **2a** [6] was replaced by a hydroxy-containing methine (δ_C_ 68.9; δ_H_ 4.21) in **2**. Detailed analysis of HMBC, HSQC, and COSY spectra (Figures S13–S15) gave evidence to support the structure of **2,** as shown in Figure 2. The NOE correlations of H-5/H-3 and H-5/H-1a suggested that ring A adopted a chair conformation and implied that these protons were cofacial (β-orientation) (Figure S16). A four-bond *W*-coupling observed between H-20b (δ_H_ 2.72) and H-5 (δ_H_ 0.91) suggested the α orientation of this ether functionality between C-7 and C-20. Although H-5 was found to have an NOE correlation with H-6, a comparison of 6α-OH and 6β-OH models disclosed that in both candidates, the proton distances between H-5 and H-6 were close enough to give NOE enhancements (Figure S49). Thus, the NOE correlations were unable to correlate the relative configuration of C-6. Using the same GIAO NMR calculation method as described above and applying the DP4+ probability on possible candidates of **2** (Figure S48), the (6β-OH)-**2** was suggested with a high probability of 100% (Table 3 and Table S4).

Podovirosane C (**3**) was found to have a negative [M − H]^−^ ion peak in HRESIMS, which, in combination with the NMR data (Table 1 and Table 2), indicated the same molecular formula as that of **2**. A comparison of the NMR data of **3** with those of **2** disclosed that they are epimers, with the difference being the NMR chemical shifts of C-3. Similarly, the chair-form ring A was evidenced by the NOE correlations of H-5/H-1a and H_3_-19/H-2a. Moreover, the proton signal of H-3 displayed as a broad singlet revealed the equatorial nature of this proton, suggesting the 3-OH to be β-oriented. The absolute configurations of **2** (3*R*,5*S*,6*S*,7*S*,10*S*) and **3** (3*S*,5*S*,6*S*,7*S*,10*S*) were also determined by the TDDFT-ECD calculations (Figure 4).

Podovirosane D (**4**) was determined to have a molecular formula of C_20_H_32_O_4_ based on the NMR data and HRESIMS at *m*/*z* 335.2221 [M − H]^−^ (Table 1 and Table S12). The ^1^H NMR data (for ^1^H NMR data, see Table S12) displayed signals characteristic to four methyls [δ_H_ 1.36 (3H, s), 1.29 (3H, s), 1.16 (3H, s), 1.08 (3H, s)], a vinyl group [δ_H_ 5.11 (1H, d, *J* = 10.4 Hz), 5.21 (1H, d, *J* = 17.4 Hz), 5.91 (1H, dd, *J* = 17.4. 10.4 Hz)], a tri-substituted double bond [δ_H_ 5.35 (1H, s)], three oxygenated methines [δ_H_ 3.62 (1H, m), 4.05 (1H, br s), 4.31 (1H, br d, *J* = 8.0 Hz)], and four exchangeable protons of OH groups [δ_H_ 4.79 (1H, br s), 5.75 (1H, br d, *J* = 4.6 Hz), 5.15 (overlapped), 6.87 (1H, br s)]. The above data disclosed that **4** was structurally related to a known pimarane, *ent*-3β,12α-dihydroxypimara-8(14),15-diene [6]. The above data, in combination with the ^13^C NMR data, suggested that it has four hydroxy groups, including three secondary and one tertiary. The OH group attached at C-3 was assigned according to the COSY correlations from H_2_-1 to H_2_-2 and from H_2_-1 to H-3, as well as the HMBC correlations from both H_3_-18 and H_3_-19 to C-3, C-4, and C-5 (Figure 2 and Figure S29). The OH group attached at C-9 was assigned on the basis of the HMBC correlations from H_3_-20 to C-1, C-5, C-9, and C-10. The 11,12-dihydroxy substituents were elucidated from the HMBC correlations of H_3_-17 with C-12, C-13, C-14, and C-15, in conjunction with a COSY correlation between H-11 and H-12. The NOE correlations of H-12/H-15, H-12/H_3_-17, H-11/H-16a, and H_3_-20/H-11 led to the assignment of H-11, H-12, H_3_-20, and the vinyl group as α-orientations (Figure 3 and Figure S31). The assignment of α-orientation of H_3_-19 is based on the NOE correlations of H_3_-19/H-6b and H_3_-20/H-6b. In contrast, the NOE correlations of H-3/H-5 and H-3/H_3_-18 indicated the β-orientations of H-3 and H-5. Although no diagnostic NOE correlation was found for the OH proton at C-9, the correlation of H_3_-20/H-11 allowed the establishment of the 9-OH group to be β-oriented. Moreover, the calculated ECD spectrum of **4** showed good agreement with the experimental data (Figure 4), which established its absolute configuration as 3*R*, 5*R*, 9*S*, 10*R*, 11*S*, 12*S*, 13*R*.

The (–)-HRAPCIMS of podovirosane E (**5**) displayed a deprotonated molecular ion [M − H]^−^, consistent with a molecular formula of C_19_H_26_O_4_ (Figure S32). The ^1^H and ^13^C NMR data (Table 1 and Table 2) were closely related to those of 9(10→20)-*abeo*-*ent*-podocarpanes [7], and differences should be substitution patterns in ring B. The HMBC correlations from H-7 to C-9, C-14, and C-10 as well as correlations from H_2_-20 to C-8, C-9, and C-10, indicated that an ether linkage should be assigned between C-7 and C-10 (Figure 2 and Figure S37). The COSY correlations of H-6 with H-6 and H-7 and of H_2_-2 with H_2_-1 and H-3 suggested the C-5/C-6/C-7 and the C-1/C-2/C-3 spin systems (Figure S38). Further HMBC correlations from H_3_-18 (or H_3_-19) to C-3, C-4, and C-5 connected the two spin systems and thus established the planar structure of **5**. The NOE correlation of H_3_-18/H-1 suggested ring A should present as boat conformation [14]. The NOE correlation of H-6/H-3 arbitrarily assigned these two protons as α-oriented, whereas the correlations of H-5/H-20b and H-5/H_3_-18 suggested the β-orientation of H-5 and the α-oriented C-7–C-10 ether linkage (Figure 3 and Figure S39). The absolute configuration of **5**, as shown in Figure 1, was further corroborated by comparing the calculated and experimental ECD data (Figure 4).

The (+)-HRESIMS of podovirosane F (**6**) displayed a sodiated molecular ion [M + Na]^+^, revealing a molecular formula of C_20_H_30_O_4_ (Figure S40). The ^1^H and ^13^C NMR data (Table 1 and Table 2) of **6** suggested it to be an analog of the known compound, 3*β*,10α-dihydroxy-12-methoxy-13-methyl-9(10 → 20)-*abeo*-*ent*-podocarpa-6,8,11,13-tetraene (**6a**), which is reported from the leaves of the same plant [15]. Analysis of ^13^C NMR data of **6** disclosed that it has an additional hydroxyl group at ring A as compared to **6a**. A 2,3-dihydroxy substituent could be readily assigned by the COSY correlations between H-2 and H-3 and between H_2_-1 and H-2 (Figure 2). Further HMBC and COSY correlations (Figure 2, Figures S45 and S46) confirmed such a planar structure of **6**. The NOE correlations of H-2/H-3, H-3/H_3_-19, H-3/H_3_-18, and H-5/H_3_-18 implied that H-5 and H-3 should be located on axial and equatorial positions, respectively; however, the orientation of H-2 remains unclear as either equatorial or axial H-2 is likely to show NOE correlation with equatorial H-3 (Figure 3 and Figure S47). In turn, analysis of the coupling constant between H-2 and H_2_-1 (Table 1) revealed that H-2 should be located in an equatorial position, suggesting the α-orientation of the attached OH group at C-2. On the other hand, the relative configuration of C-10 was also unable to be determined due to the lack of crucial NOE correlations. In order to clarify the configuration of C-10 and also to confirm the relative configuration of C-2, the GIAO NMR calculation and DP4+ probability analysis were performed for the four possible candidates, (2α,10α-OH)-**6**, (2β,10α-OH)-**6**, (2α,10β-OH)-**6**, and (2β,10β-OH)-**6** (Figure S48). The result suggested that (2α,10α-OH)-**6** with 2α,10α-dihydroxy substituents has a high probability of 100% (Table 3 and Table S7). With a similar procedure of ECD analysis upon **5**, the absolute configuration of **6** was determined as 2*R*,3*R*,5*R*,10*S* (Figure 4).

The known compounds were identified to be (*R*)-8-methoxymellein (**7**) [16] and (3*R*)-5-hydroxy-8-*O*-methylmellein (**8**) [17,18] by comparing their spectroscopic data with those reported in the literature.

**Figure 5:**
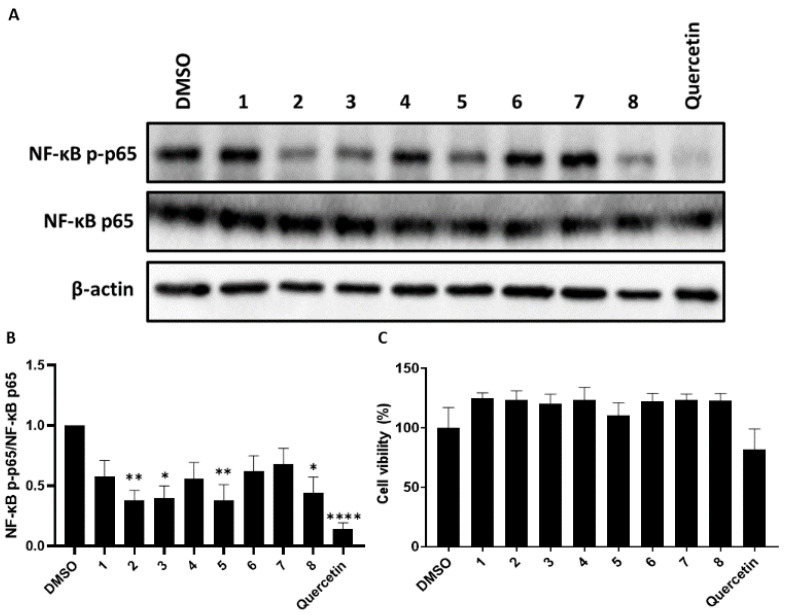
The SARS-CoV-2 pseudovirus-stimulated THP-1 macrophages were treated with individual compounds (25 μM) for 18 h, and each of the protein lysates were collected for Western blotting. DMSO was used as a solvent control. (**A**) Phosphorylated NF-*κ*B p65 and NF-*κ*B p65 were determined by indicated antibody. GAPDH was used as a loading control. (**B**) Densitometric analysis of Western blots was performed by Image J. Three individual experiments were performed. The ratio of phosphorylated NF-*κ*B (p-p65) versus the expression level of NF-*κ*B (p65) was presented. (**C**) The THP-1 macrophages were treated with compounds (25 μM) for 48 h. Cell viability was determined by MTS assay. The data show the means ± SD of three independent experiments and were analyzed by Prism (www.graphpad.com, accessed on 2 November 2022). Statistic comparison among the groups was performed by one-way ANOVA (* *p* < 0.05, ** *p* < 0.01, **** *p* < 0.0001).

Since SARS-CoV-2 Spike protein can stimulate inflammation [19,20,21,22], we established a platform to screen the activity of SARS-CoV-2 induced-inflammation for the isolated compounds. A preliminary evaluation of the cytotoxicity of the isolates toward the human monocytic cell line (THP-1) was performed using an MTS assay. The results revealed that compounds **1**–**8** were not found to exhibit toxicity toward THP-1 cells at a concentration of 25 μM (Figure 5A,C). The anti-inflammatory activity was performed by evaluating the expression of Phospho-NF-kappaB p65 (p-NF-*κ*B p65) in SARS-CoV-2 pseudovirus-stimulated phorbol 12-myristate 13-acetate (PMA)-differentiated THP-1 cells. The result of western blot analysis revealed that compounds **2**, **3**, **5**, and **8**, at a concentration of 25 μM, reduced the phosphorylated status of NF-κB in SARS-CoV-2 pseudovirus-stimulated THP-1 macrophages (Figure 5A,B).

## 3. Materials and Methods

### 3.1. General Experimental Procedures

Column chromatography was performed in a glass column using Silica gel 60 (40–63 µm, Merck, Darmstadt, Germany) and SiliaBond C18 silica gel (40–63 µm, 60 Å, 17% carbon loading, Silicycle, Quebec, Canada), and the fractions were pooled according to the TLC analysis using precoated silica gel plates (Kieselgel 60 F_254_, 0.25 mm, Merck, Darmstadt, Germany) and silica gel RP-18 plates (Kieselgel 60 F_254_S, Merck, Darmstadt, Germany). UV and IR spectra were recorded on a PerkinElmer Lambda-265 UV-Vis and a PerkinElmer Spectrum Two FT-IR spectrometers (PerkinElmer Inc., Waltham, MA, USA), respectively. Optical rotations were determined at 25 °C with a JASCO P2000 digital polarimeter (JASCO Co., Tokyo, Japan). The NMR spectra, including the 2D NMR experiments, were performed on Bruker Avance-400 and -500 spectrometers (Bruker BioSpin, Rheinstetten, Germany) with chemical shifts reported in ppm referenced to *δ*_C_ 77.0/*δ*_H_ 7.26 ppm (CDCl_3_) and *δ*_C_ 135.5/*δ*_H_ 7.58 ppm (pyridine-*d*_5_).

### 3.2. Plant Material

The roots of *F. virosa* were collected from Pingtung County, Taiwan, in September 2011 and authenticated by Prof. C. S. Kuoh of the Department of Life Sciences, National Cheng Kung University, Tainan, Taiwan. A voucher herbarium specimen, FV-Chao001, has been deposited in the School of Pharmacy, China Medical University, Taichung, Taiwan.

### 3.3. Extraction and Isolation

The dried roots of *F. virosa* (13.0 kg) were minced and soaked in MeOH (4 × 20 L) at room temperature. The combined extract was concentrated to give an oily brown residue and subsequently partitioned between CHCl_3_ and H_2_O. The CHCl_3_ layer was washed with 3% tartaric acid to afford an alkaloid-free extract (93 g) which was fractionated using silica gel column chromatography (CC) with a solvent gradient composed of hexane–EtOAc (100:0 to 0:100) and EtOAc–MeOH (100:0 to 0:100) to yield 26 fractions. Fraction 17 was fractionated by a silica gel column and eluted with a gradient eluent mixture (hexane/EtOAc = 75:25 to 70:30) to give 15 subfractions (fr.17A–17O). Fr.17G was purified by a short column of silica gel, using hexane/EtOAc = 3:2 as eluent, to obtain compound **7** (2.4 mg). Fr.17J was divided into 15 subfractions (fr.17J1–17J15) based on a gradient elution of RP-18 CC (eluent: MeOH/H_2_O = 63:47 to 80:20). Compounds **3** (0.8 mg) and **5** (4.4 mg) were obtained from fr.17J3 using semipreparative HPLC (Inertsil ODS-3, 5 μm, 250 × 10 mm; MeOH/H_2_O = 50:50). Compound **6** (4.4 mg) was also purified by HPLC using ODS-3 column and a mixture of MeOH/H_2_O = 60:40 as eluent. Fr.17K was subjected to RP-18 CC using stepwise gradient elution (eluent: MeOH/H_2_O = 40:60 to 74:26) to afford nine subfractions (fr.17K1–17K9). Among them, fr.17K1 was further fractionated using the same gradient elution as that of fr.17K and pooled according to TLC to yield 13 subfractions (fr.17K1A–17K1M). Compound **8** (1.2 mg) was yielded from fr. 17K1B by HPLC (ODS-3, MeOH/H_2_O = 35:65). Fr. 17K1L was purified by silica gel CC, eluted with hexane/EtOAc = 1:1, to afford compound **4** (3.2 mg). Compounds **2** (1.1 mg) and **1** (1.2 mg) were obtained from fr.17K1D and fr.17K1G, respectively, using HPLC (ODS-3, MeOH/H_2_O = 50:50 for **2**; MeOH/H_2_O = 60:40 for **1**).

Podovirosane A (**1**): colorless oil; ${\left[\alpha \right]}_{D}^{25}$ +148.3 (*c* 0.10, MeOH); UV (MeOH) *λ*_max_ (log ε): 205 (4.35), 217 (4.23), 278 (3.85) nm; (IR (KBr) *v*_max_ 3456, 2936, 2875 1713, 1611, 1567, 1508,1467, 1281, 1216, 1173, 1149, 1115 cm^−1^; ^1^H and ^13^C NMR data, see Table 1 and Table 2; EIMS *m*/*z* 314 [M]^+^; HREIMS *m*/*z* 314.1524 [M]^+^ (calcd for C_19_H_22_O_4_, 314.1518).

Podovirosane B (**2**): white powder; ${\left[\alpha \right]}_{D}^{25}$ +15.1 (*c* 0.08, MeOH); UV (MeOH) *λ*_max_ (log ε): 203 (4.17), 225 (3.77), 276 (3.35) nm; IR (KBr) *v*_max_ 3415, 2926, 2859, 1585, 1497, 1287, 1091, 1066 cm^−1^; ^1^H and ^13^C NMR data, see Table 1 and Table 2; (–)-ESIMS *m*/*z* 317 [M − H]^−^; (–)-HRESIMS *m*/*z* 317.1753 [M − H]^−^ (calcd for C_19_H_25_O_4_, 317.1758).

Podovirosane C (**3**): colorless oil; ${\left[\alpha \right]}_{D}^{25}$ +35.6 (c 0.11, MeOH); UV (MeOH) *λ*_max_ (log ε): 205 (4.30), 229 (3.96), 276 (3.56) nm; IR (KBr) *v*_max_ 3402, 2953, 2870, 1585, 1498, 1465, 1365, 1287, 1148 cm^−1^; ^1^H and ^13^C NMR data, see Table 1 and Table 2; (–)-APCIMS *m*/*z* 317 [M − H]^−^; (–)-HRAPCIMS *m*/*z* 317.1748 [M − H]^−^ (calcd for C_19_H_25_O_4_, 317.1758).

Podovirosane D (**4**): white powder; ${\left[\alpha \right]}_{D}^{25}$ −70.3 (*c* 0.32, MeOH); IR (CaF2) *v*_max_ 3390, 2940, 2872, 1635, 1634, 1454, 1407, 1259 cm^−1^; ^1^H NMR data (pyridine-*d*_5_, 400 MHz): δ_H_ 6.87 (1H, br s, 12-O*H*), 5.91 (1H, dd, *J* = 17.4, 10.4 Hz, H-15), 5.75 (1H, br d, *J* = 4.6 Hz), 5.35 (1H, s, H-14), 5.21 (1H, d, *J* = 17.4 Hz), 5.15 (1H, overlapped, 11-O*H*), 5.11 (1H, d, *J* = 10.4 Hz), 4.79 (1H, br s, 9-O*H*),4.31 (1H, br d, *J* = 8.0 Hz, H-11), 4.05 (1H, br s, H-12), 3.62 (1H, m, H-3), 2.89 (1H, m, H-7a), 2.39 (1H, m, H-1a), 2.37 (2H, m, H-5 and H-7b), 2.25 (1H, m, H-1b), 1.97 (2H, m, H_2_-2), 1.75 (1H, m, H-6a), 1.56 (1H, m, H-6b), 1.36 (3H, s, H_3_-17), 1.29 (3H, s, H_3_-18), 1.16 (3H, s, H_3_-19). 1.08 (3H, s, H_3_-20); ^13^C NMR, see Table 2; (–)-ESIMS *m*/*z* 335 [M − H]^−^; (–)-HRESIMS *m*/*z* 335.2221 [M − H]^−^ (calcd for C_20_H_31_O_4_, 317.2228).

Podovirosane E (**5**): white powder; ${\left[\alpha \right]}_{D}^{25}$ −9.1 (*c* 0.39, MeOH); UV (MeOH) *λ*_max_ (log ε): 203 (4.26), 225 (3.82), 280 (3.33) nm; IR (KBr) *v*_max_ 3427, 2947, 1618, 1585, 1505, 1464, 1367, 1257, 1217 cm^−1^; ^1^H and ^13^C NMR data, see Table 1 and Table 2; (–)-APCIMS *m*/*z* 317 [M − H]^−^; (–)-HRAPCIMS *m*/*z* 317.1752 [M − H]^−^ (calcd for C_19_H_25_O_4_, 317.1758).

Podovirosane F (**6**): colorless oil; ${\left[\alpha \right]}_{D}^{25}$ +188.6 (*c* 0.44, MeOH); UV (MeOH) *λ*_max_ (log ε): 216 (4.45), 260 (3.96) nm; IR (KBr) *v*_max_ 3400, 3018, 2926, 1609, 1566, 1504, 1464, 1391, 1315, 1256, 1217 cm^−1^; ^1^H and ^13^C NMR data, see Table 1 and Table 2; (+)-ESIMS *m*/*z* 341 [M + Na]^+^; (+)-HRESIMS *m*/*z* 341.1720 [M + Na]^+^ (calcd for C_20_H_30_O_4_Na, 341.1723).

### 3.4. ECD Calculations

Conformational searches were performed using GMMX add-on in GaussView with MMFF94 force field. The resulting conformers within a 10 kcal/mol window were subjected to geometry optimizations and frequency checks for local minimum using Gaussian 16 software [23] at B3LYP/6-31G(d) level of theory. After removal of duplicated conformers, the ECD spectra were simulated from the TDDFT calculation at SMD/CAM-B3LYP/6-311+G(d,p) level of theory in MeOH (compounds **1**–**3**, and **5**) and ACN (compound **4**), while compound **6** was performed at SMD/M062x/6-311+G(d,p) level of theory in MeOH. The spectra were averaged according to the Boltzmann distribution using energies calculated at the same level of theory.

### 3.5. NMR Calculations and DP4+ Analysis

The conformers for possible candidates of **1**, **2**, and **6** were obtained from calculations at B3LYP/6-31G(d) level of theory using Gaussian 16 software. The GIAO NMR calculation, using PCM/mPW1PW91/6-31G+(d,p) level in CHCl_3_, was applied on the resulting conformers with relative energy less than 2 kcal/mol from the global minimum. The Boltzmann-weighted NMR data of possible candidates and the experimental data were subjected to DP4+ probability analysis with the aid of the Excel sheet provided by Grimblat et al. (Tables S1–S11) [13].

### 3.6. Generation of SARS-CoV-2 Spike Pseudotyped Viruses

SARS-CoV-2 Spike pseudotyped viral particles have recently been reported using HIV-based lentiviral particles [24]. The pseudotyped lentiviral particles with SARS-CoV-2 Spike were then generated with some modifications. In brief, 293T cells were co-transfected with three plasmids, including pcDNA3.1-2019-nCoV-SΔ18 (from RNAi Core, Academia Sinica, Taipei, Taiwan), pCMV d8.91 and pAS3W Fluc, to produce pseudotyped viral particles. The supernatant containing pseudotyped viral particles was collected at 24 h, 36 h, and 48 h post-transfection, following centrifugation at 3000 rpm for 10 min to remove cellular debris. The supernatant was then passed through a 0.45 μm filter and concentrated. The viral particle number was determined by real-time RT-PCR to quantify the RNA copies of FLuc reporter gene.

### 3.7. MTS Assay

MTS assay was performed as previously described [6]. In brief, the THP-1 cells were seeded on 96-well plate at a density of 1 × 10^4^ cells/well and treated with 5 ng/mL of PMA for 48 h. The differentiated THP-1 cells were treated with the tested compounds at the concentration of 25 μM for additional 18 h. The cells were then incubated with MTS solution for 4 h, and the absorbance at 490 nm was measured using a microplate spectrophotometer (Molecular Devices, San Jose, CA, USA).

### 3.8. Compound Screening to Inhibit SARS-CoV-2 Induced-Inflammation

In brief, the human monocytic cell line THP-1 was treated with 5 ng/mL of phorbol 12-myristate 13-acetate (PMA; sigma) to differentiate into macrophages in RPMI-1640 medium with 10% FBS for 24 h. After changing fresh medium, the cells were incubated for further 48 h [25]. The differentiated THP-1 cells were re-seeded into 12 well at a density of 5 × 10^5^ cells per mL, and the differentiated macrophages were infected with SARS-CoV-2 pseudotyped viruses (purchased from RNAi Core, Academia Sinica, Taipei, Taiwan) for 2 h following treated with each tested compound (25 μM) for 18 h. The cell lysates were collected, and the expression level and phosphorylated status of NF-*κ*B were detected by Western analysis.

## 4. Conclusions

Six new polyoxygenated terpenoids, podovirosanes A–F (**1**–**6**), and two known polyketides (**7** and **8**) were isolated from the roots of *F. virosa*. Among them, the polyketides **7** and **8** likely originated from root symbiotic fungi as these two compounds and related analogs were reported from fungi [16,17,18,26]. The present work represents the first report for the non-alkaloid constituents of the title plant that exhibited potent anti-inflammatory activity against SARS-CoV-2-induced inflammation.

## Figures and Tables

**Table 1:** ^1^H NMR spectroscopic data of compounds **1**–**3**, **5**, and **6**.

	1 ^a^	2 ^b^	3 ^b^	5 ^b^	6 ^a^
No.	*δ*_H_ (*J* in Hz)	*δ*_H_ (*J* in Hz)	*δ*_H_ (*J* in Hz)	*δ*_H_ (*J* in Hz)	*δ*_H_ (*J* in Hz)
1	2.42 br d (13.2)	2.16 td (14.2, 4.4)	2.52 td (14.0, 4.3)	1.98 m	2.25 dd (14.5, 3.7)
	2.28 td (13.2, 5.4)	1.88 m	1.59 m	1.98 m	1.95 d (14.5, 2.0)
2	2.10 m	1.88 m	2.01 m	2.24 m	4.00 ddd (3.7, 3.0, 2.0)
	2.05 m	1.73 m	1.85 m	1.50 m	
3	3.81 br s	3.40 dd (11.7, 3.4)	3.62 br s	3.88 t (8.0)	3.63 br d (3.0)
5	2.94 br s	0.91 d (2.4)	1.29 d (3.6)	1.56 d (6.2)	2.39 dd (4.6, 2.2)
6	5.90 br d (9.8)	4.21 m	4.15 m	4.44 t (6.2)	5.85 dd (12.0, 4.6)
7	6.60 dd (9.8, 2.9)	4.72 d (4.4)	4.71 d (4.3)	4.72 d (6.2)	6.61 dd (12.0, 2.2)
11	6.93 s	6.67 s	6.73 s	6.58 s	6.58 s
14	6.89 s	7.12 s	7.11 s	6.85 s	6.96 s
15	2.18 s	2.22 s	2.21 s	2.18 s	2.18 s
18	1.19 s	1.14 s	1.11 s	1.03 s	1.03 s
19	4.37 d (12.2)	1.14 s	1.17 s	1.10 s	1.25 s
	4.04 dd (12.2, 1.7)				
20		4.07 d (8.5)	4.11 d (8.5)	2.98 d (16.3)	2.95 d (14.0)
		2.72 d (8.5)	2.75 d (8.5)	2.55 d (16.3)	2.68 d (14.0)
OMe	3.88 s	3.86 s	3.86 s	3.80 s	3.84 s

^a^ Spectra recorded at 400 MHz in CDCl_3_; ^b^ Spectra recorded at 500 MHz in CDCl_3_.

**Table 2:** ^13^C NMR spectroscopic data of compounds **1**–**6**.

	1 ^a^	2 ^b^	3 ^b^	4 ^c^	5 ^b^	6 ^a^
No.	*δ*_C_ (mult.)	*δ*_C_ (mult.)	*δ*_C_ (mult.)	*δ*_C_ (mult.)	*δ*_C_ (mult.)	*δ*_C_ (mult.)
1	27.9 (CH_2_)	25.9 (CH_2_)	20.5 (CH_2_)	33.1 (CH_2_)	30.3 (CH_2_)	39.1 (CH_2_)
2	28.9 (CH_2_)	27.0 (CH_2_)	24.9 (CH_2_)	28.6 (CH_2_)	25.7 (CH_2_)	72.7 (CH)
3	74.5 (CH)	78.5 (CH)	74.9 (CH)	78.0 (CH)	71.3 (CH)	77.7 (CH)
4	36.6 (C)	39.1 (C)	37.9 (C)	39.7 (C)	36.1 (C)	37.3 (C)
5	39.0 (CH)	56.8 (CH)	50.8 (CH)	44.8 (CH)	58.7 (CH)	50.0 (CH)
6	122.8 (CH)	68.9 (CH)	69.1 (CH)	23.0 (CH_2_)	78.5 (CH)	127.8 (CH)
7	131.2 (CH)	72.9 (CH)	73.0 (CH)	33.0 (CH_2_)	77.5 (CH)	131.4 (CH)
8	125.2 (C)	125.9 (C)	125.8 (C)	141.0 (C)	125.6 (C)	128.5 (C)
9	134.8 (C)	145.1 (C)	145.5 (C)	76.8 (C)	131.9 (C)	134.2 (C)
10	47.5 (C)	39.2 (C)	39.0 (C)	43.6 (C)	80.4 (C)	79.7 (C)
11	108.1 (CH)	101.6 (CH)	101.8 (CH)	67.3 (CH)	110.9 (CH)	112.1 (CH)
12	157.4 (C)	158.3 (C)	158.3 (C)	78.4 (CH)	157.7 (C)	157.1 (C)
13	125.9 (C)	124.6 (C)	124.4 (C)	45.1 (C)	124.3 (C)	124.9 (C)
14	129.0 (CH)	129.0 (CH)	128.9 (CH)	126.3 (CH)	128.7 (CH)	132.2 (CH)
15	15.8 (CH_3_)	15.9 (CH_3_)	15.9 (CH_3_)	145.6 (CH)	15.9 (CH_3_)	15.7 (CH_3_)
16				114.0 (CH_2_)		
17				25.2 (CH_3_)		
18	19.3 (CH_3_)	28.7 (CH_3_)	28.0 (CH_3_)	29.4 (CH_3_)	23.1 (CH_3_)	28.3 (CH_3_)
19	75.4 (CH_2_)	15.4 (CH_3_)	22.2 (CH_3_)	16.7 (CH_3_)	23.8 (CH_3_)	22.6 (CH_3_)
20	174.5 (C)	65.8 (CH_2_)	65.9 (CH_2_)	18.0 (CH_3_)	44.1 (CH_2_)	51.0 (CH_2_)
OMe	55.4 (CH_3_)	55.5 (CH_3_)	55.5 (CH_3_)		55.3 (CH_3_)	55.4 (CH_3_)

^a^ Spectra recorded at 100 MHz in CDCl_3_; ^b^ Spectra recorded at 125 MHz in CDCl_3_. ^c^ Spectra recorded at 100 MHz in pyridine-*d*_5_.

**Table 3:** DP4+ probabilities for possible candidates of compounds **1**, **2**, and **6**.

	DP4+ (%) ^a^							
	Candidates of 1		Candidates of 2		Candidates of 6			
	3β-OH	3α-OH	6β-OH	6α-OH	2α,10α-OH	2β,10α-OH	2α,10β-OH	2β,10β-OH
H	100.00%	0%	100.00%	0%	100.00%	0%	0%	0%
C	100.00%	0%	100.00%	0%	100.00%	0%	0%	0%
All data	100.00%	0%	100.00%	0%	100.00%	0%	0%	0%

^a^ The NMR data were calculated at PCM/mPW1PW91/6-31G+(d,p)//B3LYP/6-31G(d).

## Data Availability

The data used to support the findings of this study are available from the corresponding author upon request.

## Supplementary Materials

The following are available online at https://www.mdpi.com/article/10.3390/molecules27238548/s1, Figures S1–S47: NMR (1D and 2D) and MS spectra of compounds **1**–**6**, Figures S46–S49: structures of possible candidates for DP4+ analysis, Tables S1–S11: conformers and Boltzmann populations of candidate structures in DP4+ analysis. Table S12: ^1^H NMR spectroscopic data of compounds **1**–**6**.
