# Supplementary material for: Polyoxygenated Terpenoids and Polyketides from the Roots of Flueggea virosa and Their Inhibitory Effect against SARS-CoV-2-Induced Inflammation
Source: Molecules. 2022 Dec 5;27(23):8548. doi: 10.3390/molecules27238548 (PMC9737494; doi:10.3390/molecules27238548)
Supplement: Supplementary file 1 [file molecules-27-08548-s001.zip › molecules-2046091-supplementary.pdf]

Polyoxygenated Terpenoids and Polyketides from the Roots of *Flueggea virosa* and Their Inhibitory Effect against SARS-CoV-2-Induced Inflammation

Ju-Chien Cheng [1,†], Yi-Ju Chen [2,†], Chi-Wen Chuang [3], Ya-Hsuan Chao [1], Hui-Chi Huang [4], Chia-Chi Lin [4] and Chih-Hua Chao [3,5,*]

1 Department of Medical Laboratory Science and Biotechnology, China Medical University, Taichung 40402, Taiwan
2 Core Facility Center, Office of Research and Development, Taipei Medical University, Taipei 110301, Taiwan
3 School of Pharmacy, China Medical University, Taichung 406040, Taiwan
4 Department of Chinese Pharmaceutical Sciences and Chinese Medicine Resources, China Medical University, Taichung 40402, Taiwan
5 Chinese Medicine Research and Development Center, China Medical University Hospital, Taichung 404332, Taiwan
* Correspondence: chchao@mail.cmu.edu.tw; Tel: +886-4-22053366 (ext. 5157); Fax: +886-4-22078083
† These authors contributed equally to this work.

Figure S1. (+)-HR-EIMS spectrum of **1**.
Figure S2. ^{1}H NMR spectrum of **1**.
Figure S3. ^{13}C NMR spectrum of **1**.
Figure S4. DEPT spectrum of **1**.
Figure S5. HSQC spectrum of **1**.
Figure S6. HMBC spectrum of **1**.
Figure S7. COSY spectrum of **1**.
Figure S8. NOESY spectrum of **1**.
Figure S9. (–)-HR-APCIMS spectrum of **2**.
Figure S10. ^{1}H NMR spectrum of **2**.

Figure S11. ^{13}C NMR spectrum of **2**.
Figure S12. DEPT spectrum of **2**.
Figure S13. HSQC spectrum of **2**.
Figure S14. HMBC spectrum of **2**.
Figure S15. COSY spectrum of **2**.
Figure S16. NOESY spectrum of **2**.
Figure S17. (–)-HR-ESIMS spectrum of **3**.
Figure S18. ^{1}H NMR spectrum of **3**.
Figure S19. ^{13}C NMR spectrum of **3**.
Figure S20. HSQC spectrum of **3**.
Figure S21. HMBC spectrum of **3**.
Figure S22. COSY spectrum of **3**.
Figure S23. NOESY spectrum of **3**.
Figure S24. (–)-HR-ESIMS spectrum of **4**.
Figure S25. ^{1}H NMR spectrum of **4**.
Figure S26. ^{13}C NMR spectrum of **4**.
Figure S27. DEPT spectrum of **4**.
Figure S28. HSQC spectrum of **4**.
Figure S29. HMBC spectrum of **4**.
Figure S30. COSY spectrum of **4**.
Figure S31. NOESY spectrum of **4**.
Figure S32. (–)-HR-APCIMS spectrum of **5**.
Figure S33. ^{1}H NMR spectrum of **5**.
Figure S34. ^{13}C NMR spectrum of **5**.
Figure S35. DEPT spectrum of **5**.
Figure S36. HSQC spectrum of **5**.
Figure S37. HMBC spectrum of **5**.
Figure S38. COSY spectrum of **5**.
Figure S39. NOESY spectrum of **5**.
Figure S40. (+)-HR-ESIMS spectra of **6**.
Figure S41. ^{1}H NMR spectrum of **6**.
Figure S42. ^{13}C NMR spectrum of **6**.
Figure S43. DEPT spectrum of **6**.
Figure S44. HSQC spectrum of **6**.
Figure S45. HMBC spectrum of **6**.
Figure S46. COSY spectrum of **6**.
Figure S47. NOESY spectrum of **6**.
Figure S48. Possible candidates of compounds **1**, **2**, and **6** for DP4+ probability

analysis.

Figure S49. H-6/H-7 distance of 6*α*-OH and 6β-OH possible candidates of compound **2.** Conformers were generated by MM2 energy minimizations.

Table S1. DP4+ analysis table for compound **1** (isomer 1: 3β-OH; isomer 2: 3α-OH).

Table S2. Conformers and Boltzmann populations of 3α-OH isomer of **1**.

Table S3. Conformers and Boltzmann populations of 3β-OH isomer of **1**.

Table S4. DP4+ analysis table for compound **2** (isomer 1: 6α-OH; isomer 2: 6β-OH).

Table S5. Conformers and Boltzmann populations of 6α-OH isomer of **2**.

Table S6. Conformers and Boltzmann populations of 6β-OH isomer of **2**.

Table S7. DP4+ analysis table for compound **6** (isomer 1: 2α,10α-OH; isomer 2: 2β,10α-OH; isomer 3: 2α,10β-OH; isomer 4: 2β,10β-OH).

Table S8. Conformers and Boltzmann populations of 2α,10α-OH isomer of **6**.

Table S9. Conformers and Boltzmann populations of 2β,10α-OH isomer of **6**.

Table S10. Conformers and Boltzmann populations of 2α,10β-OH isomer of **6**.

Table S11. Conformers and Boltzmann populations of 2β,10β-OH isomer of **6**.

Table S12. ^{1}H NMR spectroscopic data of compounds **1**–**6**.

```
LIST: hei2124_fvcl17k1g3-c3          30-Dec-18 REG   : 02:58.5     #9
Samp:                                           Start : 09:24:49  9104
Mode: EI +VE +LMR  ESCAN (EXP) UP HR NRM
Oper:                                           Inlet :
Limt: (   0)     .    .
    : (422) C24.H38.O6
Peak: 1000.00 mmu   R+D: -2.0 > 60.0
Data: +/519>575  (CMASS : converted |CMASS : converted |CMASS : conve

                   0                  (mmu)
    Mass   Intensity     %RA   Flags Delta  R+D  Composition
314.1524      428258   95.72   #      -0.6  9.0  C19.H22.O4
```

Figure S1. (+)-HR-EIMS spectrum of **1**.

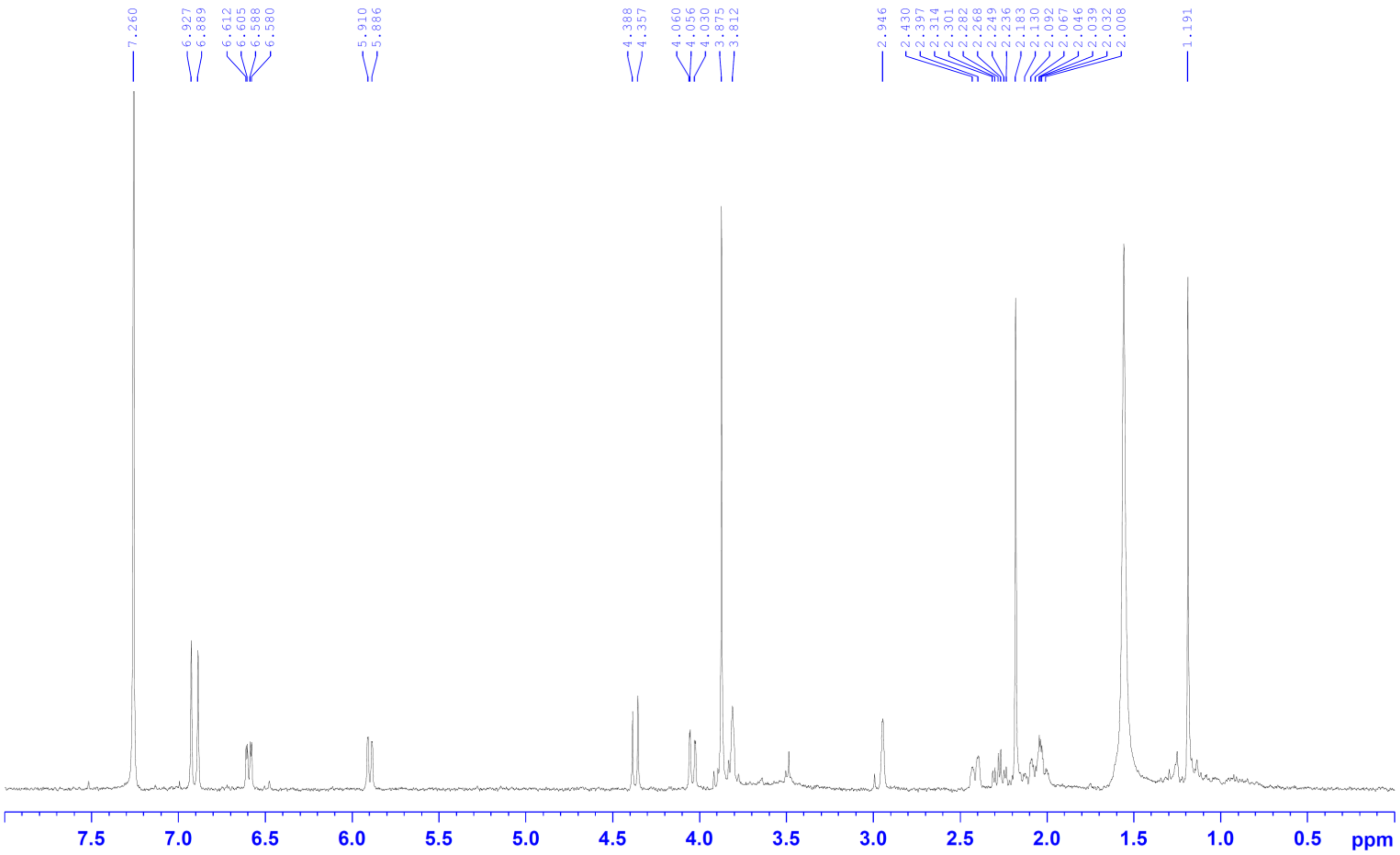

Figure S2. ^{1}H NMR spectrum of **1**.

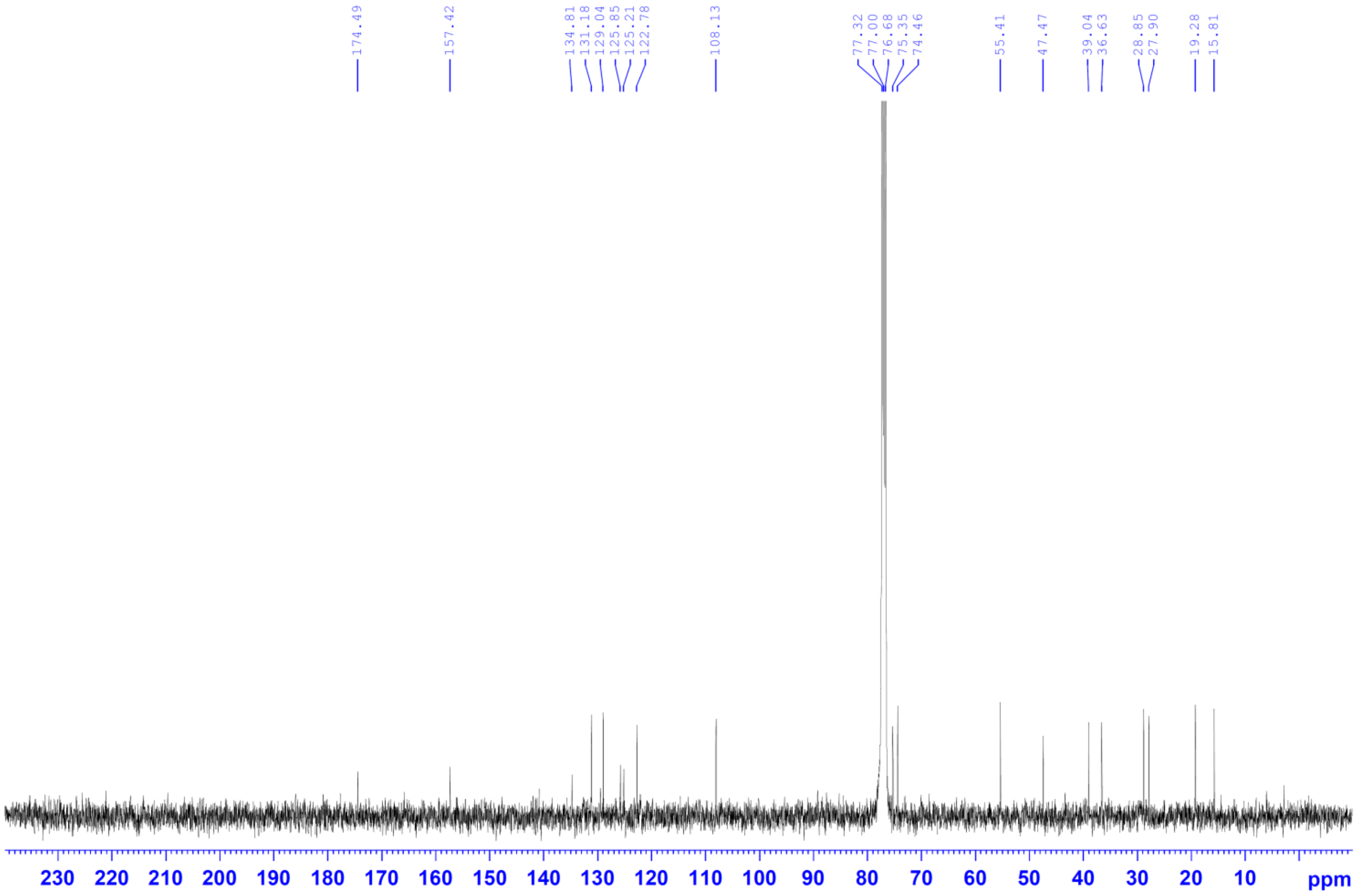

Figure S3. ^{13}C NMR spectrum of **1**.

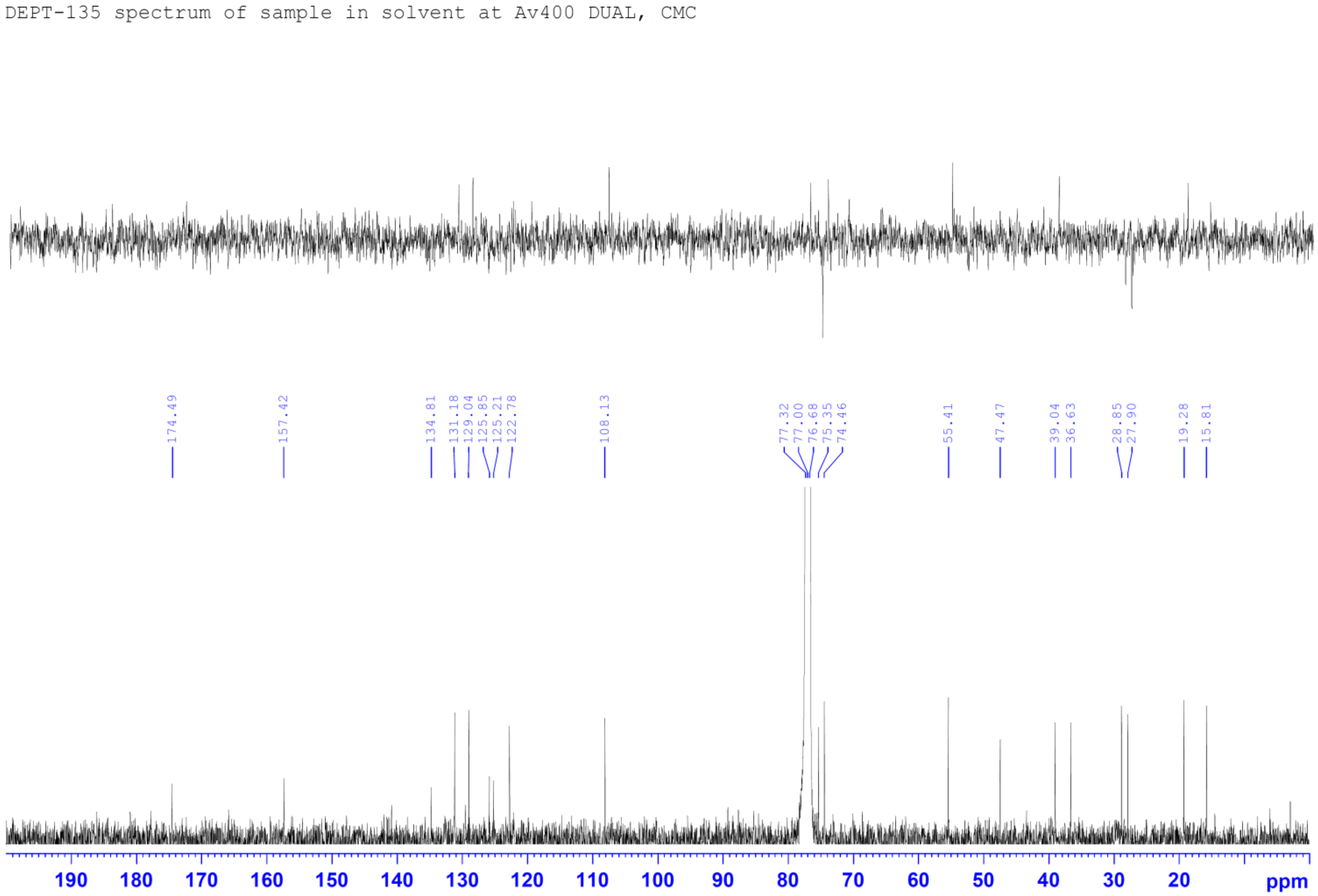

Figure S4. DEPT spectrum of **1**.

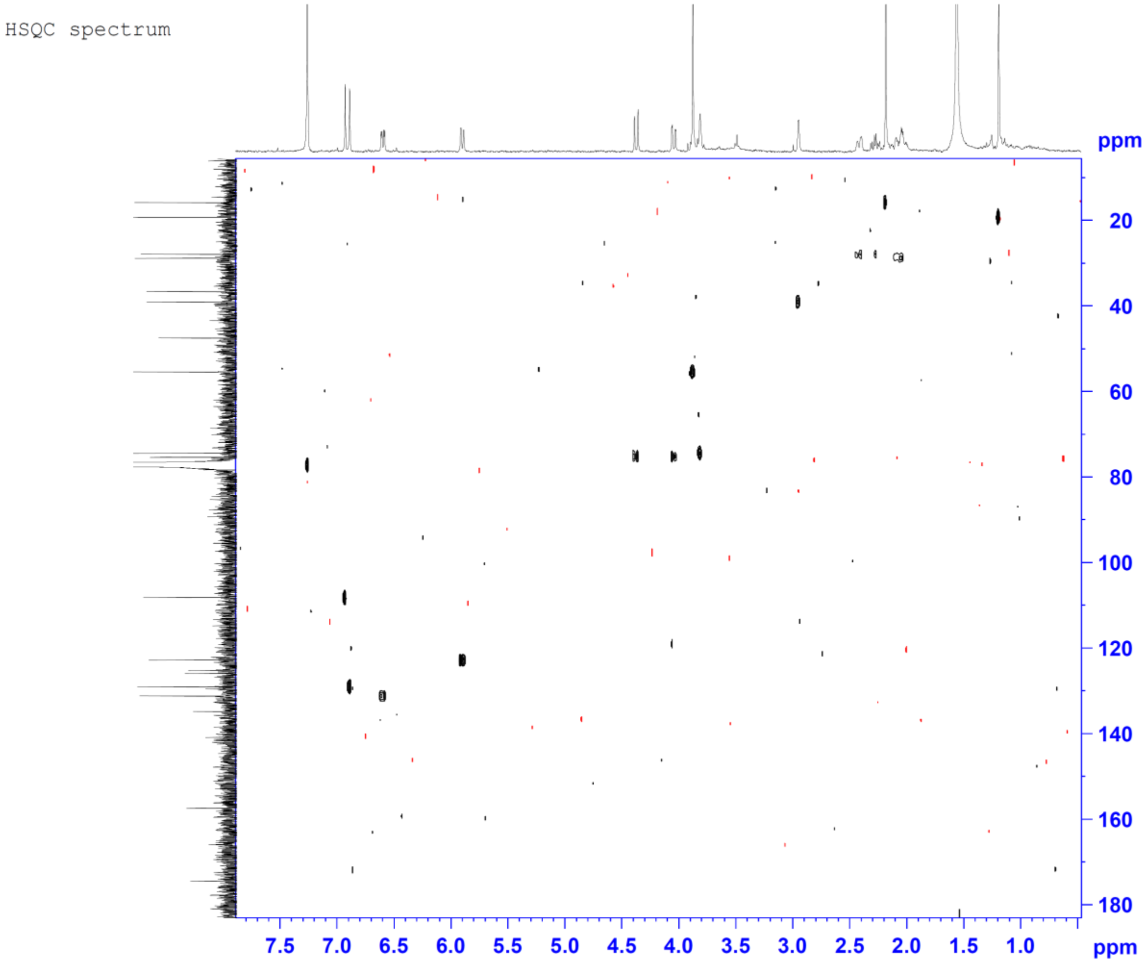

Figure S5. HSQC spectrum of **1**.

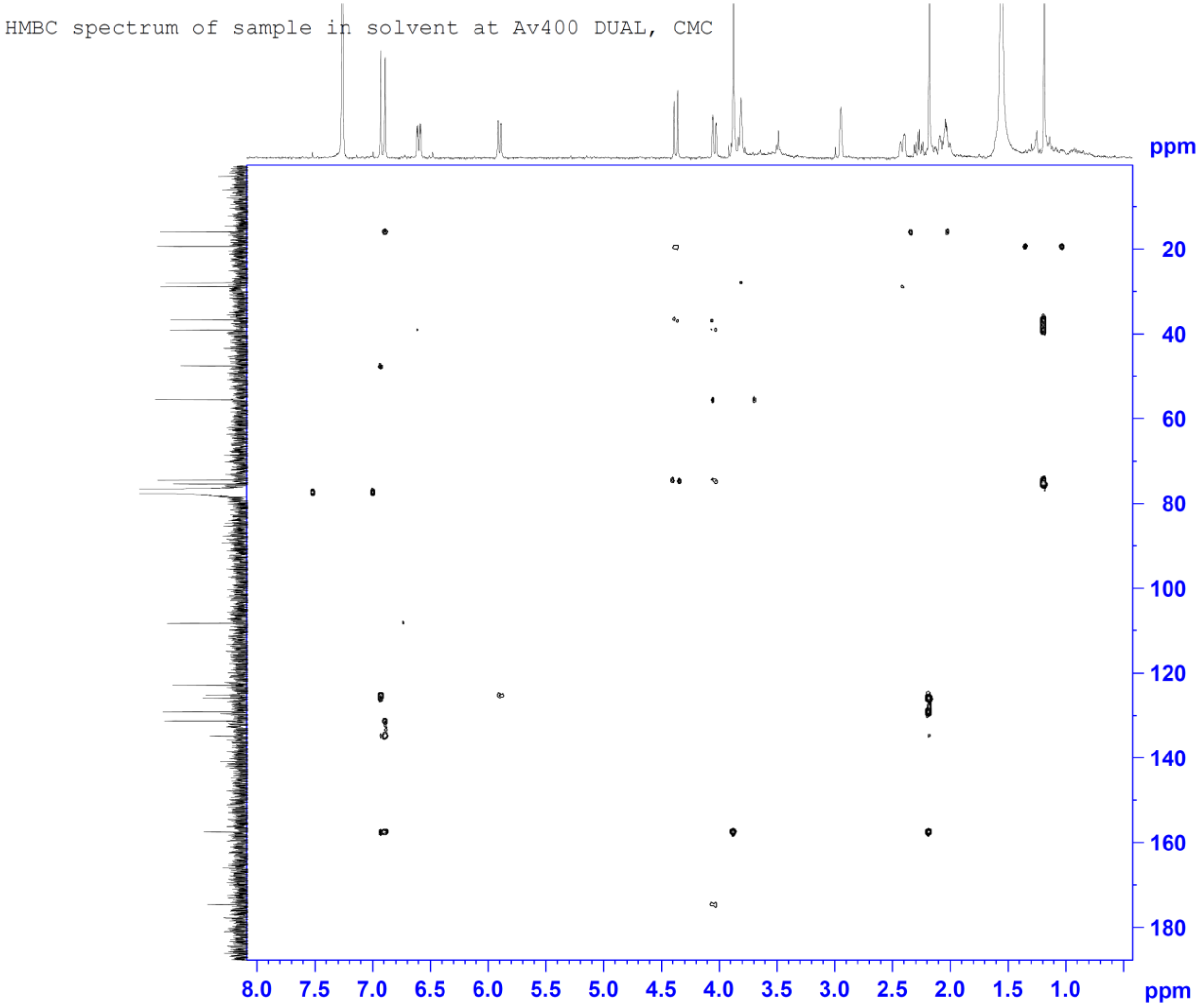

Figure S6. HMBC spectrum of **1**.

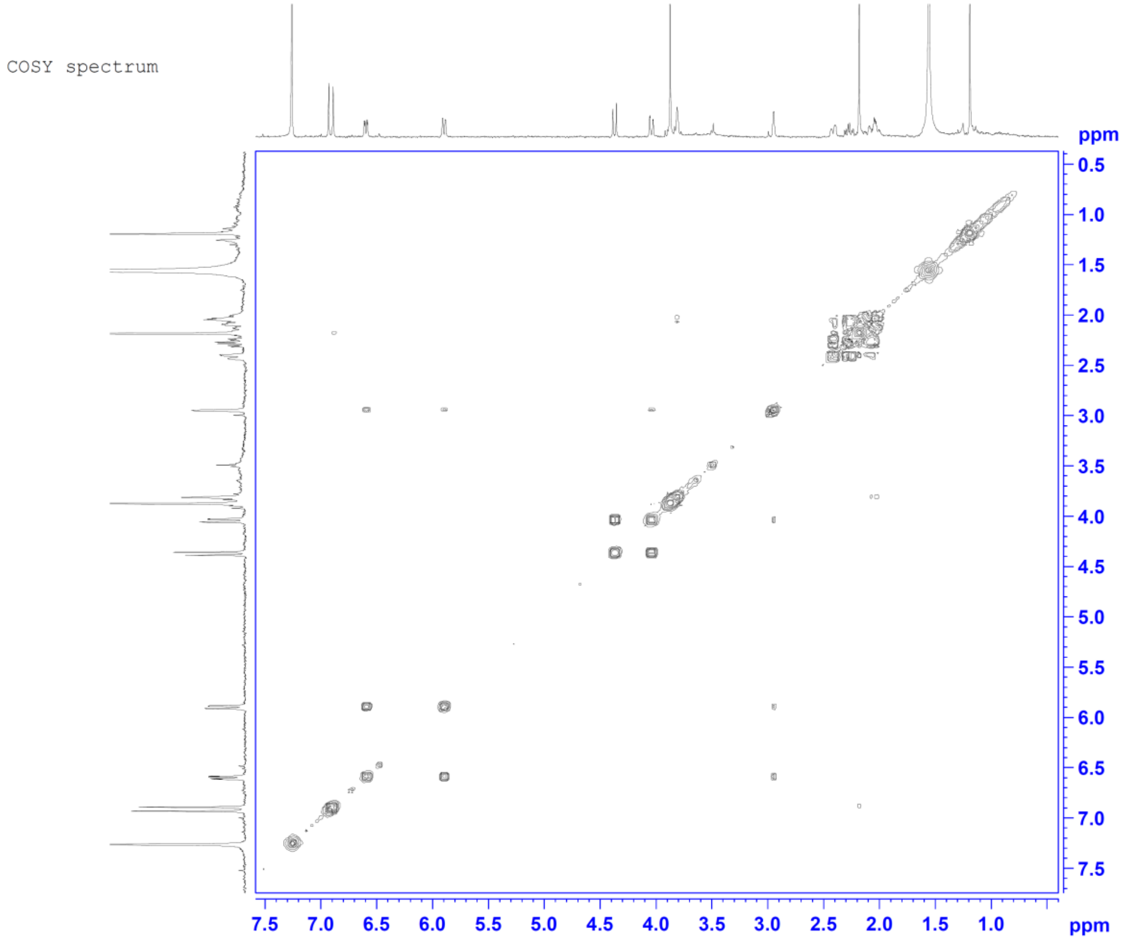

Figure S7. COSY spectrum of **1**.

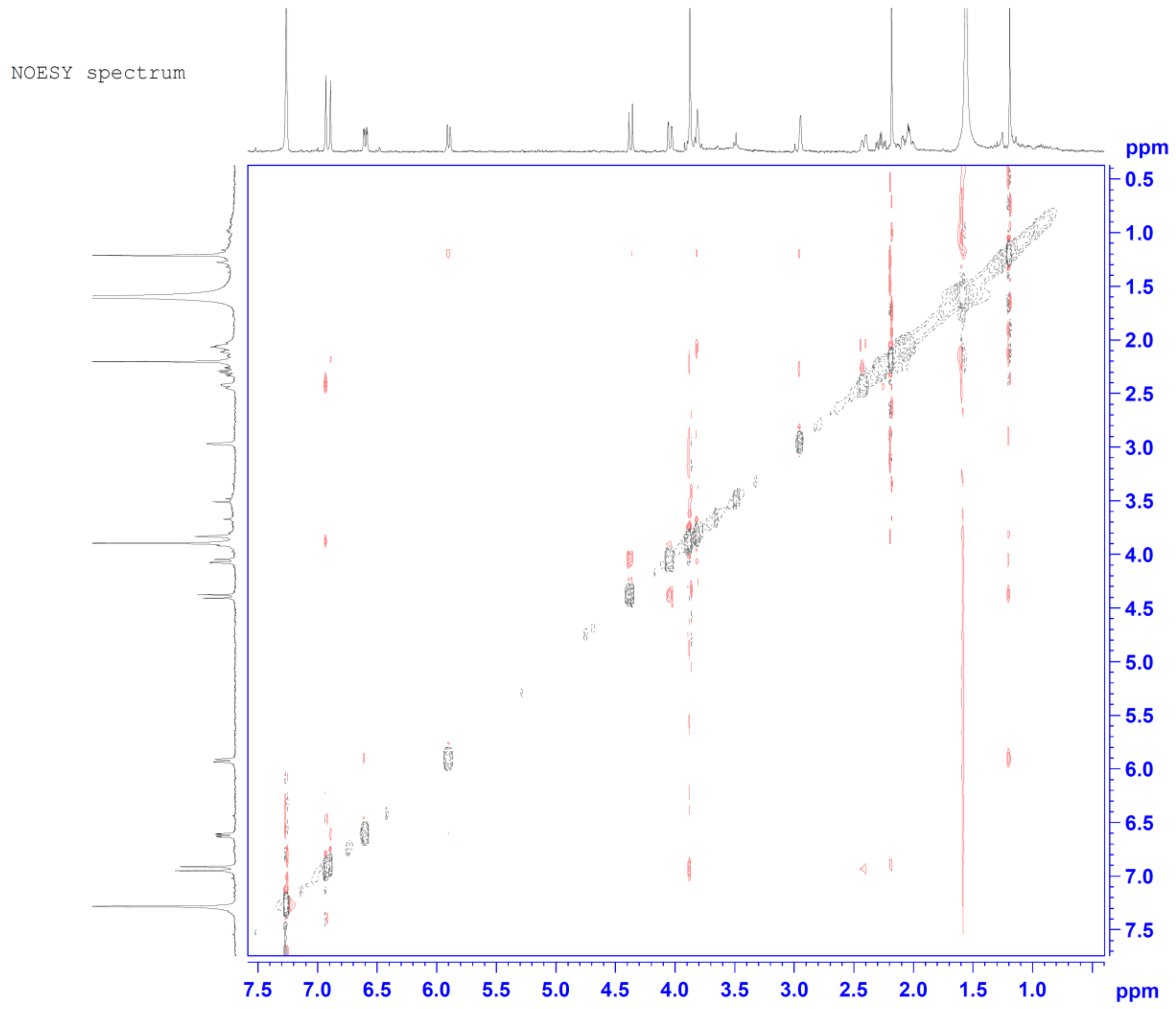

Figure S8. NOESY spectrum of **1**.

F:\Exp_data\...\85-FV-Cl-17K1D2-APCI-H 2017/9/26 下午 05:18:08

85-FV-Cl-17K1D2-APCI-H#1-20 RT: 0.01-0.53 AV: 20
T: FTMS - p APCI corona Full ms [250.00-400.00]
m/z= 317.1718-317.1777

Isotope	Min	Max
O-16	0	4
C-12	0	19
H-1	0	27

Charge 1
Mass tolerance 1000.00 ppm
Nitrogen rule not used
RDB equiv -1.00-100.00
max results 1

m/z	Intensity	Relative	Theo. Mass	Delta (ppm)	Composition
317.1748	113607.6	100.00	317.1747	0.15	$C_{19}H_{25}O_{4}$

Figure S9. (–)-HR-APCIMS spectrum of **2**.

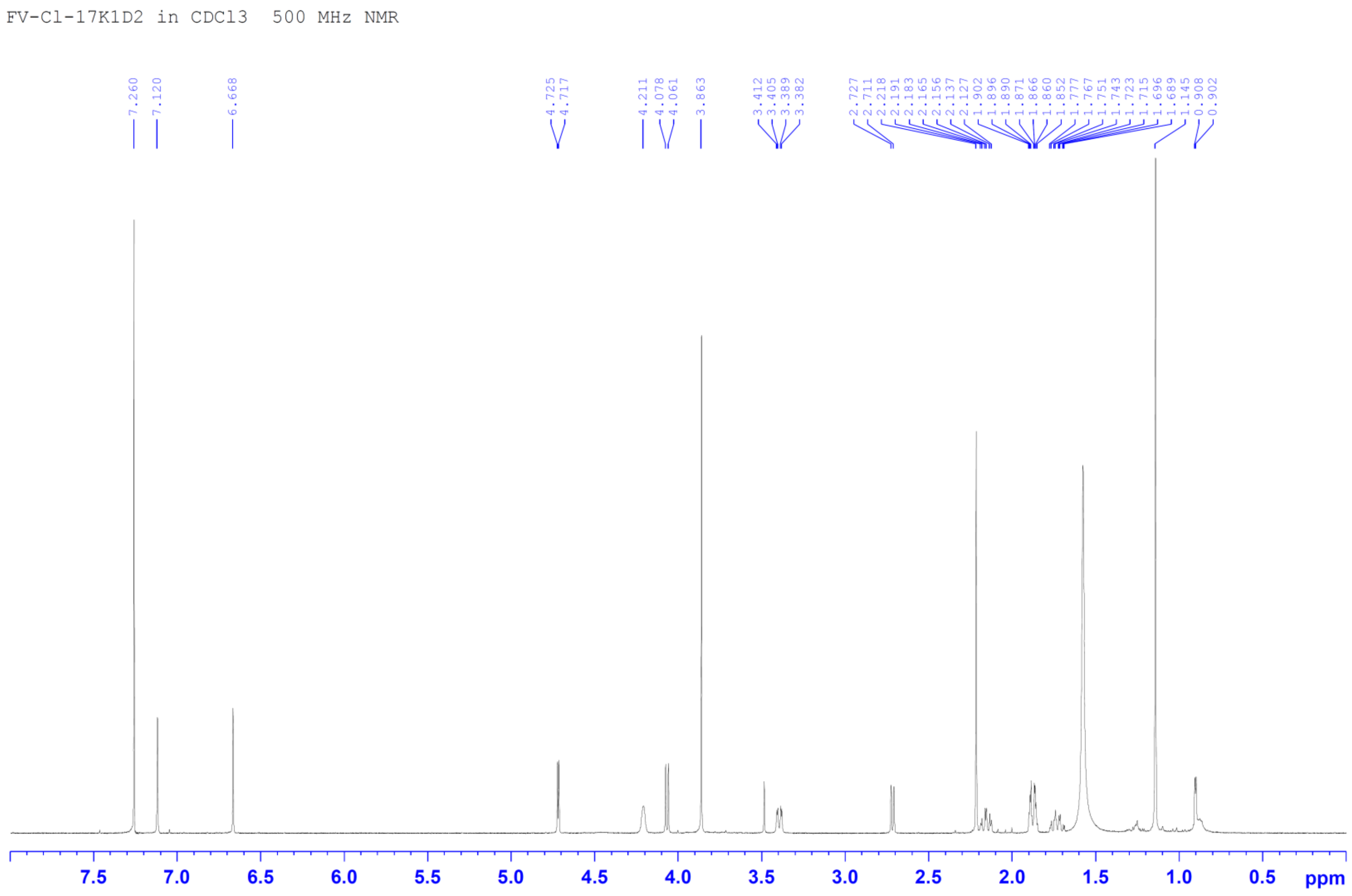

Figure S10. ^{1}H NMR spectrum of **2**.

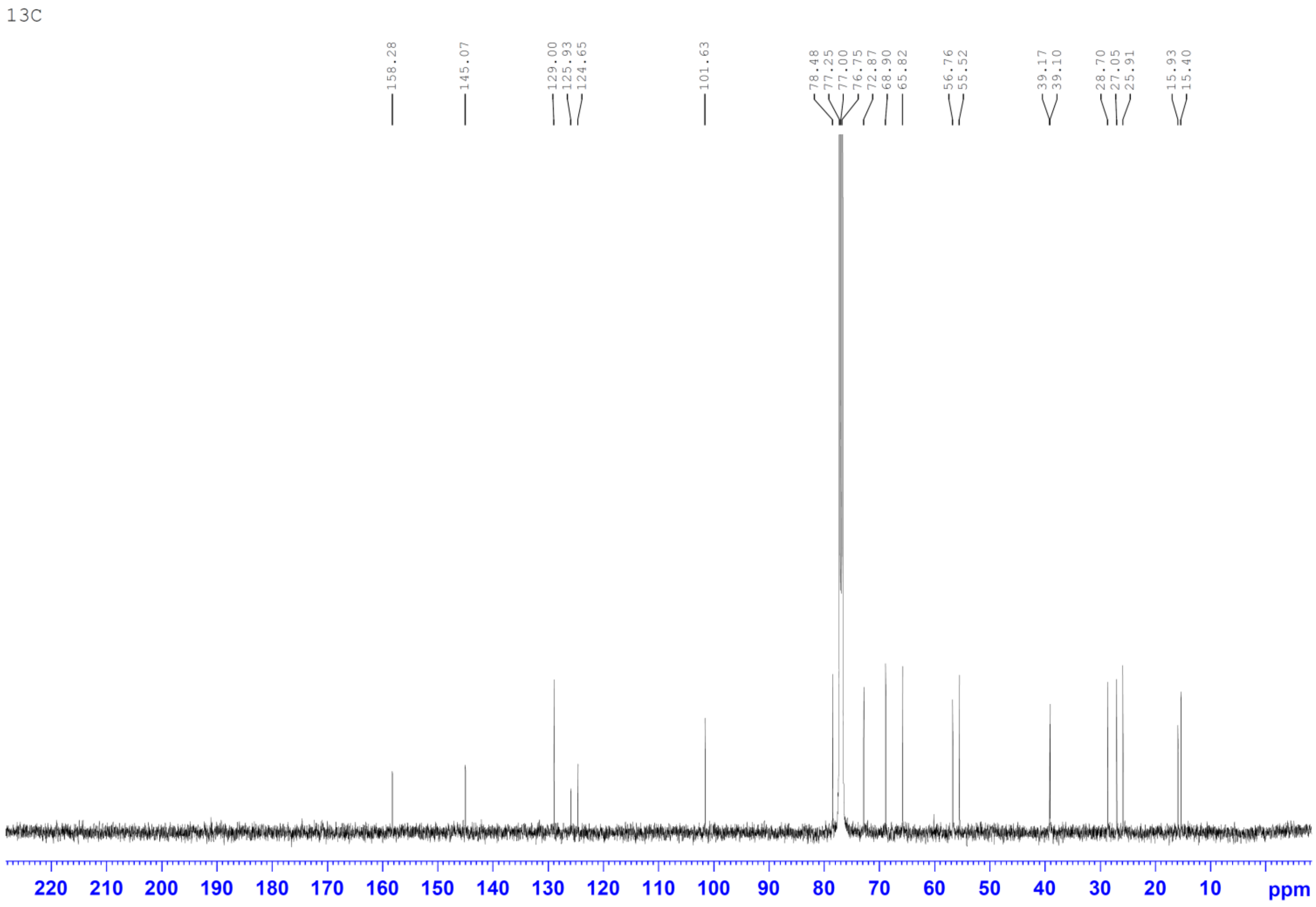

Figure S11. ^{13}C NMR spectrum of **2**.

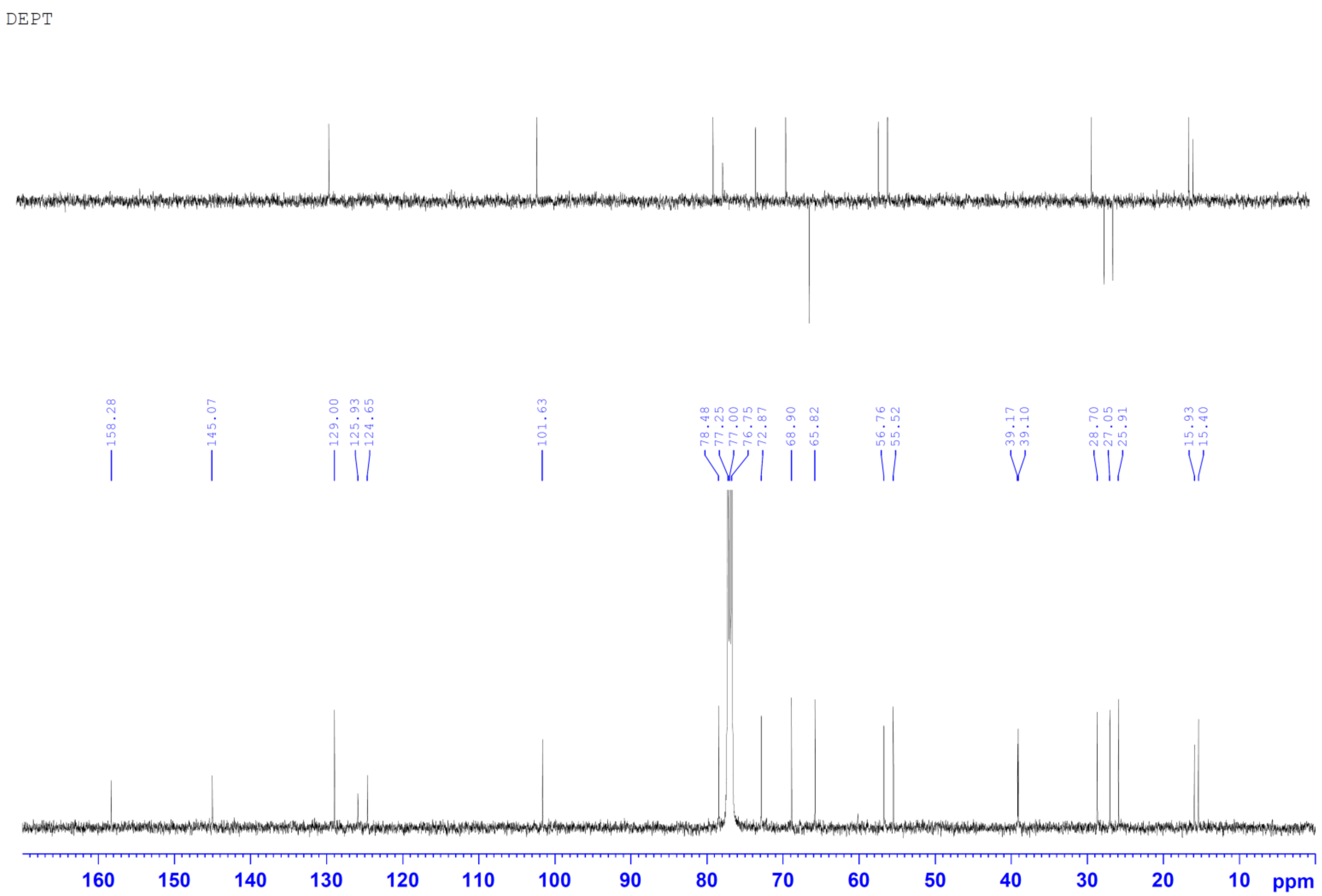

Figure S12. DEPT spectrum of **2**.

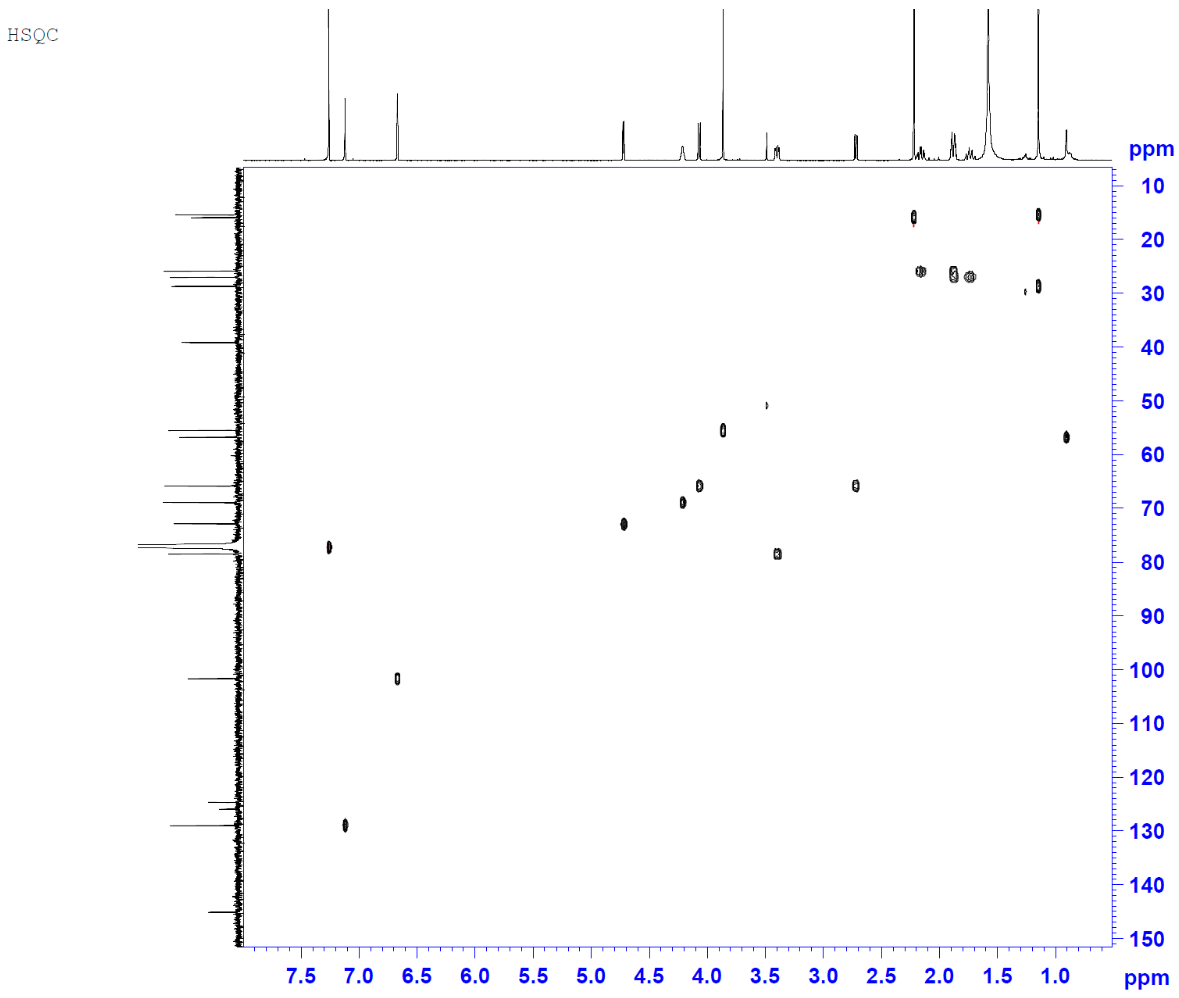

Figure S13. HSQC spectrum of **2**.

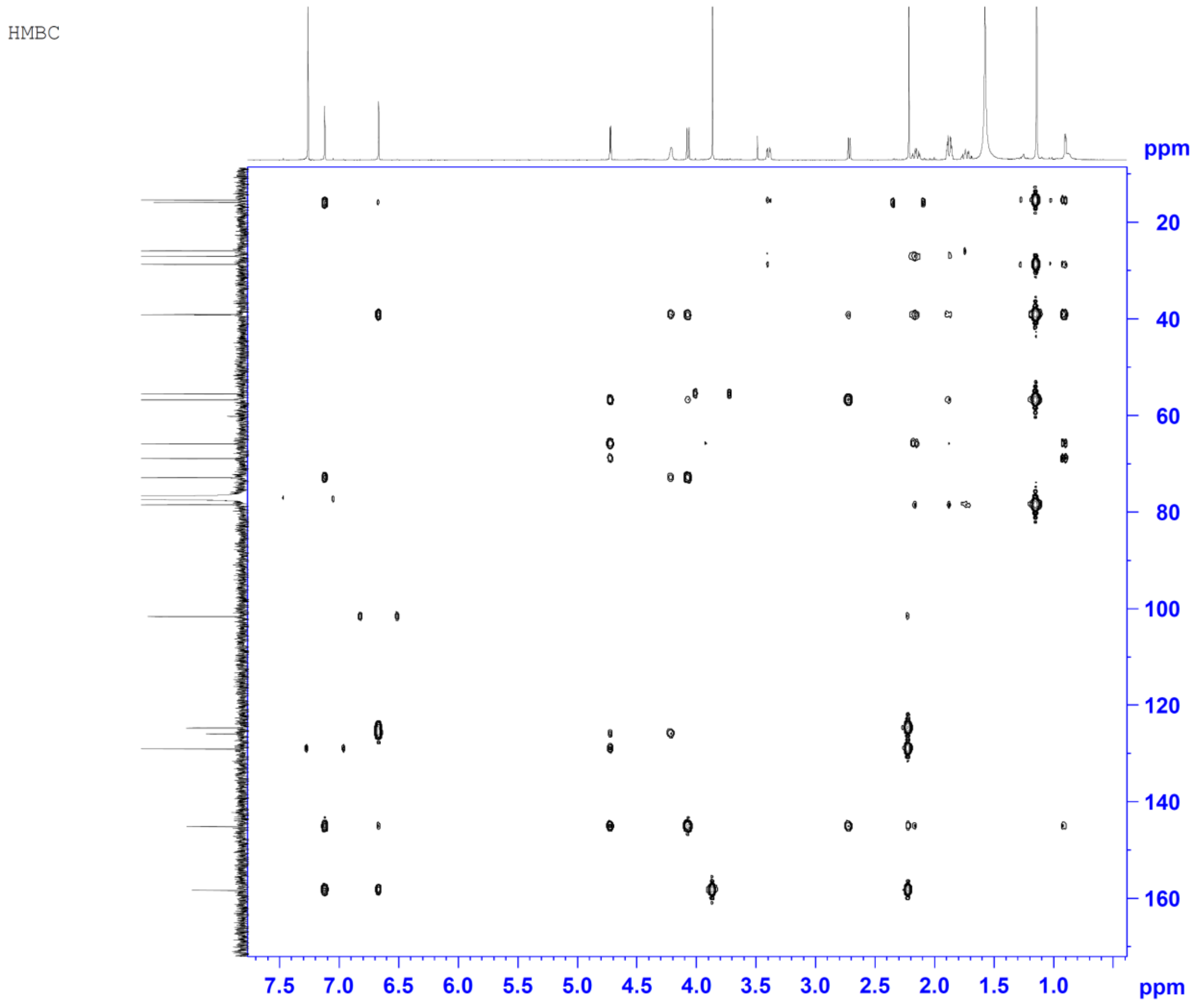

Figure S14. HMBC spectrum of **2**.

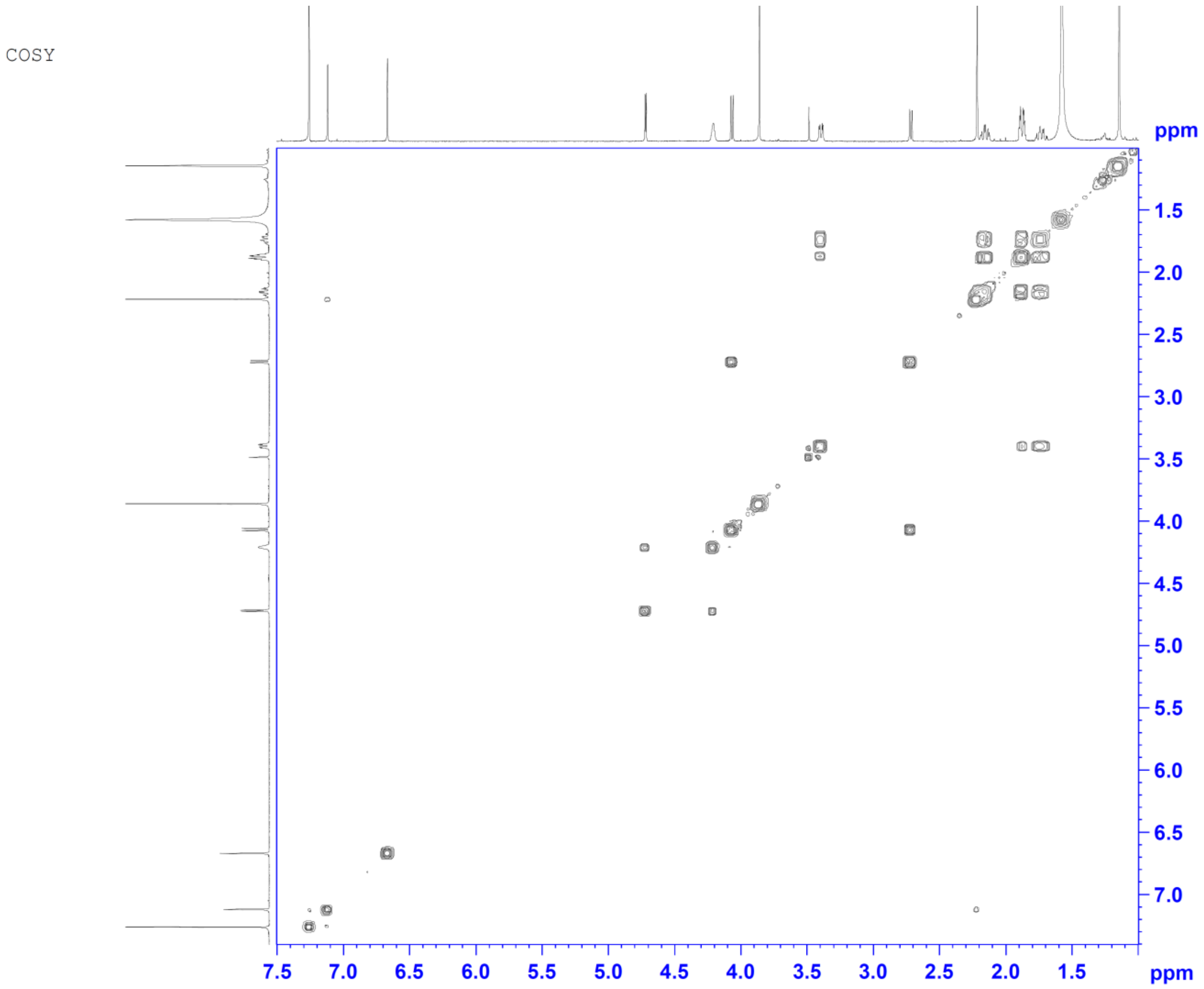

Figure S15. COSY spectrum of **2**.

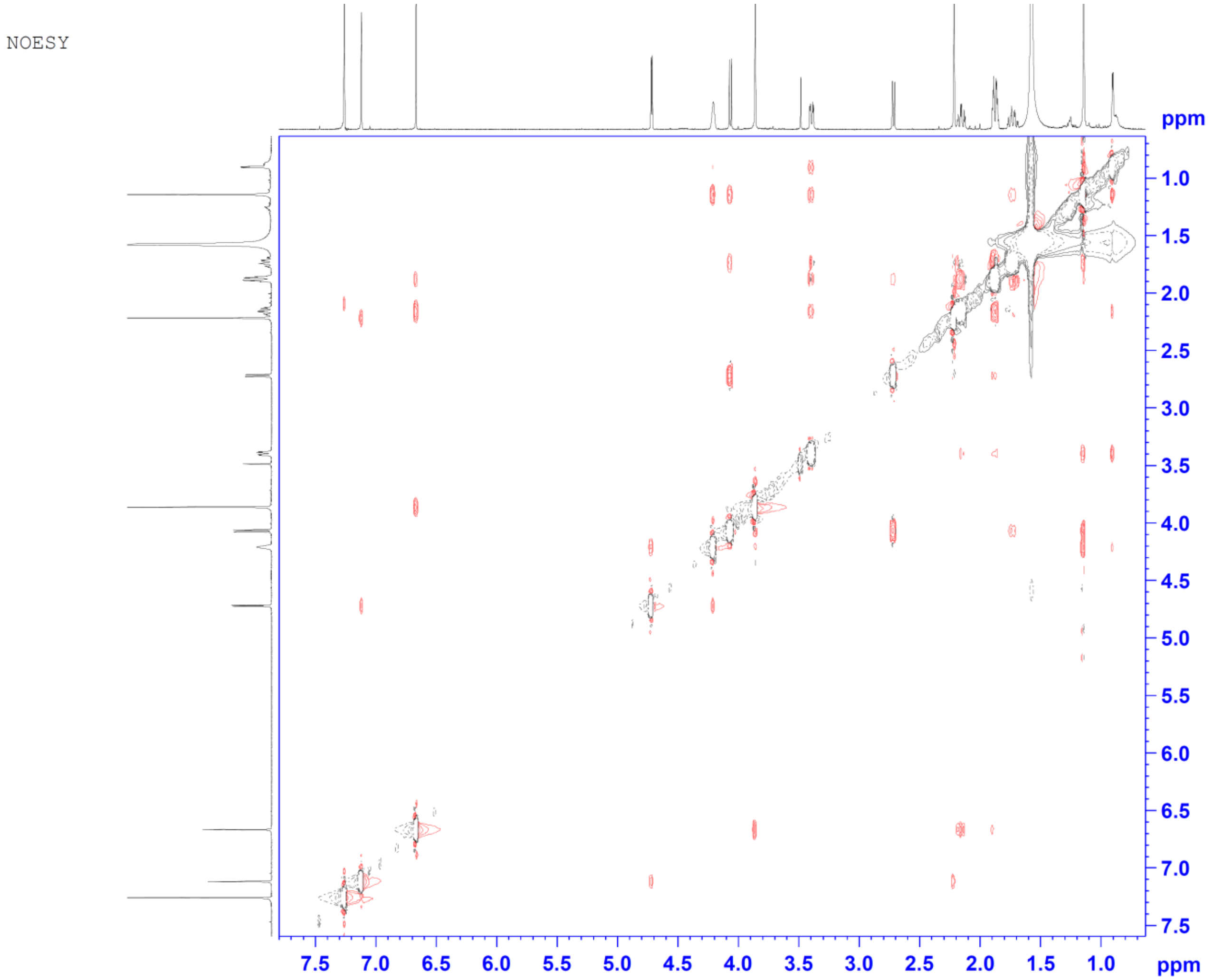

Figure S16. NOESY spectrum of **2**.

F:\Exp_data\...\51-FV-Cl-17J3D-H 2017/9/20 上午 11:31:15

51-FV-Cl-17J3D-H#1-20 RT: 0.01-0.53 AV: 20
T: FTMS - p ESI Full ms [310.00-325.00]
m/z= 317.1416-317.2115

Isotope	Min	Max
O-16	0	4
C-12	0	19
H-1	0	27

Charge 1
Mass tolerance 1000.00 ppm
Nitrogen rule not used
RDB equiv -1.00-100.00
max results 1

m/z	Intensity	Relative	Theo. Mass	Delta (ppm)	Composition
317.1753	3564591.0	100.00	317.1747	1.90	$C_{19}H_{25}O_4$

Figure S17. (–)-HR-ESIMS spectrum of **3**.

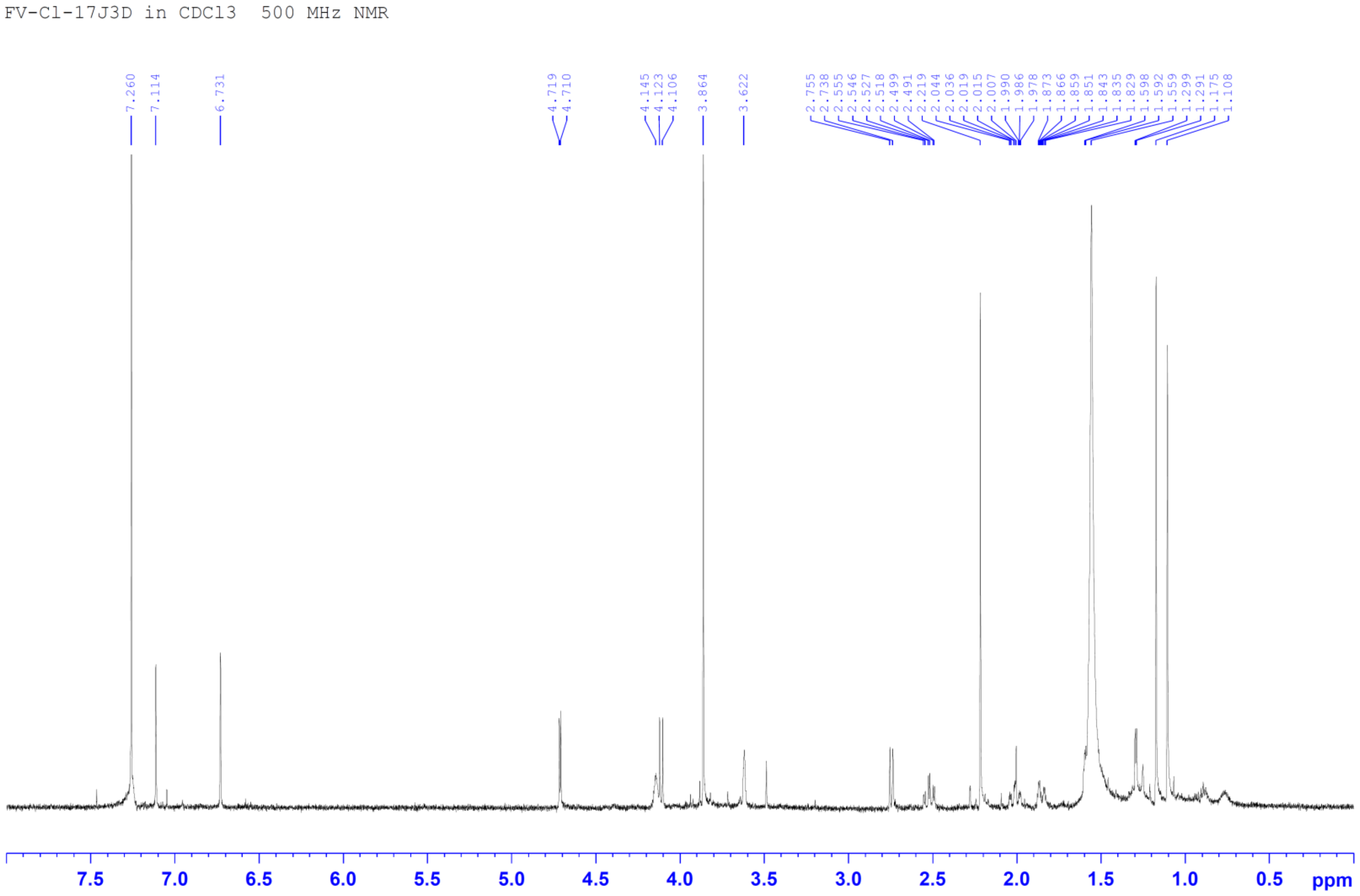

Figure S18. ^{1}H NMR spectrum of **3**.

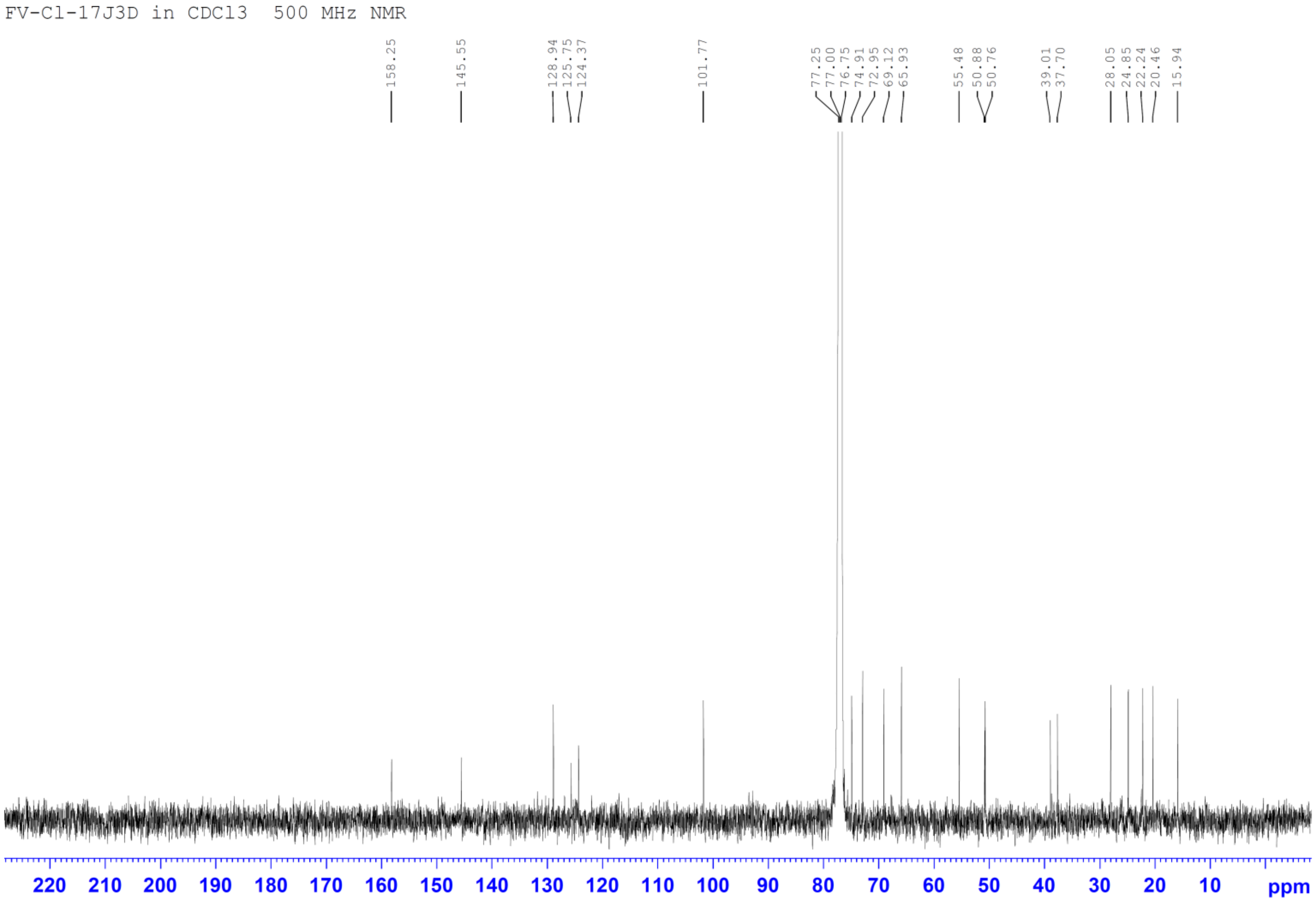

Figure S19. ^{13}C NMR spectrum of **3**.

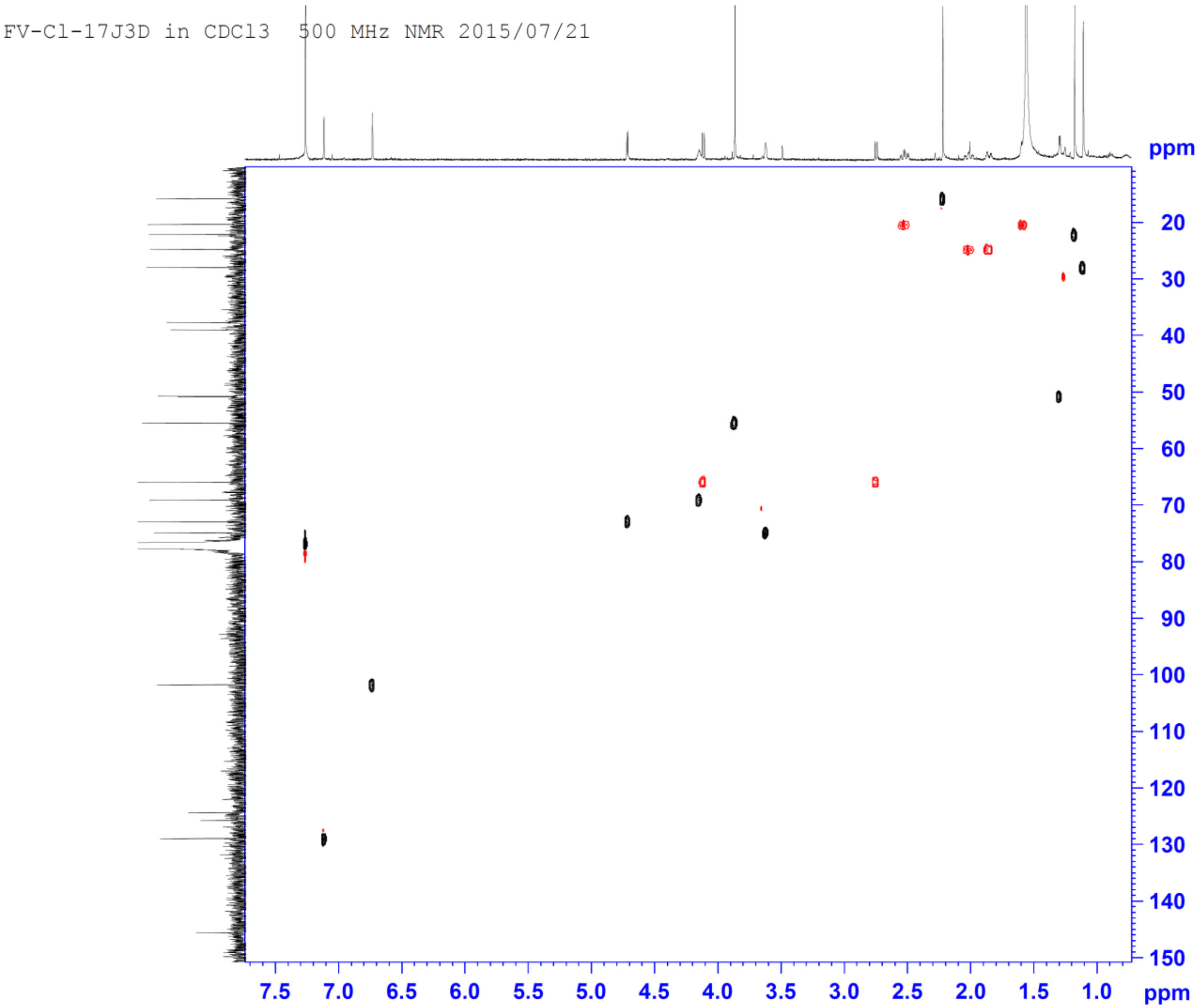

Figure S20. HSQC spectrum of **3**.

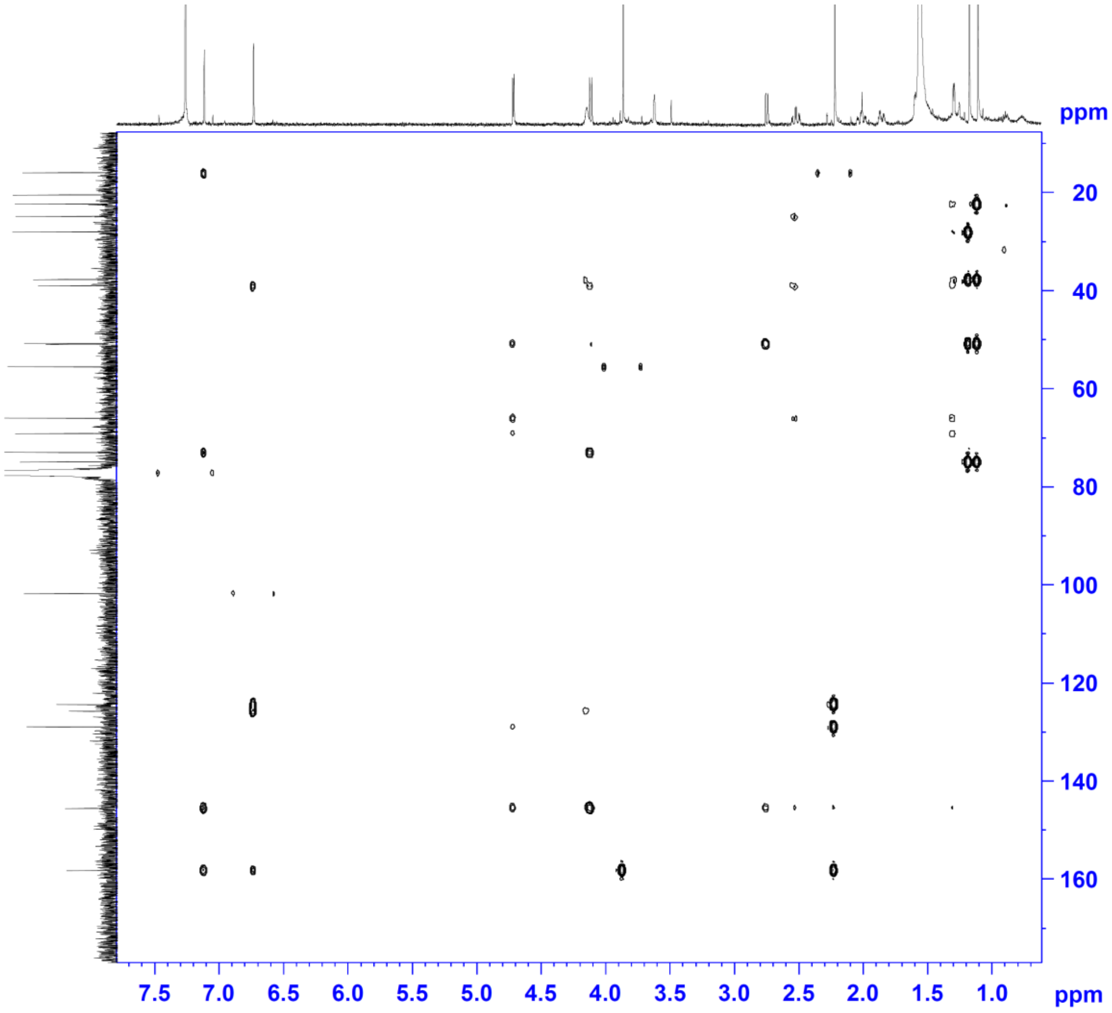

Figure S21. HMBC spectrum of **3**.

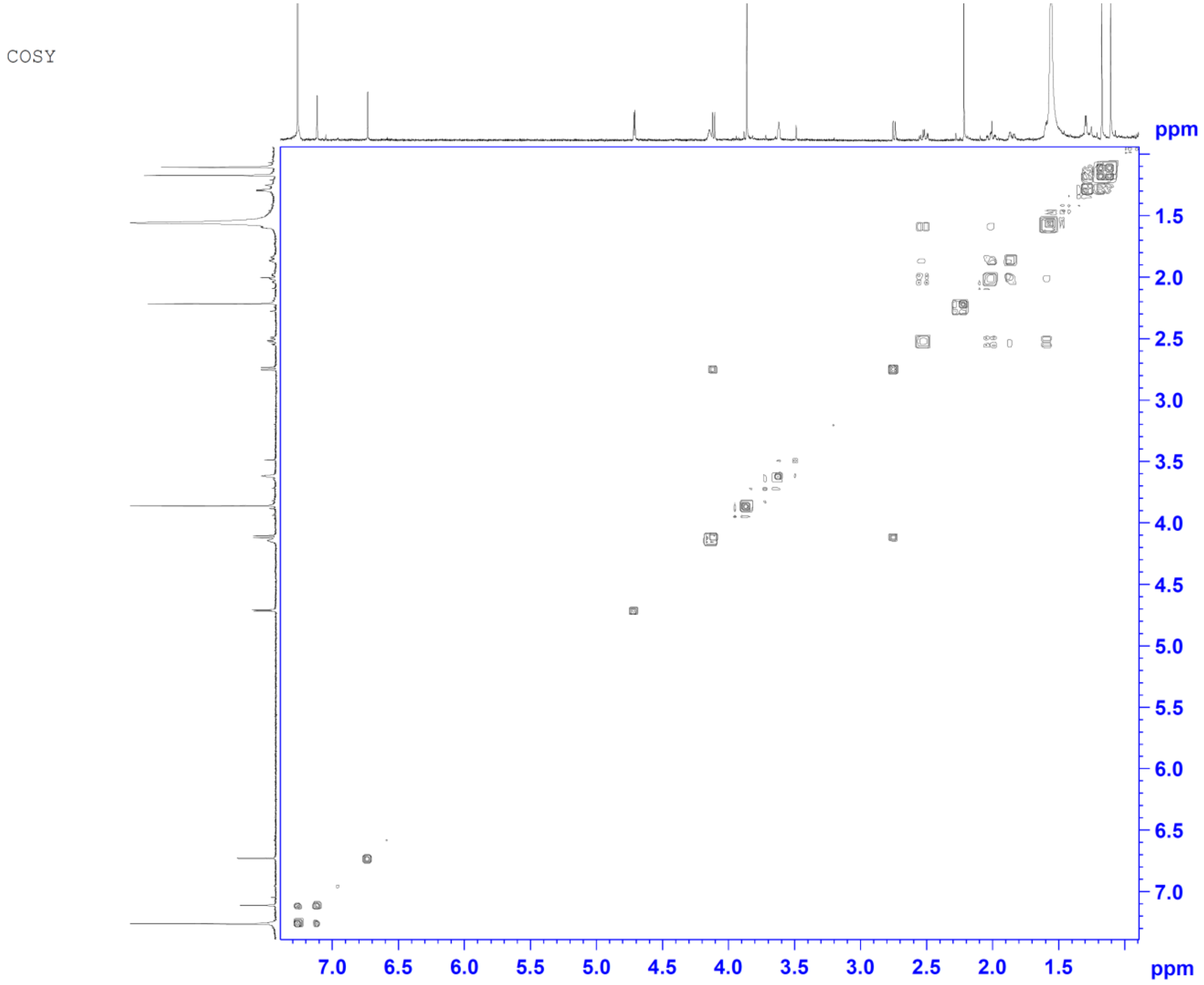

Figure S22. COSY spectrum of **3**.

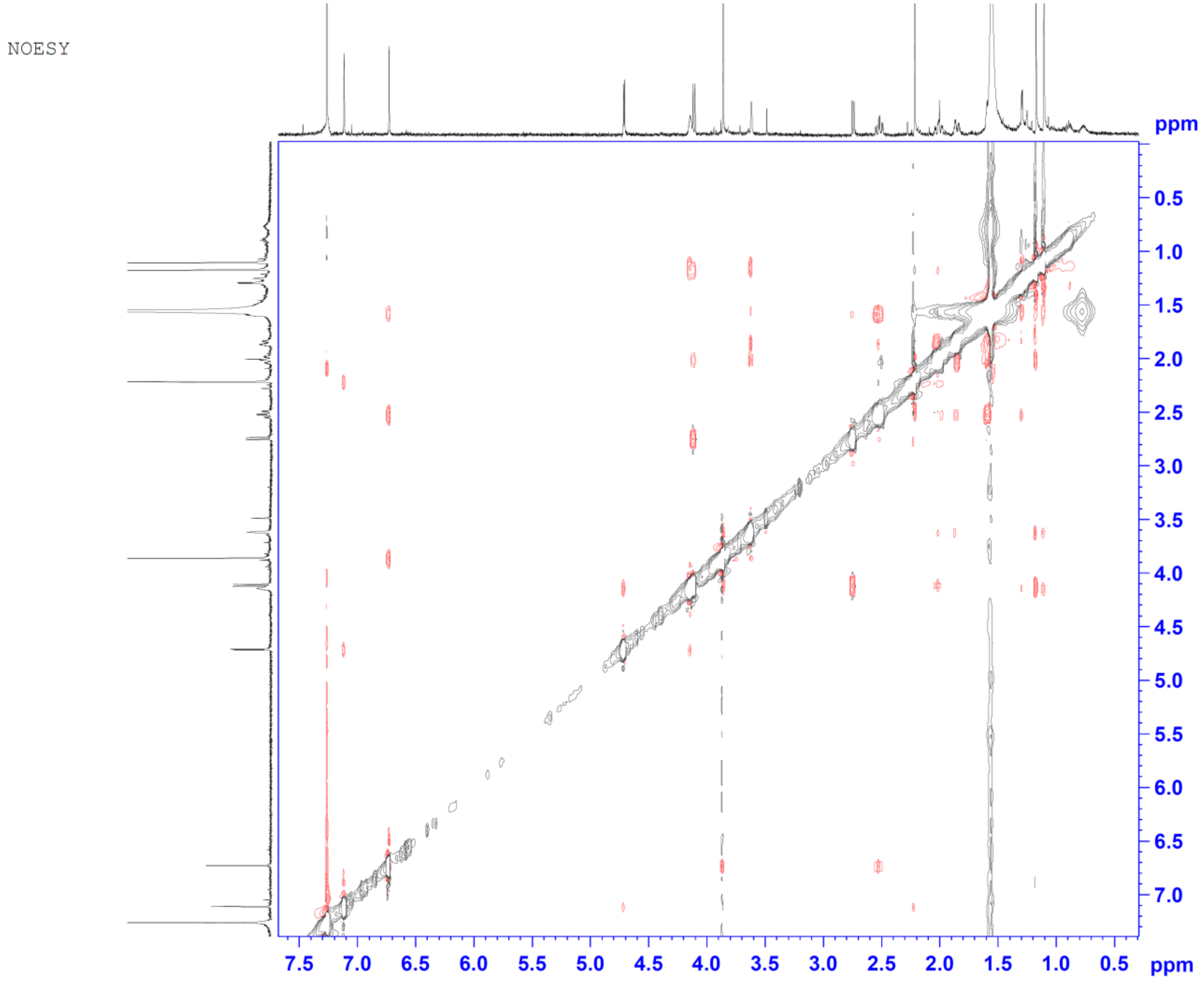

Figure S23. NOESY spectrum of **3**.

F:\Exp_data\...\80-FV-Cl-17K1L-H 2017/9/19 下午 03:51:33

80-FV-Cl-17K1L-H#1-20 RT: 0.02-0.54 AV: 20
T: FTMS - p ESI Full ms [300.00-350.00]
m/z= 335.1955-335.2456

Isotope	Min	Max
O-16	0	4
C-12	0	20
H-1	0	33

Charge 1
Mass tolerance 1000.00 ppm
Nitrogen rule not used
RDB equiv -1.00-100.00
max results 1

m/z	Intensity	Relative	Theo. Mass	Delta (ppm)	Composition
335.2221	5521533.5	100.00	335.2217	1.27	$C_{20}H_{31}O_4$

Figure S24. (–)-HR-ESIMS spectrum of **4**.

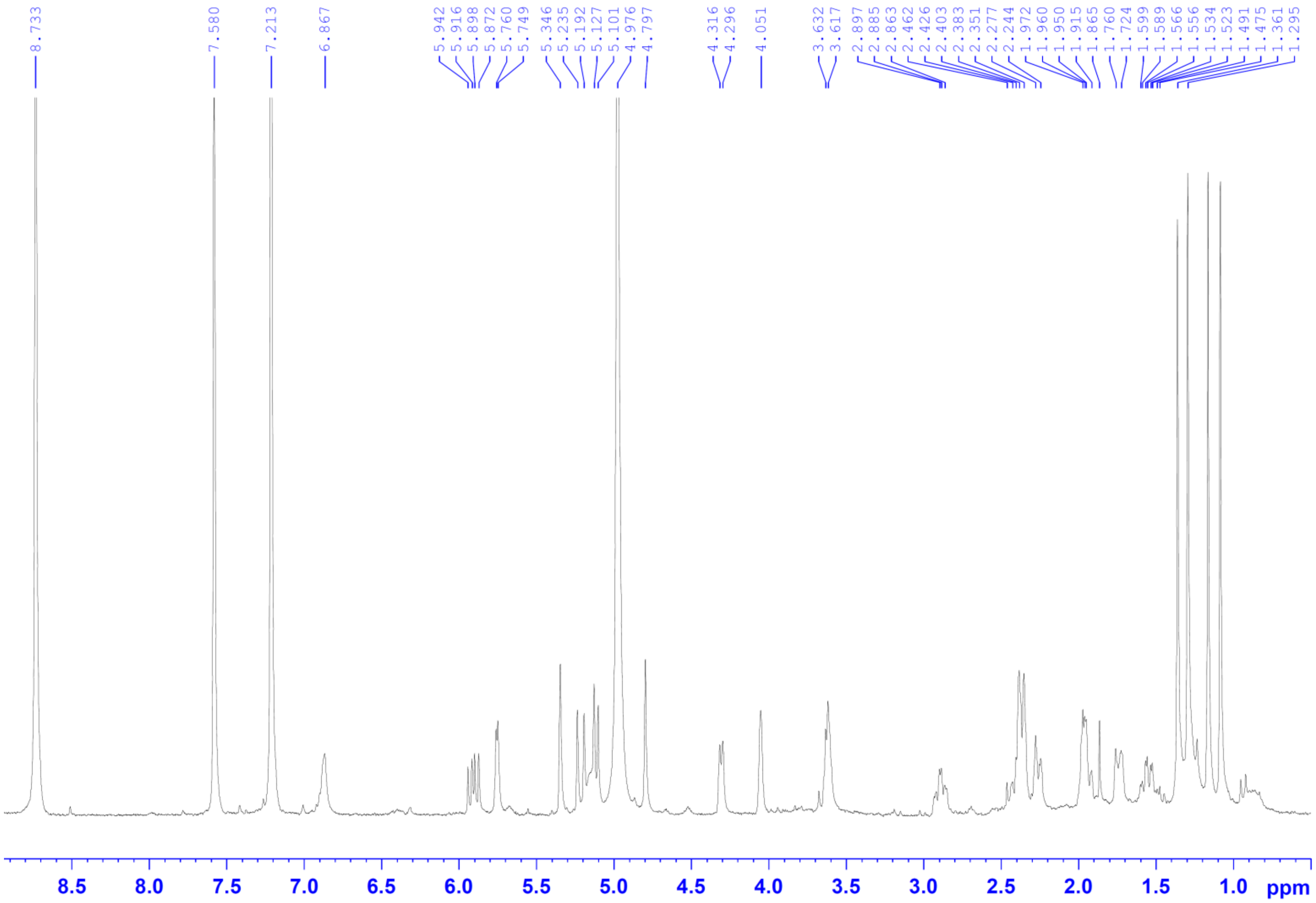

Figure S25. ^{1}H NMR spectrum of **4**.

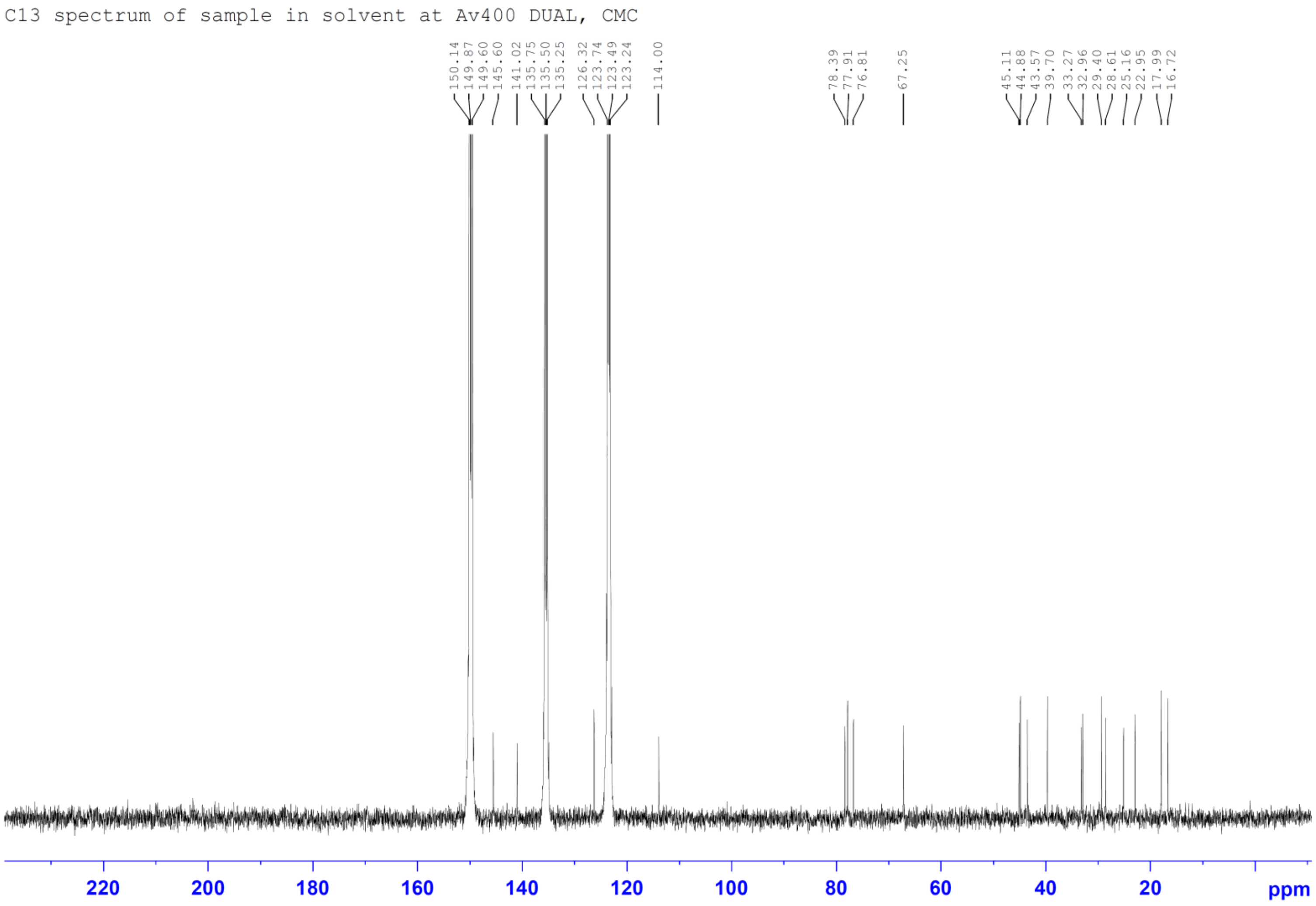

Figure S26. ^{13}C NMR spectrum of **4**.

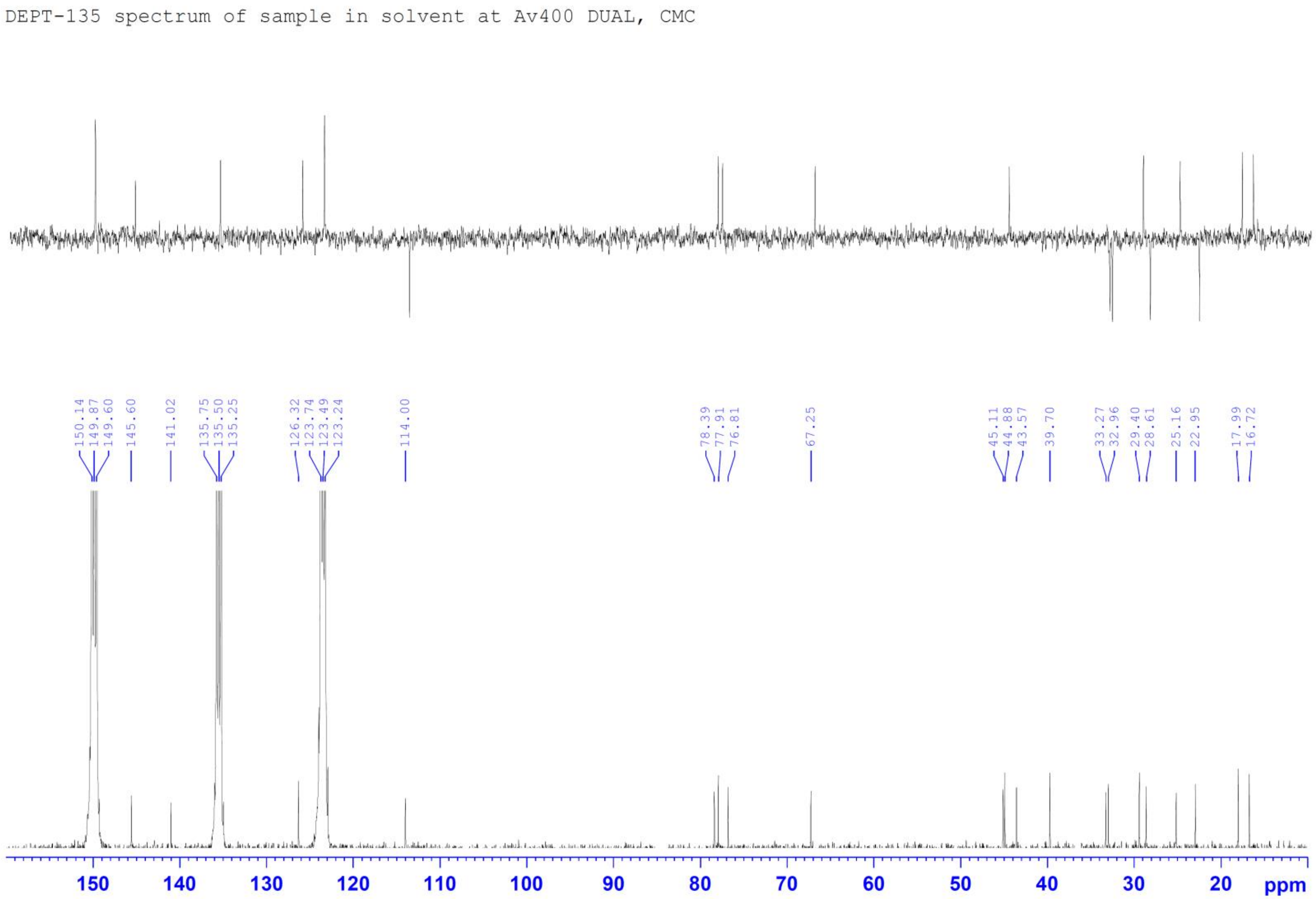

Figure S27. DEPT spectrum of **4**.

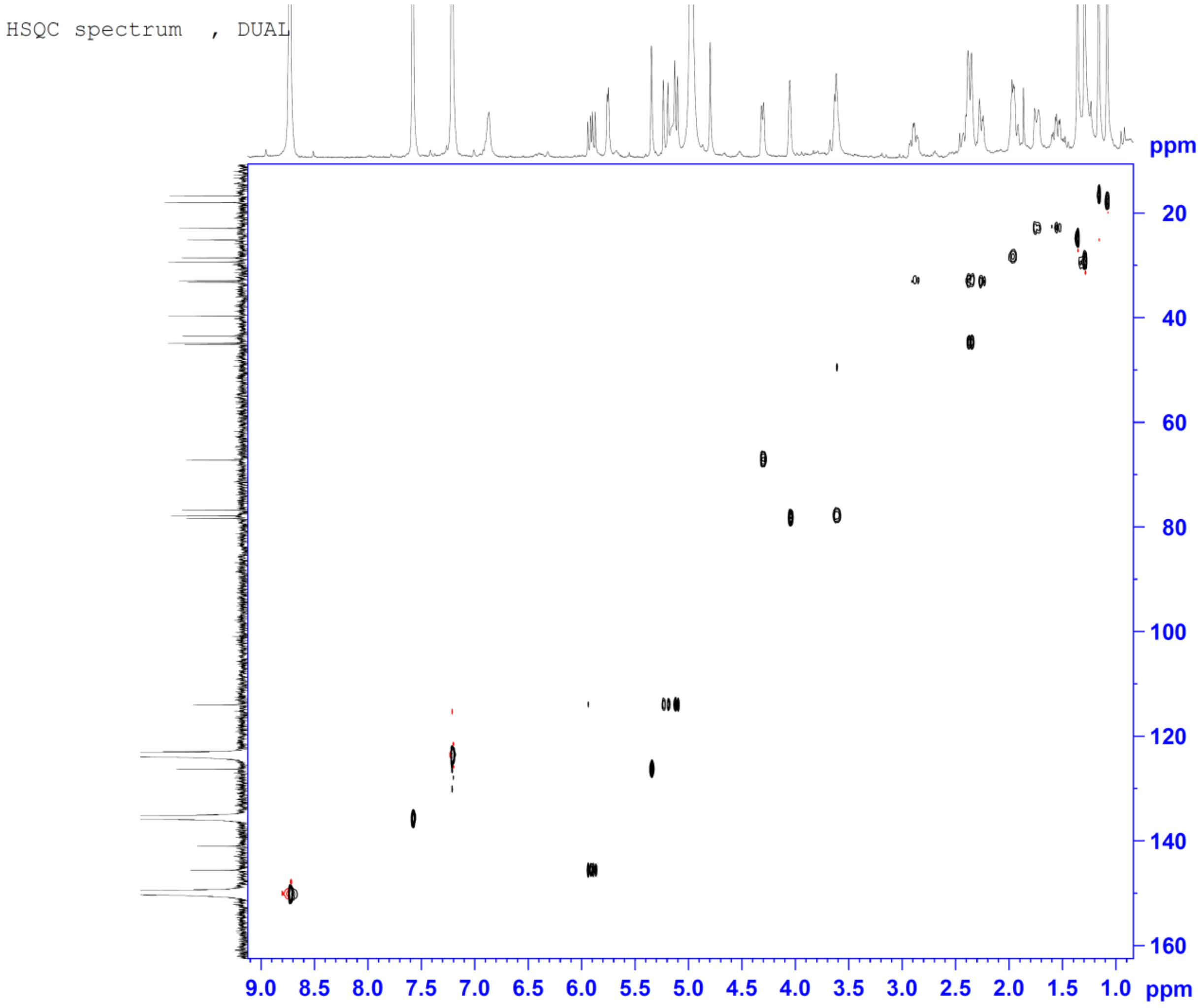

Figure S28. HSQC spectrum of **4**.

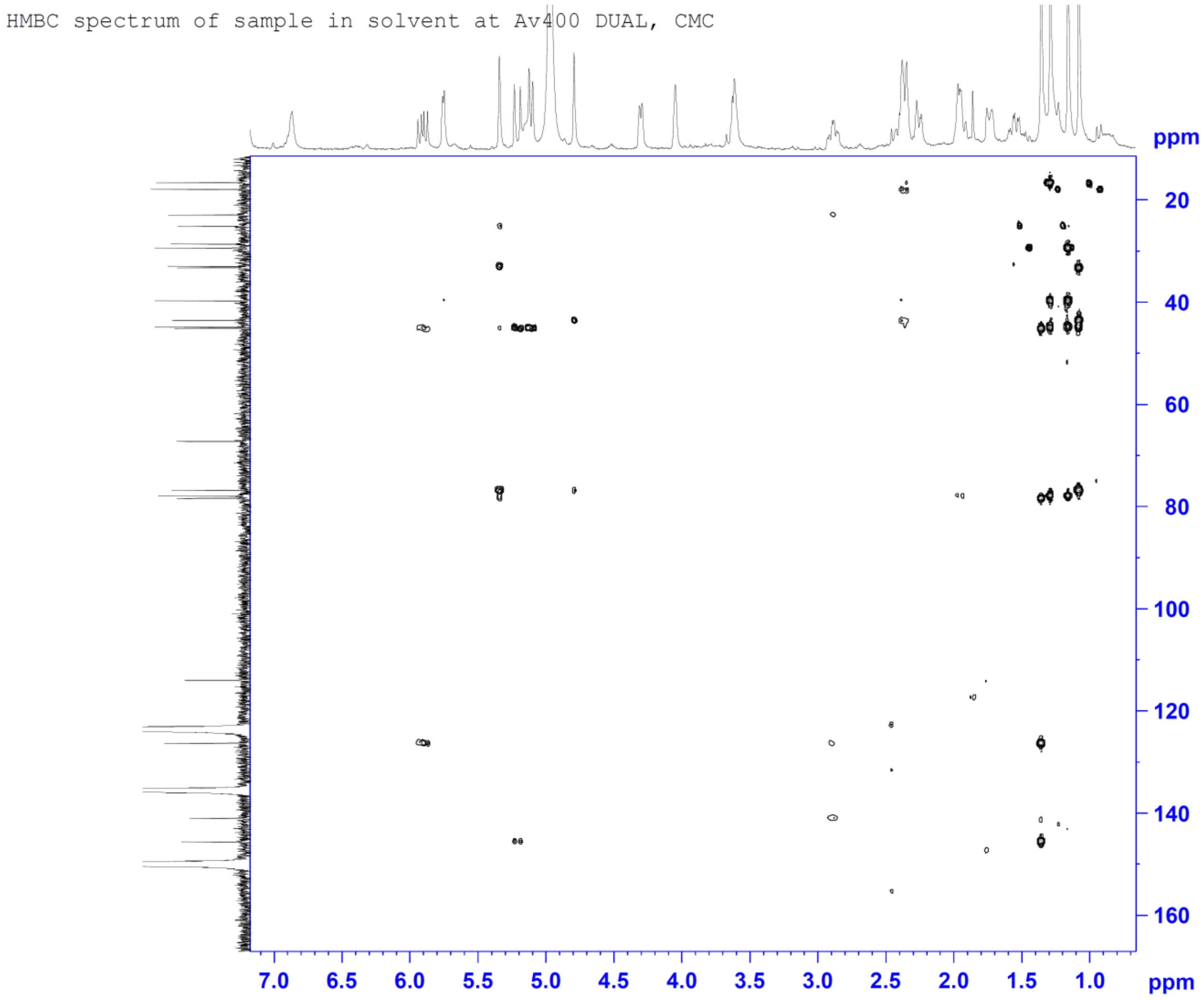

Figure S29. HMBC spectrum of **4**.

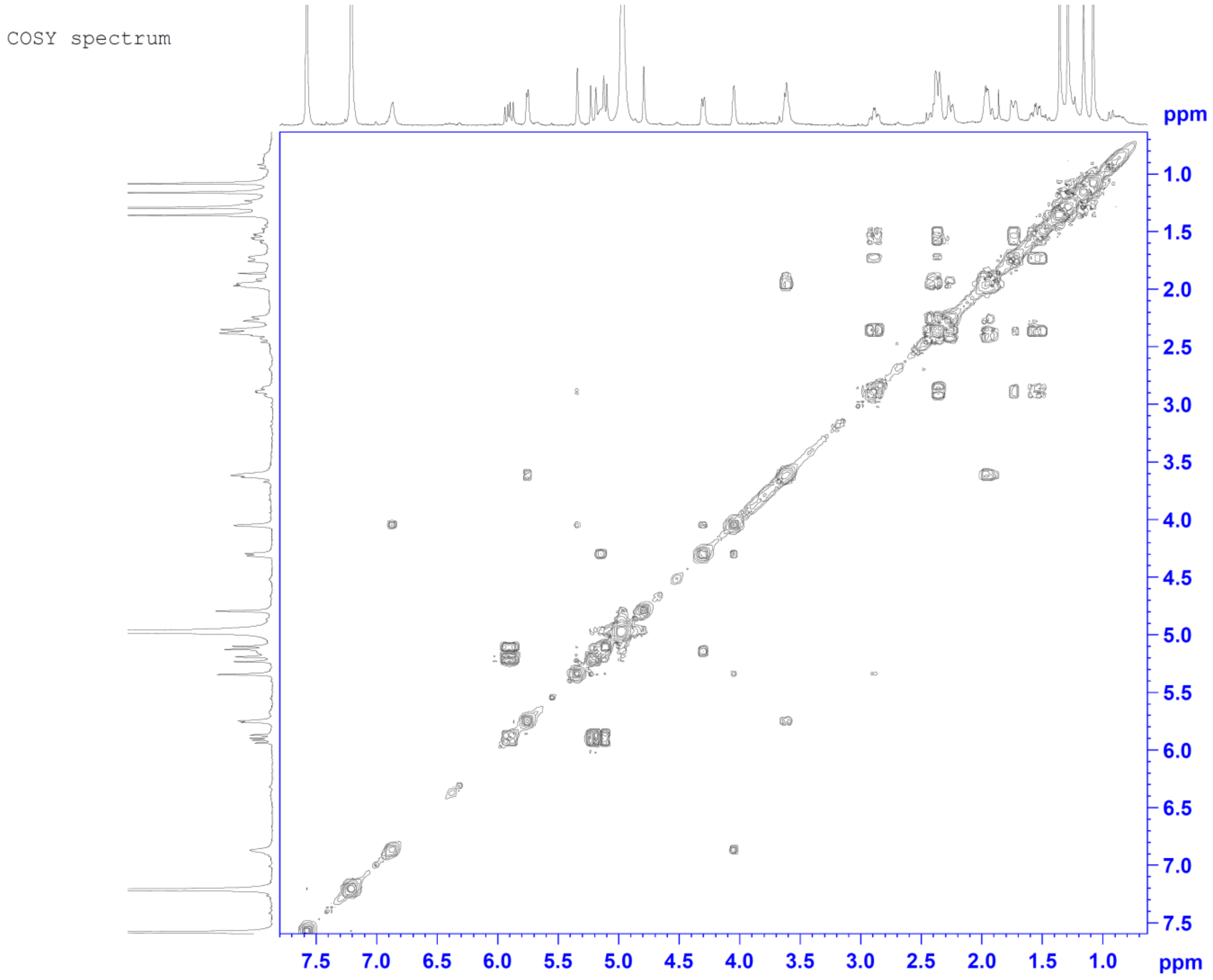

Figure S30. COSY spectrum of **4**.

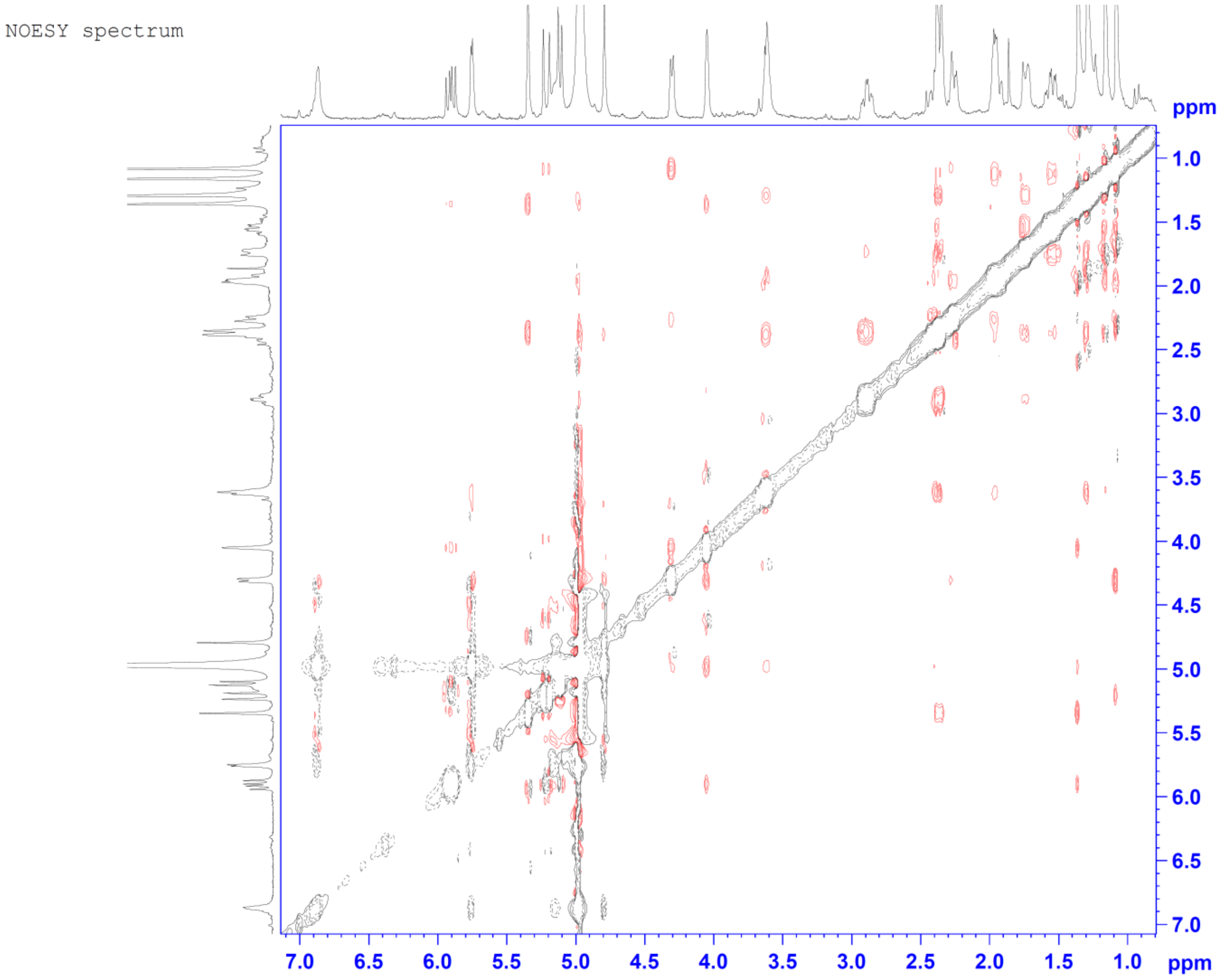

Figure S31. NOESY spectrum of **4**.

F:\Exp_data\...\30-FV-Cl-17J3C-APCI-H 2017/9/12 上午 11:28:26

30-FV-Cl-17J3C-APCI-H#1-20 RT: 0.00-0.52 AV: 20
T: FTMS - p APCI corona Full ms [300.00-340.00]
m/z= 317.1727-317.1781

Isotope	Min	Max
O-16	0	4
C-12	0	19
H-1	0	27

Charge 1
Mass tolerance 1000.00 ppm
Nitrogen rule not used
RDB equiv -1.00-100.00
max results 1

m/z	Intensity	Relative	Theo. Mass	Delta (ppm)	Composition
317.1752	329827.7	100.00	317.1747	1.46	$C_{19}H_{25}O_4$

Figure S32. (–)-HR-APCIMS spectrum of **5**.

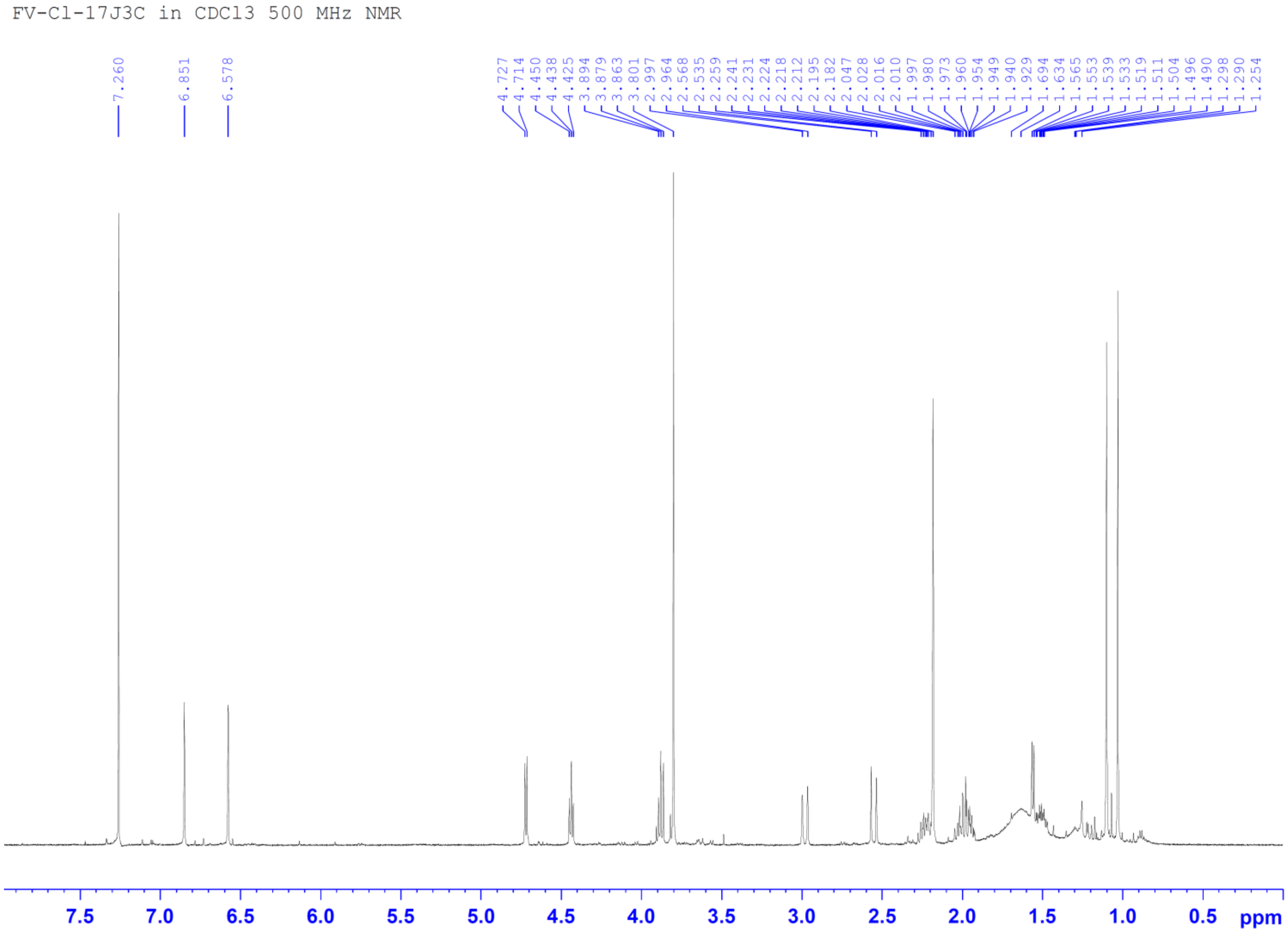

Figure S33. ^{1}H NMR spectrum of **5**.

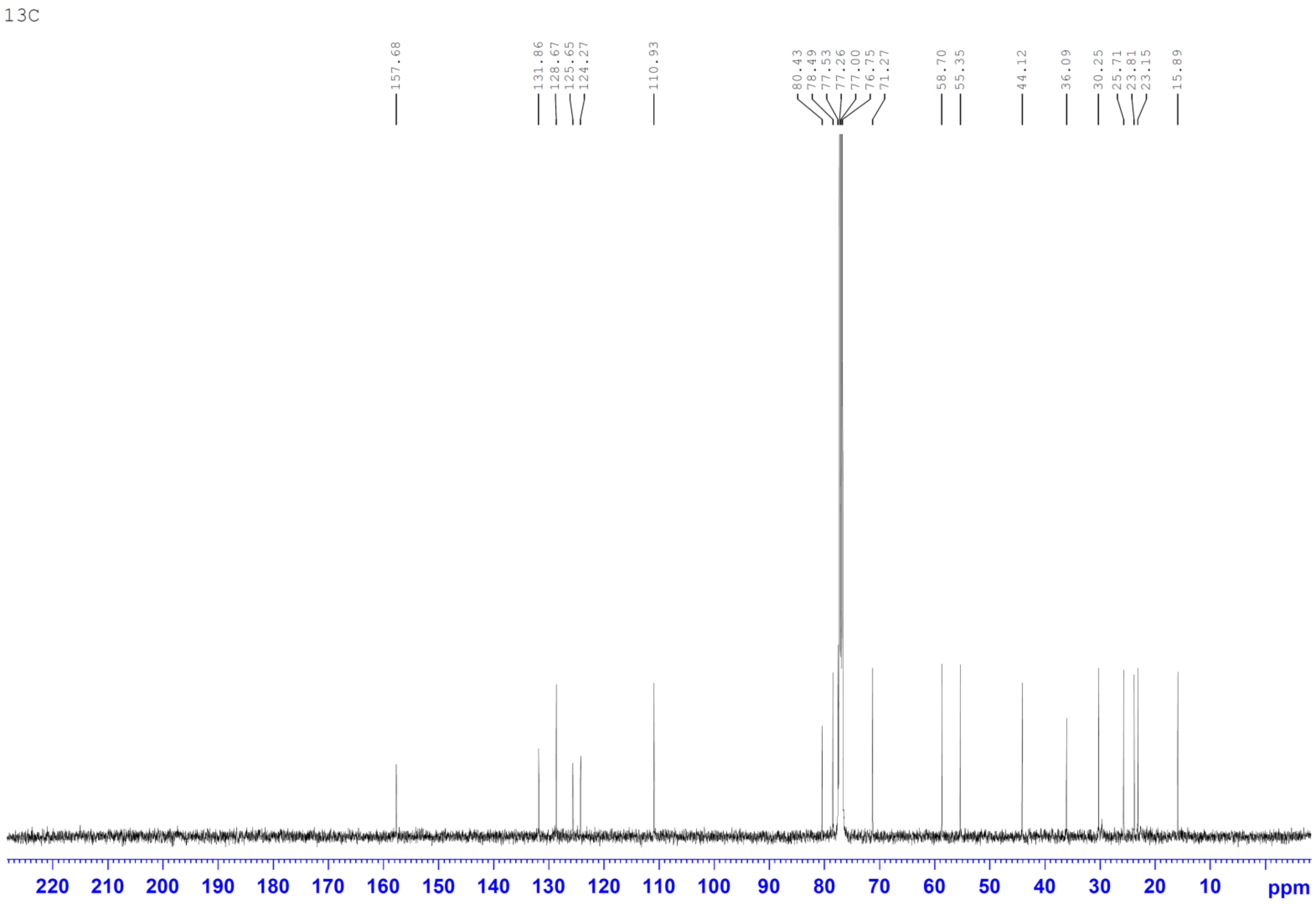

Figure S34. ^{13}C NMR spectrum of **5**.

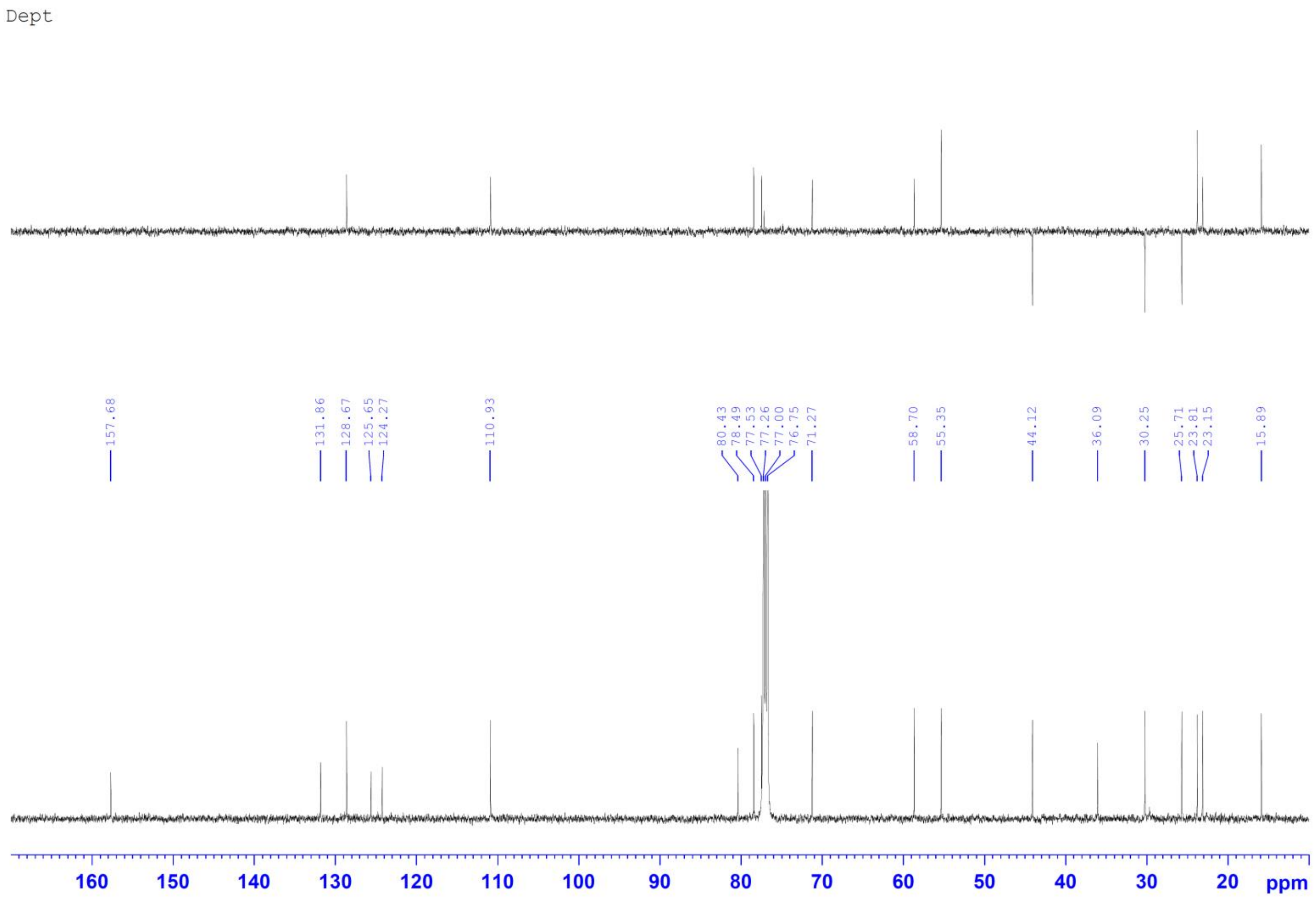

Figure S35. DEPT spectrum of **5**.

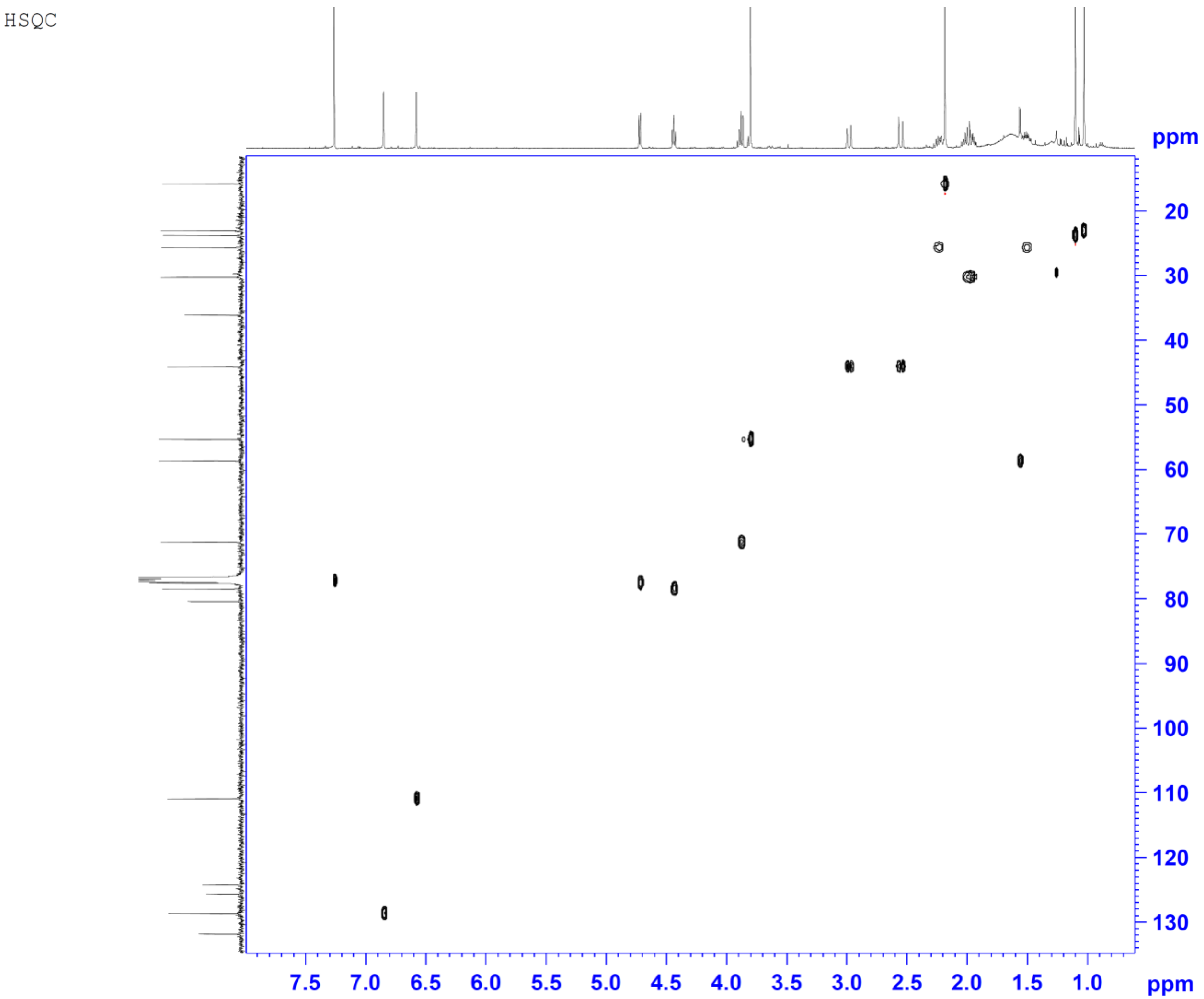

Figure S36. HSQC spectrum of **5**.

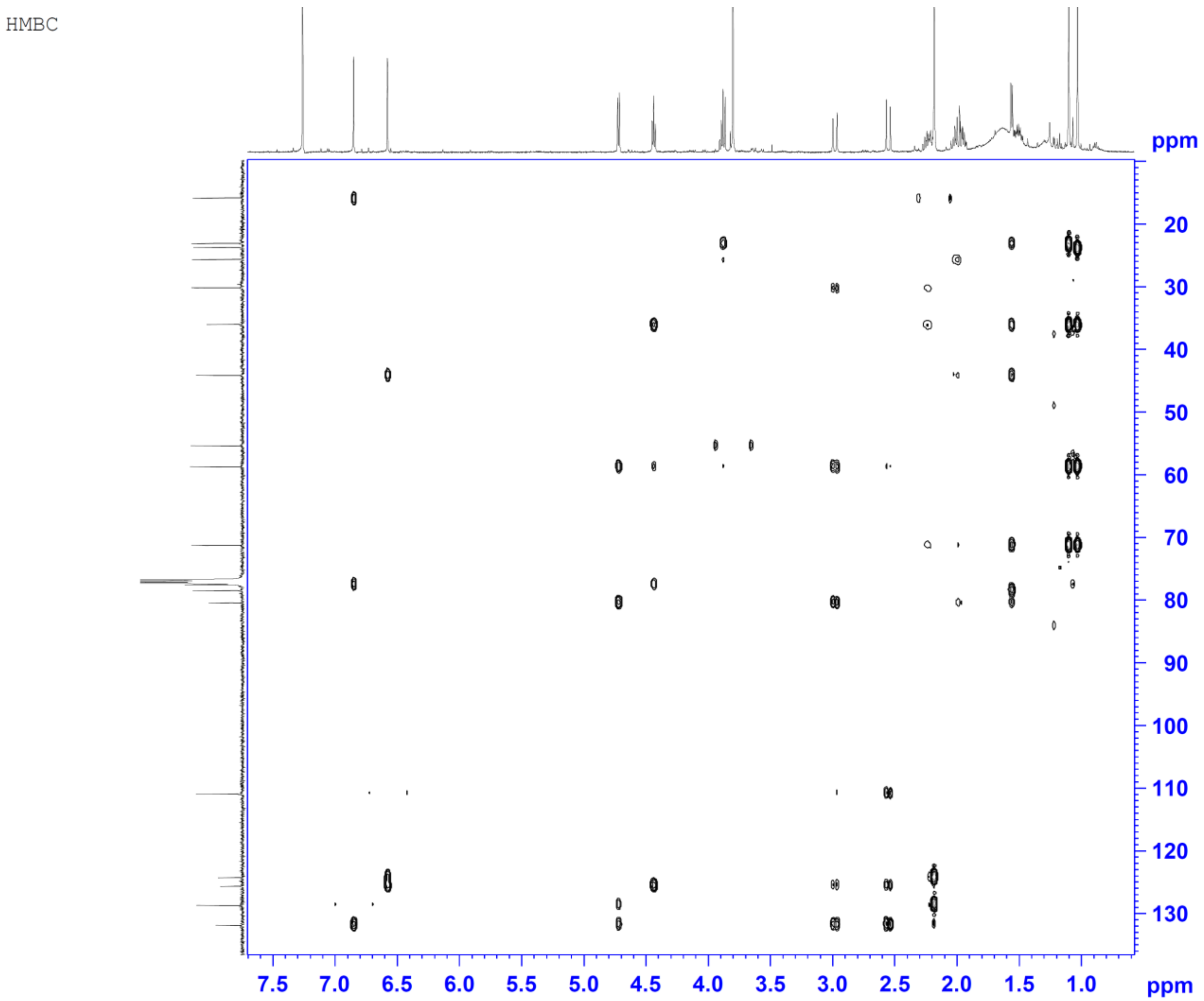

Figure S37. HMBC spectrum of **5**.

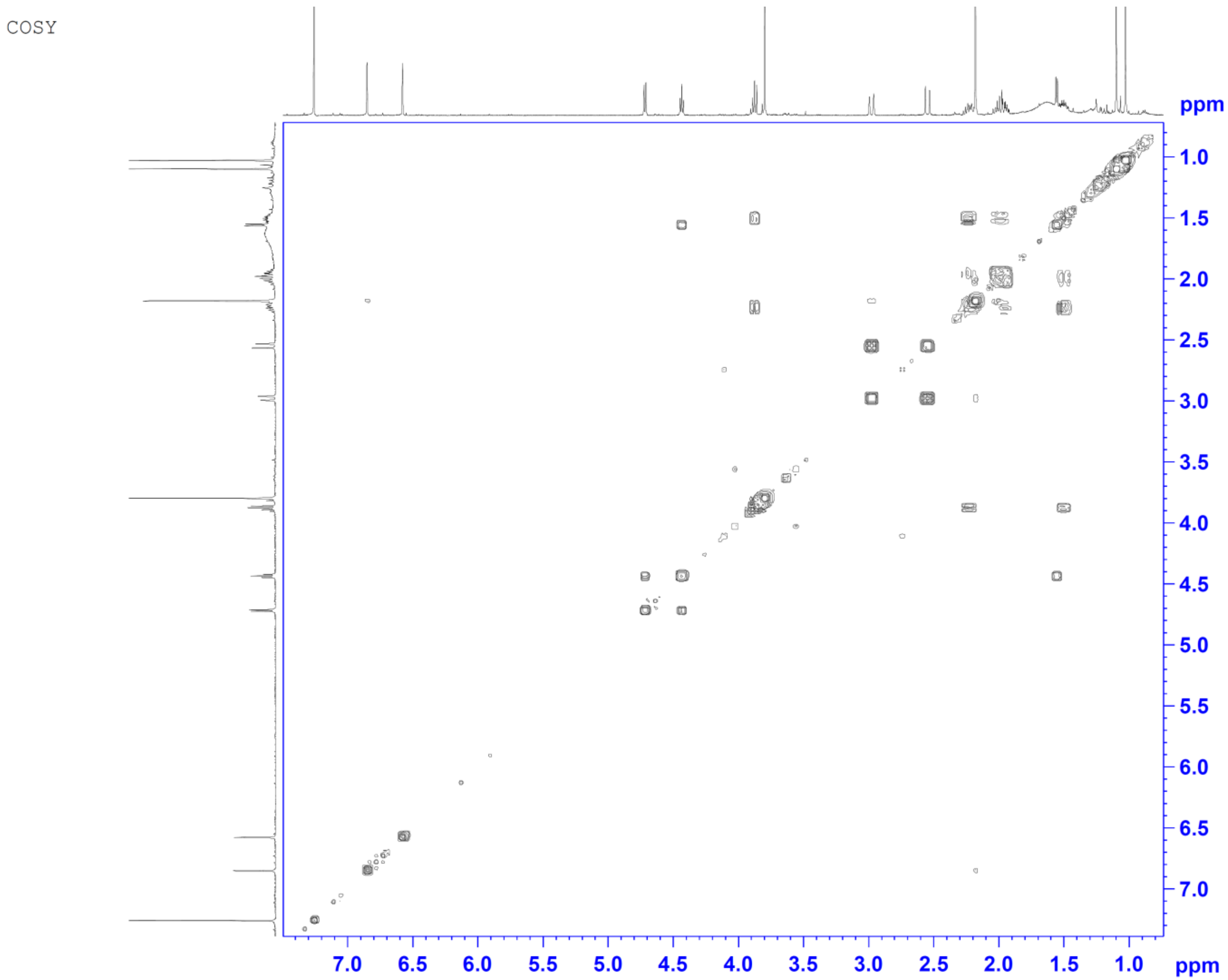

Figure S38. COSY spectrum of **5**.

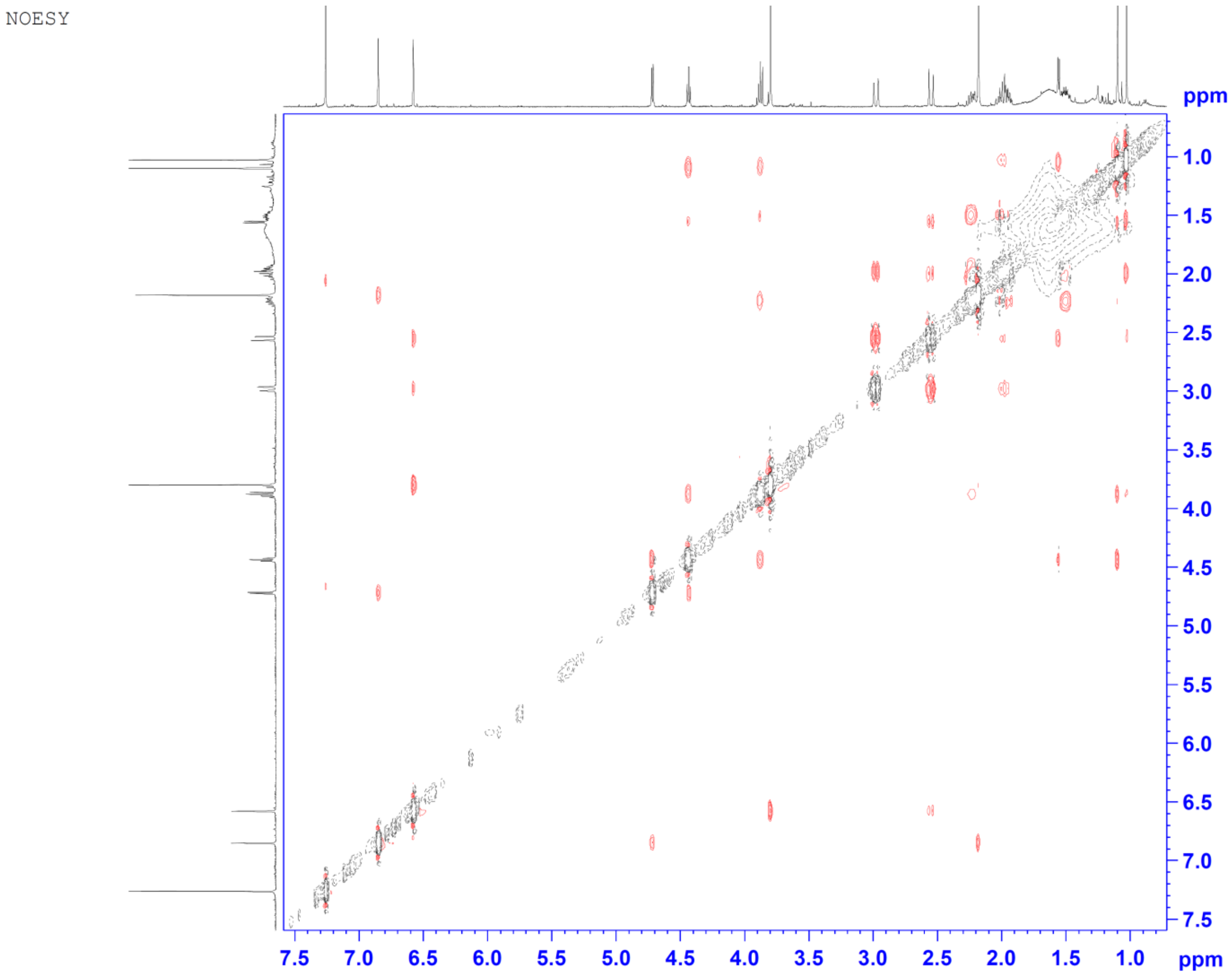

Figure S39. NOESY spectrum of **5**.

F:\Exp_data\...\07-FV-Cl-17J6C-H 2017/9/14 下午 02:11:36

07-FV-Cl-17J6C-H#1-20 RT: 0.01-0.28 AV: 20
T: FTMS + p ESI Full ms [150.00-500.00]
m/z= 341.1300-341.1982

Isotope	Min	Max
O-16	0	4
C-12	0	19
H-1	0	27
Na-23	0	1

Charge 1
Mass tolerance 1000.00 ppm
Nitrogen rule not used
RDB equiv -1.00-100.00
max results 1

m/z	Intensity	Relative	Theo. Mass	Delta (ppm)	Composition
341.1720	2807975.5	100.00	341.1723	-0.83	$C_{19}H_{26}O_4Na$

Figure S40. (+)-HR-ESIMS spectra of **6**.

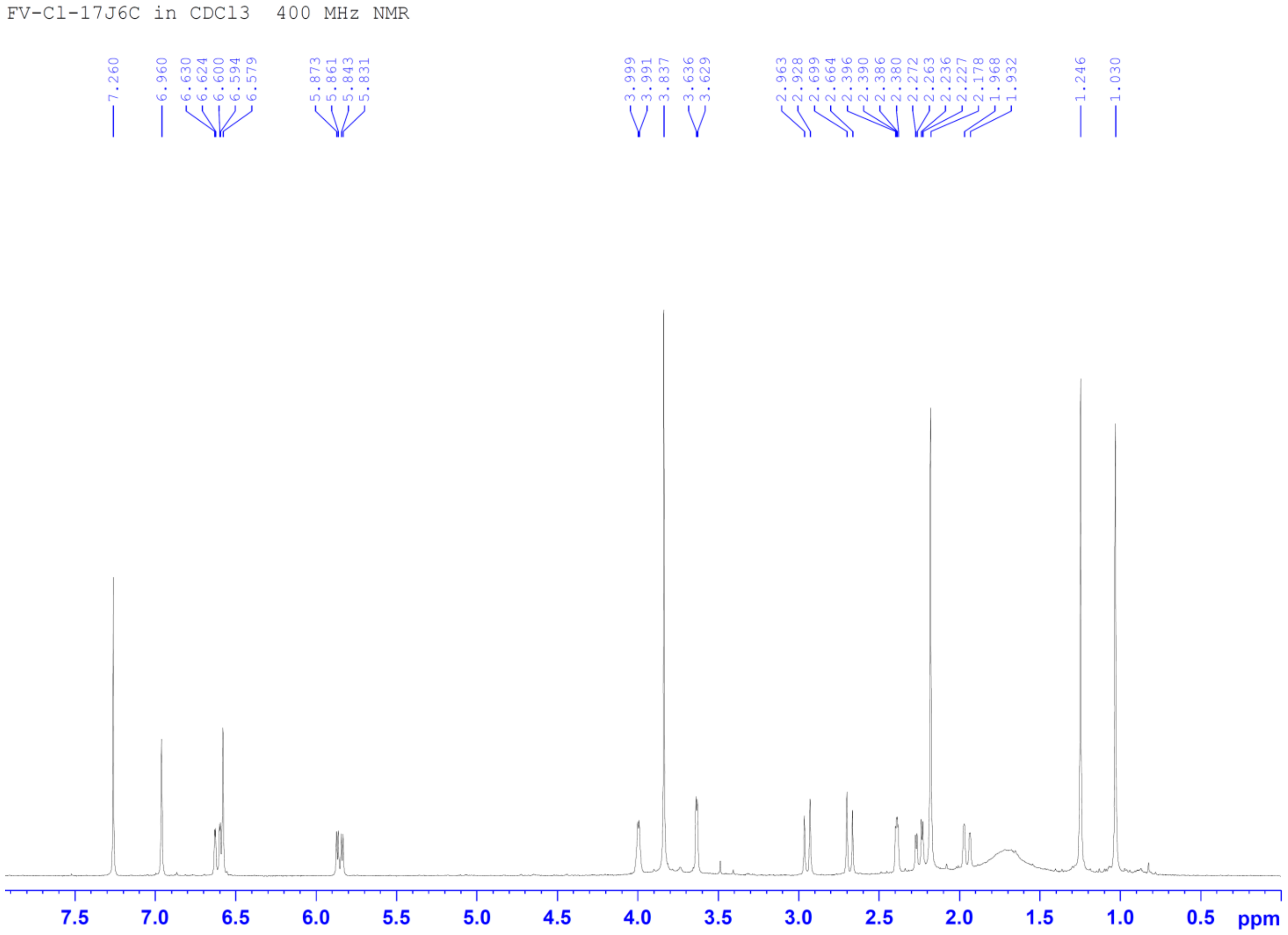

Figure S41. ^{1}H NMR spectrum of **6**.

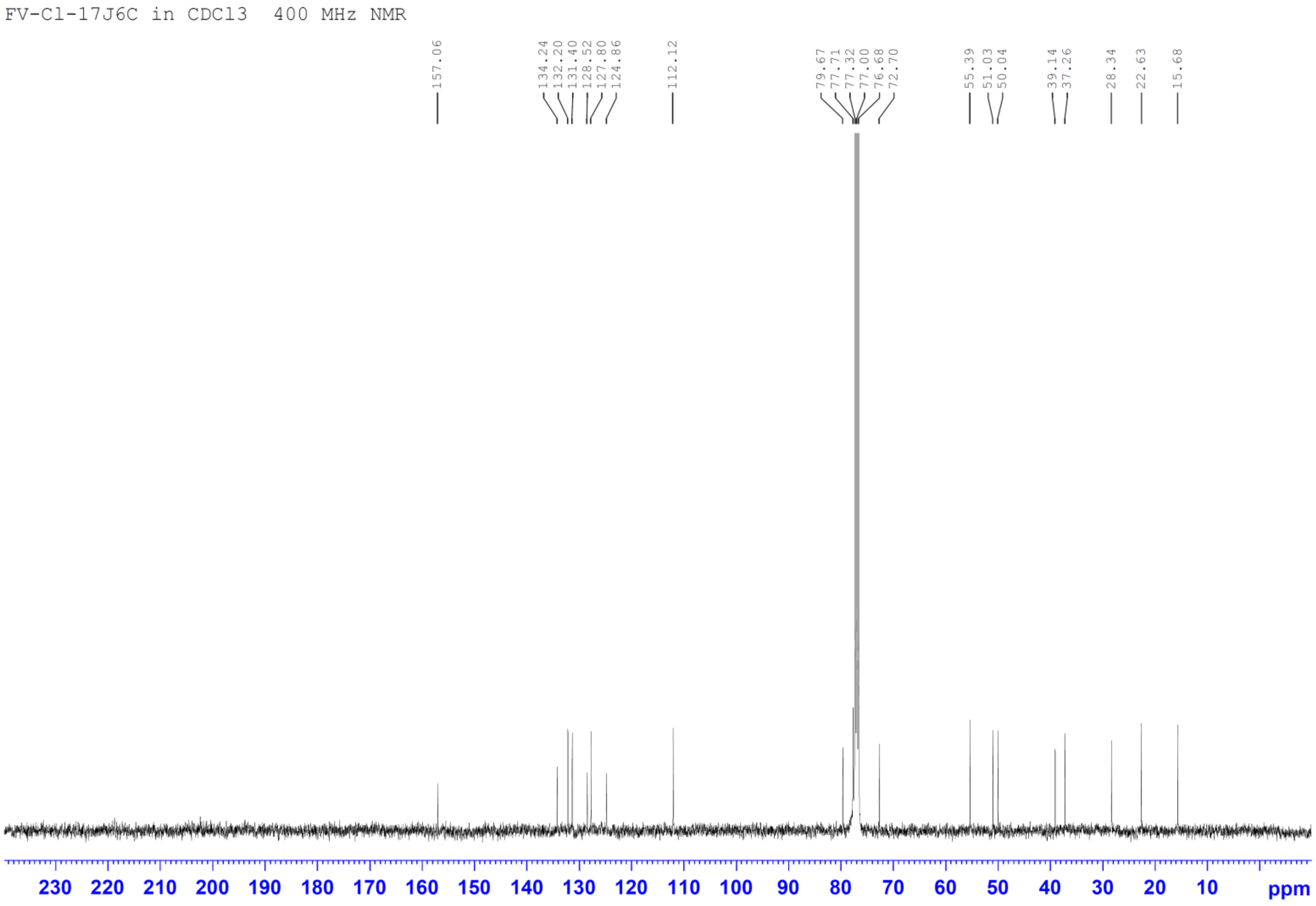

Figure S42. ^{13}C NMR spectrum of **6**.

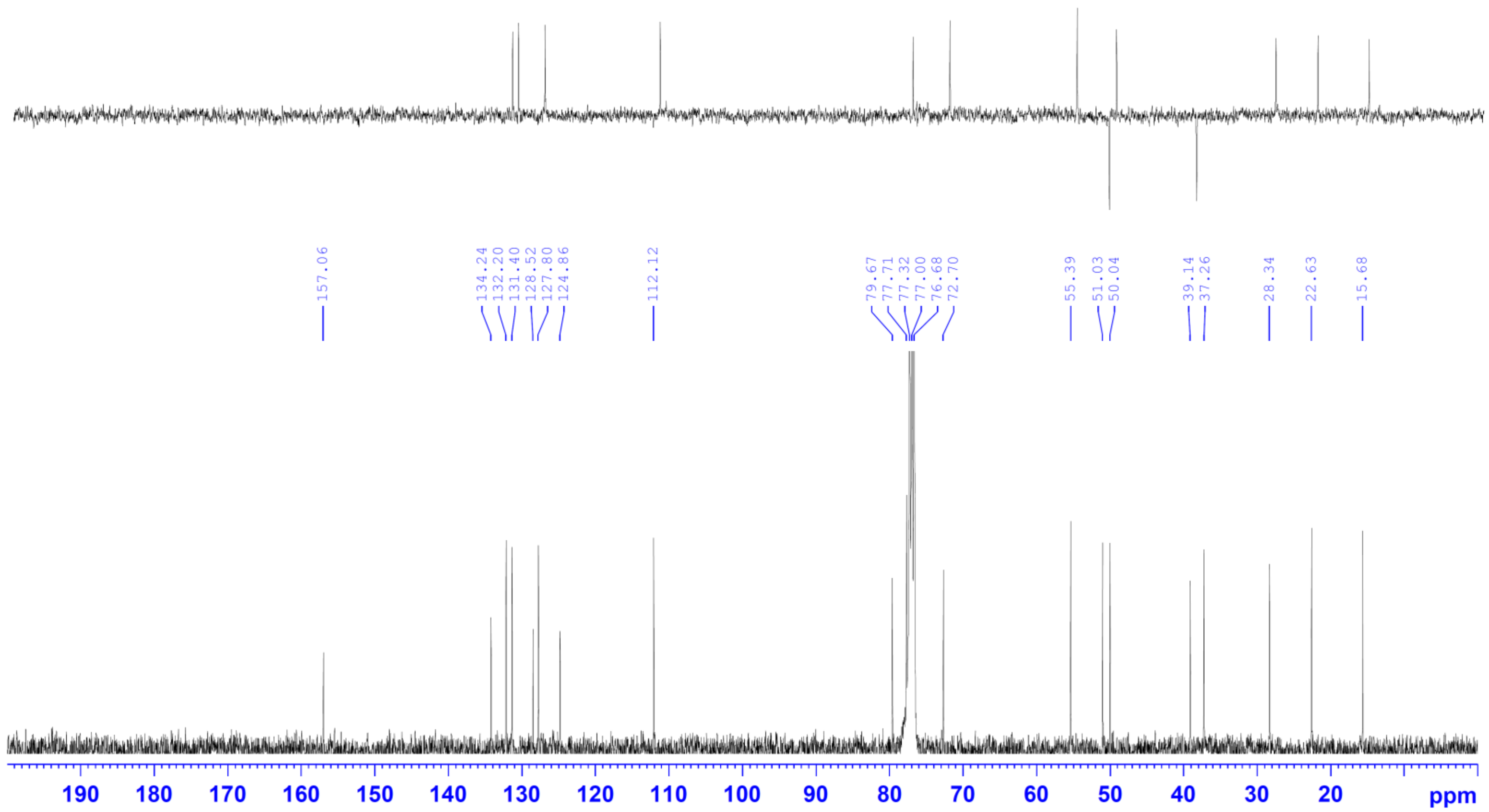

Figure S43. DEPT spectrum of **6**.

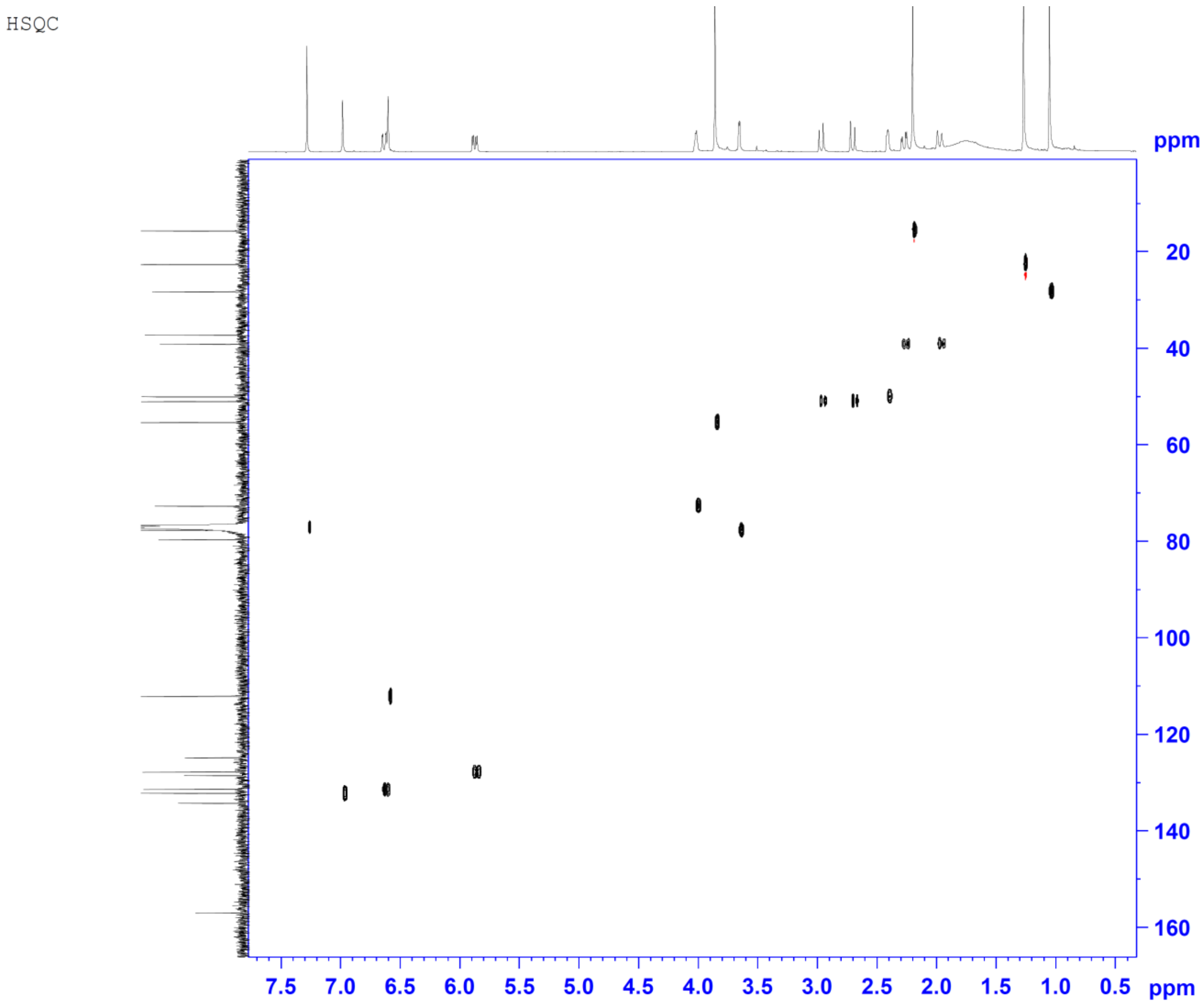

Figure S44. HSQC spectrum of **6**.

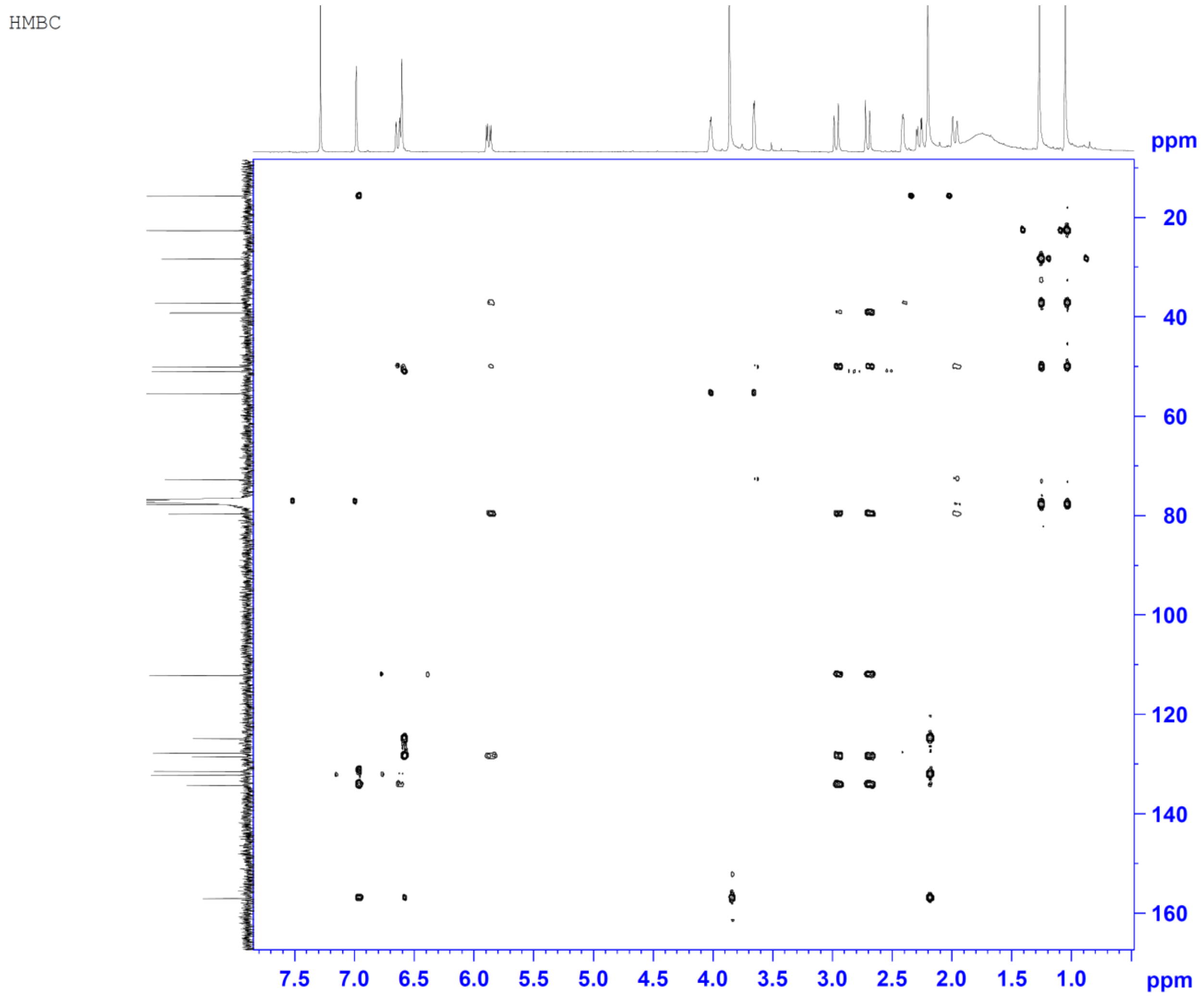

Figure S45. HMBC spectrum of **6**.

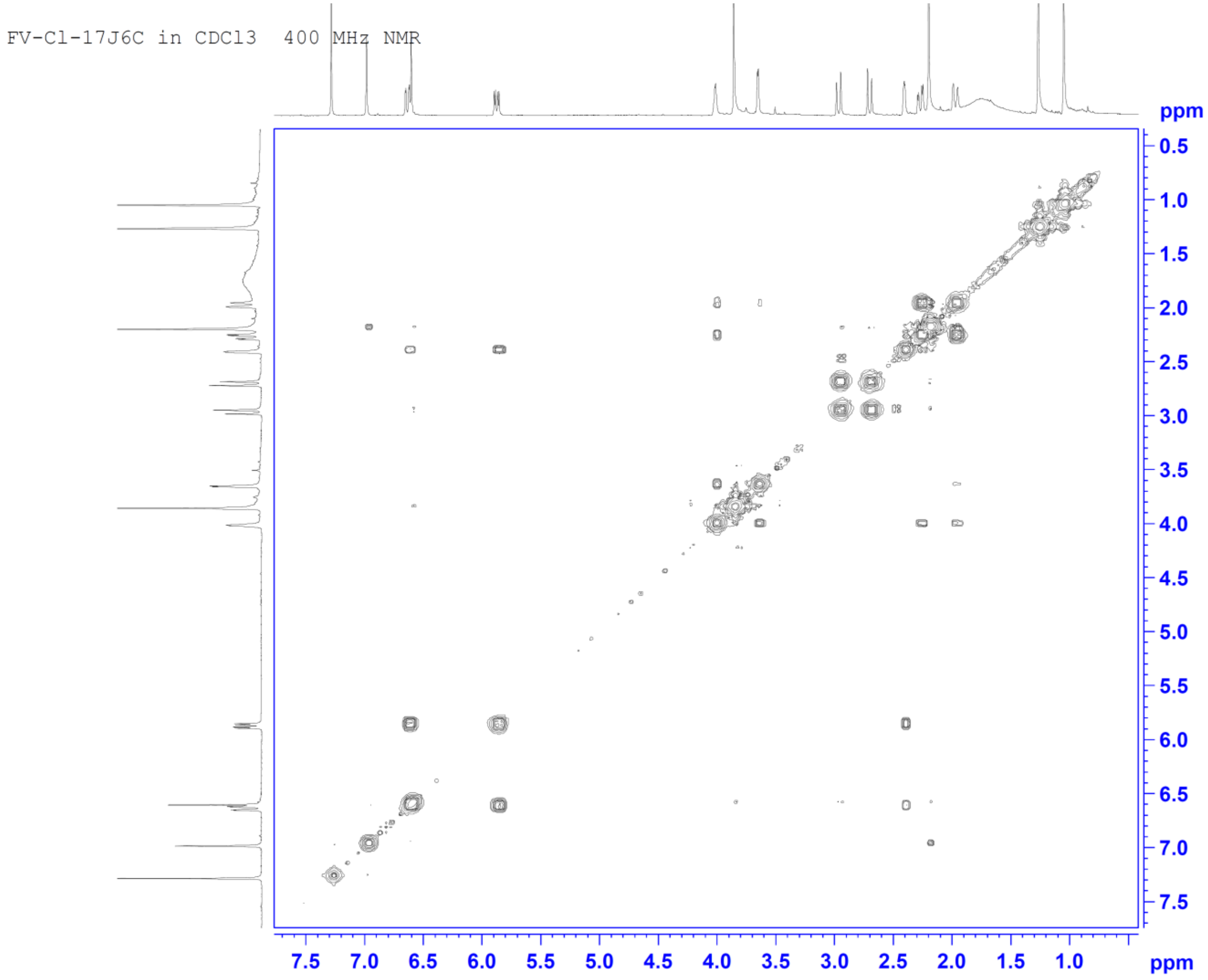

Figure S46. COSY spectrum of **6**.

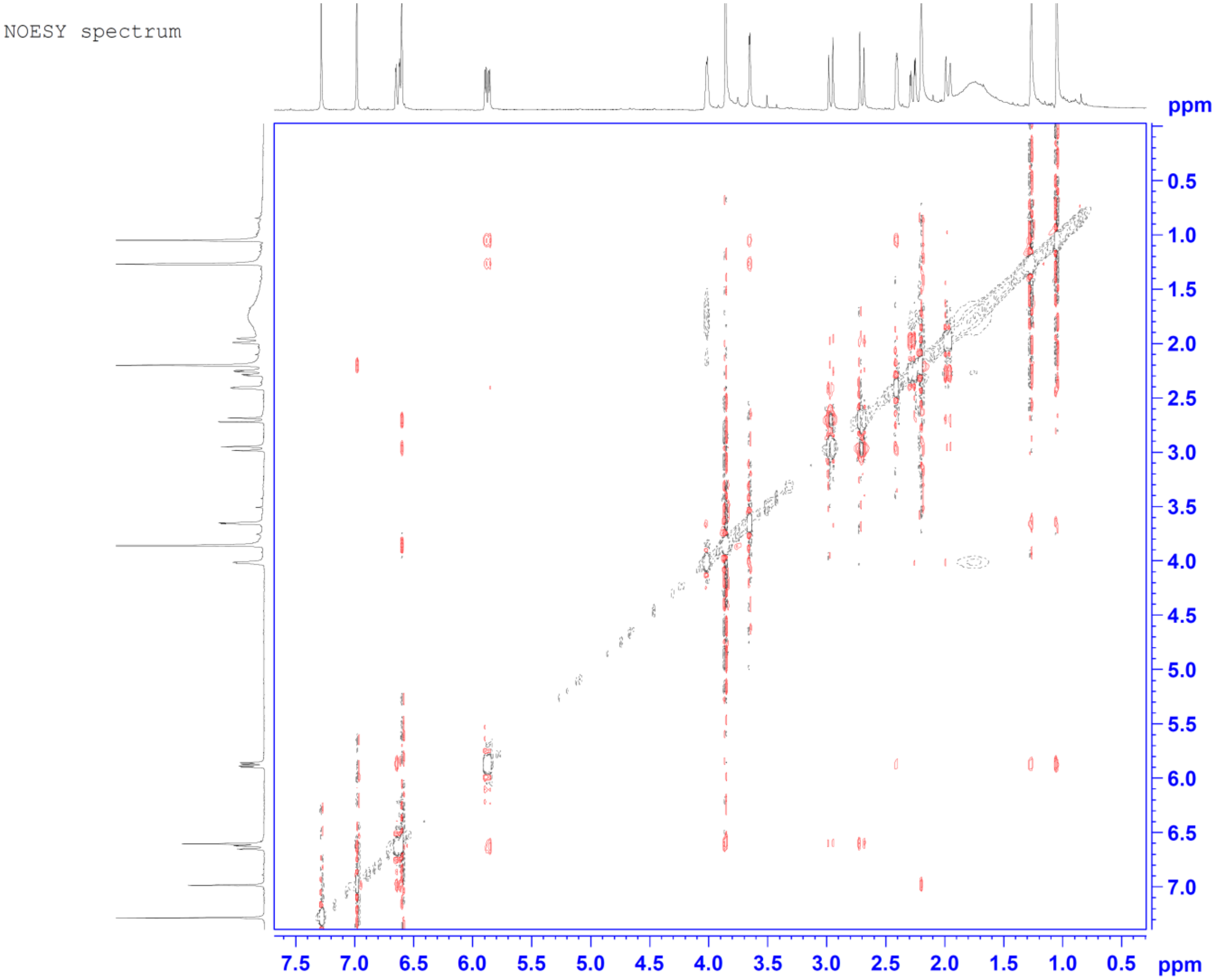

Figure S47. NOESY spectrum of **6**.

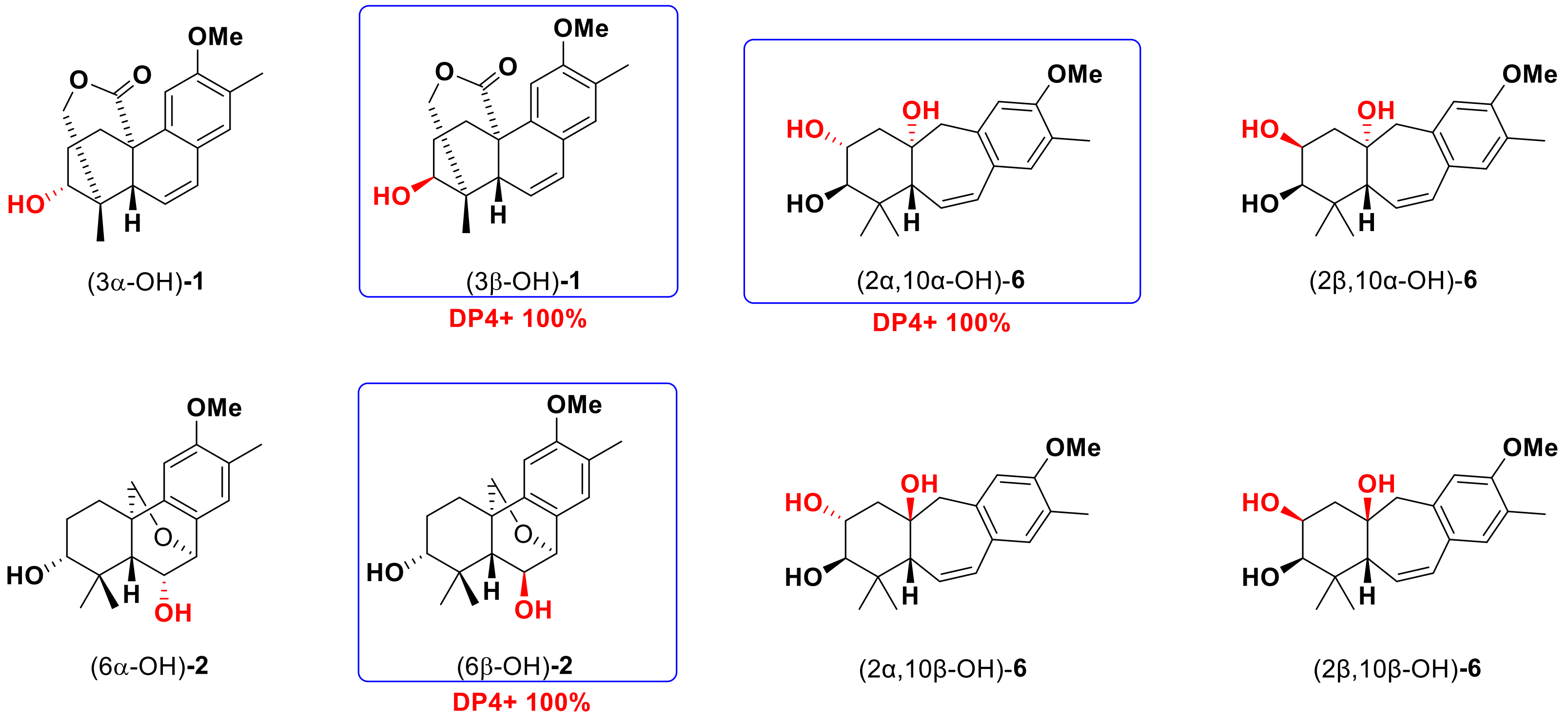

Figure S48. Possible candidates of compounds **1**, **2**, and **6** for DP4+ probability analysis.

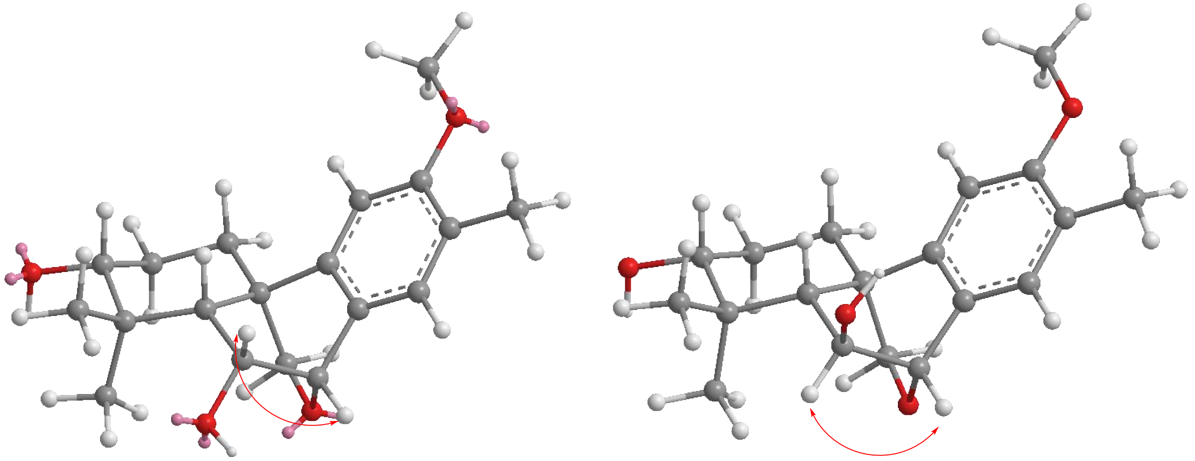

2.525 **Å** 2.476 **Å**

6α-OH 6β-OH

Figure S49. H-6/H-7 distance of 6α-OH and 6β-OH possible candidates of compound **2.** Conformers were generated by MM2 energy minimizations.

Table S1. DP4+ analysis table for compound **1** (isomer **1**: 3β-OH; isomer **2**: 3α-OH).

Functional	Solvent?	Basis Set
mPW1PW91	PCM	6-31+G(d,p)

Isomer Nº			1	2	3
DP4+ (%)		H data	100.00%	0.00%	-
		C data	100.00%	0.00%	-
		All data	100.00%	0.00%	-
Type	sp2?	Exp	1	2	3
C	x	108.1	89.59574001	89.2967897	
C	x	134.8	61.9701478	62.489968	
C	x	125.2	72.23589959	72.5038408	
C	x	129	69.60684022	69.4925838	
C	x	125.9	71.8181605	71.6585755	
C	x	157.4	41.8256481	41.7256232	
C		47.5	143.8645873	144.333204	
C		39	153.4398502	147.823136	
C	x	122.8	73.78434471	74.8083597	
C	x	131.2	66.20999243	66.4814807	
C		15.8	177.347593	177.423368	
C		27.9	163.9664729	160.691371	
C		28.9	164.7070977	163.300495	
C		74.5	119.3974051	120.03775	
C		36.6	155.7825877	155.226002	
C		19.3	176.4108609	176.814747	
C	x	174.5	24.27630265	24.4480983	
C		75.4	121.5269572	124.880552	
C		55.4	142.015428	142.109266	
H	x	6.93	24.51803458	24.5233053	
H	x	6.89	24.39986759	24.42251	
H		2.94	28.42534812	29.0671563	
H	x	5.9	25.34491231	25.2815031	
H	x	6.6	24.54488507	24.6163656	
H		2.18	29.33899984	29.3323841	
H		2.42	29.32183365	29.0578319	
H		2.28	29.33693171	29.660755	
H		2.1	29.66138839	29.5900136	
H		2.05	29.47941688	29.7443486	
H		3.81	27.72538307	27.8833957	
H		1.19	30.47799693	30.3829288	
H		4.04	27.52986444	26.8409481	
H		4.37	27.11379826	27.3127916	
H		3.88	27.62024425	27.6388816	

Table S2. Conformers and Boltzmann populations of 3α-OH isomer of **1**.

**3α1** (69.69%)				**3α2** (30.31%)			
C	1.99573	-0.7934	-0.17558	C	1.996059	-0.78716	-0.18317
C	0.972396	0.154266	-0.09847	C	0.972865	0.160853	-0.10569
C	1.314187	1.519463	-0.05034	C	1.315497	1.525083	-0.04084
C	2.667677	1.889923	-0.08531	C	2.669846	1.894231	-0.0618
C	3.697269	0.960313	-0.17587	C	3.69915	0.964633	-0.15348
C	3.337629	-0.40112	-0.21977	C	3.338642	-0.39609	-0.21317
C	-0.50657	-0.23837	0.025388	C	-0.50676	-0.23444	0.003024
C	-1.42088	0.831768	-0.63714	C	-1.42406	0.848296	-0.63578
C	-1.0211	2.229962	-0.21788	C	-1.01765	2.243124	-0.21326
C	0.261177	2.527135	0.035394	C	0.264722	2.535009	0.045192
C	5.147789	1.366235	-0.21767	C	5.150315	1.369428	-0.18045
C	-0.81086	-1.61655	-0.62888	C	-0.81206	-1.59208	-0.69457
C	-2.28814	-2.0092	-0.50429	C	-2.28579	-1.99869	-0.57227
C	-3.2263	-0.89451	-0.97142	C	-3.24888	-0.87673	-0.96675
C	-2.90694	0.491353	-0.35109	C	-2.90762	0.509558	-0.33479
O	-4.58612	-1.20387	-0.66161	O	-4.60244	-1.26046	-0.7234
C	-3.87369	1.515003	-0.9813	C	-3.88071	1.539038	-0.94214
C	-0.83933	-0.41502	1.529567	C	-0.83989	-0.46484	1.500359
O	-0.04477	-0.86407	2.320824	O	-0.05291	-0.95579	2.272872
O	-2.11163	-0.19585	1.943429	O	-2.1139	-0.25306	1.926113
C	-3.10804	0.507705	1.16877	C	-3.07906	0.522849	1.185637
O	4.38523	-1.27694	-0.30092	O	4.385806	-1.27191	-0.29407
C	4.11272	-2.66862	-0.28801	C	4.112096	-2.66366	-0.30147
H	1.753506	-1.8468	-0.18163	H	1.752946	-1.84018	-0.19978
H	2.920438	2.947843	-0.04472	H	2.923283	2.951438	-0.00925
H	-1.26557	0.755542	-1.72889	H	-1.28388	0.779997	-1.73017
H	-1.77464	3.010331	-0.19007	H	-1.76876	3.026315	-0.18988
H	0.554095	3.546309	0.280149	H	0.560452	3.552757	0.292573
H	5.249113	2.454567	-0.16581	H	5.252283	2.457208	-0.1193
H	5.708023	0.927018	0.616215	H	5.703421	0.923052	0.654381
H	5.634288	1.017913	-1.13669	H	5.643603	1.027651	-1.09826
H	-0.19982	-2.3958	-0.16531	H	-0.19372	-2.38481	-0.26511
H	-0.51791	-1.55806	-1.68543	H	-0.52986	-1.49527	-1.75092
H	-2.47525	-2.91475	-1.09911	H	-2.48791	-2.87497	-1.19907
H	-2.53162	-2.25966	0.534101	H	-2.4958	-2.30144	0.463059
H	-3.1148	-0.77738	-2.06427	H	-3.19515	-0.73582	-2.05473

H -4.76492 -2.10186 -0.98033	H -4.63327 -1.70469 0.138719
H -3.67739 1.611664 -2.05582	H -3.66687 1.683793 -2.00782
H -4.90696 1.181262 -0.85761	H -4.90772 1.174645 -0.85288
H -3.78222 2.508334 -0.53301	H -3.82198 2.516018 -0.45313
H -4.05924 0.04338 1.437047	H -4.05418 0.121034 1.478199
H -3.11053 1.542355 1.529789	H -3.01852 1.551593 1.558526
H 3.595822 -2.9698 0.632308	H 3.588017 -2.9763 0.610889
H 3.510865 -2.97114 -1.15559	H 3.516766 -2.95403 -1.17756
H 5.08464 -3.1636 -0.33568	H 5.084028 -3.15862 -0.34813

Table S3. Conformers and Boltzmann populations of 3β-OH isomer of **1**.

**3β1** (46.15%)	**3β2** (45.62%)	**3β3** (8.23%)
C 1.941671 -0.77581 -0.09939	C 1.943799 -0.77349 -0.10511	C 1.94132 -0.77485 -0.09879
C 0.898418 0.151465 -0.04939	C 0.900018 0.152633 -0.04962	C 0.89835 0.152885 -0.04836
C 1.206661 1.524348 -0.10651	C 1.207574 1.526065 -0.09838	C 1.206942 1.525684 -0.10544
C 2.548512 1.922298 -0.21176	C 2.549227 1.925754 -0.201	C 2.54882 1.923428 -0.21295
C 3.597651 1.01218 -0.27457	C 3.598835 1.016753 -0.26925	C 3.597501 1.013185 -0.27731
C 3.27111 -0.35682 -0.21643	C 3.272982 -0.35295 -0.21967	C 3.270869 -0.3561 -0.21737
C -0.56615 -0.26144 0.153777	C -0.56344 -0.26564 0.150659	C -0.56585 -0.26068 0.154867
C -1.52124 0.730938 -0.57301	C -1.52135 0.73383 -0.56113	C -1.52279 0.735734 -0.56441
C -1.14912 2.164769 -0.26346	C -1.14869 2.166619 -0.24786	C -1.1491 2.168692 -0.25207
C 0.130542 2.510609 -0.06223	C 0.131478 2.511696 -0.04819	C 0.131741 2.512103 -0.0546
C 5.035418 1.447565 -0.39323	C 5.036374 1.45346 -0.38529	C 5.035155 1.447937 -0.39805
C -0.85677 -1.70205 -0.35921	C -0.85498 -1.69824 -0.3852	C -0.85537 -1.70101 -0.35971
C -2.31906 -2.11517 -0.1477	C -2.3166 -2.11923 -0.18235	C -2.32071 -2.11916 -0.16265
C -3.29522 -1.07577 -0.70594	C -3.29736 -1.07618 -0.71279	C -3.29883 -1.0799 -0.71844
C -2.99023 0.369242 -0.22687	C -2.98649 0.368661 -0.21093	C -2.9918 0.369626 -0.2251
O -3.24113 -1.04877 -2.13684	O -3.22825 -1.15894 -2.13945	O -3.35788 -1.14251 -2.1468
C -4.01287 1.312666 -0.89185	C -4.00354 1.329829 -0.86012	C -4.01595 1.313017 -0.88415
C -0.84932 -0.30648 1.675857	C -0.84317 -0.3329 1.671945	C -0.85027 -0.31128 1.677631
O -0.02256 -0.64194 2.488654	O -0.01494 -0.67804 2.478795	O -0.02101 -0.65221 2.485601
O -2.1263 -0.10631 2.101864	O -2.12034 -0.14118 2.104835	O -2.1233 -0.10603 2.104801
C -3.15377 0.4991 1.292522	C -3.14767 0.479799 1.310766	C -3.15578 0.492817 1.295331
O 4.337394 -1.21223 -0.2766	O 4.339362 -1.20707 -0.28541	O 4.336336 -1.21098 -0.27778
C 4.099361 -2.60592 -0.17094	C 4.101731 -2.60218 -0.19172	C 4.100397 -2.60496 -0.16352
H 1.725293 -1.83264 -0.0298	H 1.727327 -1.83065 -0.04251	H 1.725633 -1.83167 -0.02674
H 2.776005 2.985946 -0.25233	H 2.776041 2.989812 -0.23509	H 2.776402 2.987016 -0.2532

H	-1.39384	0.572838	-1.65526	H	-1.40206	0.577778	-1.64553	H	-1.38347	0.593969	-1.65159
H	-1.92028	2.928655	-0.27703	H	-1.91988	2.931028	-0.25474	H	-1.92137	2.931052	-0.25664
H	0.403323	3.551522	0.100598	H	0.404354	3.551912	0.119175	H	0.405232	3.55189	0.11379
H	5.110735	2.538997	-0.41783	H	5.11131	2.545042	-0.40389	H	5.111052	2.539233	-0.4233
H	5.633844	1.079037	0.448357	H	5.634774	1.080499	0.454363	H	5.634121	1.079303	0.443031
H	5.50023	1.049293	-1.30315	H	5.501429	1.060208	-1.29726	H	5.498478	1.048698	-1.30826
H	-0.2094	-2.41889	0.153877	H	-0.20882	-2.42245	0.119207	H	-0.21292	-2.41867	0.157816
H	-0.60911	-1.73428	-1.42638	H	-0.6052	-1.71659	-1.4519	H	-0.57429	-1.74121	-1.42204
H	-2.50564	-3.08265	-0.63607	H	-2.50731	-3.06276	-0.70514	H	-2.50946	-3.07614	-0.66267
H	-2.52271	-2.27173	0.917524	H	-2.51749	-2.29457	0.879799	H	-2.51848	-2.27497	0.903345
H	-4.3198	-1.32696	-0.38199	H	-4.31606	-1.33571	-0.37775	H	-4.31774	-1.33225	-0.40038
H	-3.4011	-1.95113	-2.45354	H	-3.95498	-0.63657	-2.51024	H	-2.45047	-1.12911	-2.48862
H	-3.88984	1.306827	-1.97684	H	-3.82111	1.429784	-1.93576	H	-3.92503	1.273501	-1.97181
H	-5.03456	0.978916	-0.67144	H	-5.02896	0.964074	-0.71652	H	-5.03586	0.998459	-0.63009
H	-3.92175	2.341982	-0.53326	H	-3.96054	2.335429	-0.43274	H	-3.90306	2.350381	-0.55678
H	-4.08943	0.041442	1.632556	H	-4.08414	0.019279	1.645994	H	-4.08714	0.023131	1.630603
H	-3.18299	1.56054	1.563956	H	-3.17344	1.53699	1.600037	H	-3.19611	1.551683	1.575355
H	5.080792	-3.08142	-0.22325	H	5.083424	-3.07659	-0.24786	H	5.082531	-3.07893	-0.21359
H	3.621604	-2.86261	0.783502	H	3.62394	-2.86684	0.760435	H	3.623671	-2.85603	0.792783
H	3.475737	-2.97385	-0.99689	H	3.478359	-2.96287	-1.02086	H	3.47712	-2.97855	-0.98709

Table S4. DP4+ analysis table for compound **2** (isomer **1**: 6α-OH; isomer **2**: 6β-OH).

Functional	Solvent?	Basis Set
mPW1PW91	PCM	6-31+G(d,p)

Isomer Nº			1	2	3
DP4+ (%)		H data	0.00%	100.00%	-
		C data	0.00%	100.00%	-
		All data	0.00%	100.00%	-
Type	sp2?	Exp	1	2	3
C		27	166.9032247	166.529575	
C		78.5	116.7934818	116.806488	
C		39.1	151.6956414	152.89649	
c		56.8	148.2454013	137.615652	
c		39.2	153.5290984	152.479861	
c		25.9	167.5768392	167.609611	
c		68.9	122.6160673	125.022165	
c		72.9	118.1037707	120.823519	
c	x	125.9	70.7826572	72.0894995	
c	x	145.1	51.37148232	51.1426929	
c	x	129	71.24476556	69.4267738	
c	x	124.6	73.08509916	73.0708289	
c	x	158.3	40.89726929	40.6921424	
c	x	101.6	97.17987369	97.5057014	
c		15.9	177.3707034	177.330815	
c		65.8	127.5627827	129.543047	
c		28.7	169.6653998	167.531664	
c		15.4	179.5177543	179.92844	
c		55.5	142.1017936	142.192915	
H		0.91	30.43274742	30.818264	
H		1.88	29.95801379	29.8915114	
H		1.73	29.79565981	29.8576739	
H		3.4	28.22982641	28.148608	
H		2.16	29.36782698	29.4270667	
H		1.88	29.85722374	29.7747043	
H		4.21	27.6502101	27.4409883	
H		4.72	27.04143838	26.960035	
H	x	7.12	24.21051712	24.2096163	
H	x	6.67	24.76929582	24.7275913	
H		2.22	29.3598753	29.338014	
H		4.07	27.09451381	27.3561901	
H		2.72	28.73106972	28.8548811	
H		1.14	30.42756119	30.4905711	
H		1.14	30.1676364	30.4267473	
H		3.86	27.64320049	27.6226388	

Table S5. Conformers and Boltzmann populations of 6α-OH isomer of **2**.

**6α1** (52.98%)	**6α2** (26.20%)	**6α3** (20.82%)
C 2.237828 2.146824 0.890175	C 2.237042 2.140624 0.88706	C 2.22783 2.158625 0.871023
C 2.856339 1.689916 -0.42712	C 2.859168 1.681081 -0.43441	C 2.863252 1.691807 -0.44127
C 2.816024 0.136741 -0.58713	C 2.81804 0.136438 -0.598	C 2.818721 0.13874 -0.60201
C 1.301176 -0.27778 -0.52899	C 1.302602 -0.2805 -0.52861	C 1.304456 -0.28401 -0.52761
C 0.438756 0.315256 0.658973	C 0.439726 0.311833 0.659733	C 0.440835 0.308818 0.658602
C 0.746981 1.799189 0.919802	C 0.745373 1.796186 0.918351	C 0.74111 1.795464 0.911254
C 0.991022 -1.823 -0.66865	C 0.993017 -1.82643 -0.66589	C 0.993033 -1.83052 -0.66538
C -0.12728 -2.16752 0.333819	C -0.12959 -2.16869 0.333004	C -0.1341 -2.17064 0.32893
C -1.32062 -1.28824 0.132754	C -1.32166 -1.28842 0.131514	C -1.324 -1.28911 0.128275
C -1.02416 0.069076 0.285914	C -1.02395 0.068066 0.287915	C -1.02408 0.065981 0.288503
C -2.61596 -1.6998 -0.17982	C -2.61715 -1.6982 -0.18252	C -2.62005 -1.69739 -0.18575
C -3.64512 -0.7735 -0.34821	C -3.64558 -0.77081 -0.34933	C -3.64753 -0.76871 -0.34927
C -3.33689 0.59349 -0.17972	C -3.33631 0.595374 -0.1774	C -3.33656 0.596812 -0.1737
C -2.04041 1.016874 0.141235	C -2.03968 1.016942 0.145191	C -2.03937 1.016162 0.149296
C -5.05177 -1.19391 -0.69062	C -5.05228 -1.1894 -0.69385	C -5.05485 -1.18469 -0.6941
C 0.631295 -0.54932 1.934856	C 0.635324 -0.55288 1.934897	C 0.642785 -0.55751 1.931228
O 4.176536 2.228559 -0.47532	O 4.216301 2.110112 -0.55325	O 4.173953 2.233548 -0.60622
H 0.867368 0.189651 -1.42325	H 0.865306 0.183309 -1.42258	H 0.867849 0.178764 -1.42239
C 3.347521 -0.22921 -1.99185	C 3.344423 -0.21778 -2.00674	C 3.343083 -0.21857 -2.00958
C 3.771431 -0.50007 0.45208	C 3.778731 -0.5042 0.436002	C 3.780947 -0.50273 0.42714
O 0.470742 -1.9467 1.642556	O 0.463888 -1.94909 1.64446	O 0.457786 -1.95348 1.64258
O 2.079651 -2.70322 -0.42899	O 2.076061 -2.71118 -0.42033	O 2.071588 -2.71986 -0.40854
O -4.39395 1.446435 -0.34984	O -4.39286 1.449753 -0.34602	O -4.39193 1.452521 -0.33933
C -4.1778 2.838642 -0.19324	C -4.17541 2.841249 -0.18768	C -4.17223 2.844122 -0.1821
H 2.369755 3.230734 0.983621	H 2.356336 3.229129 0.989745	H 2.344893 3.245838 0.950821
H 2.778091 1.694046 1.729535	H 2.779099 1.690352 1.726297	H 2.764613 1.722585 1.725111
H 2.260047 2.122926 -1.25122	H 2.268263 2.117764 -1.26042	H 2.293654 2.128716 -1.27304
H 0.255501 2.394976 0.139535	H 0.255571 2.388929 0.134549	H 0.245143 2.38178 0.12704
H 0.295581 2.109902 1.871935	H 0.292331 2.110624 1.868457	H 0.288976 2.110888 1.861585
H 0.664227 -2.04001 -1.68997	H 0.670378 -2.04349 -1.68837	H 0.677026 -2.04791 -1.68979
H -0.3574 -3.23495 0.294884	H -0.36076 -3.23581 0.293573	H -0.36694 -3.23743 0.28894
H -2.83508 -2.75967 -0.29488	H -2.83687 -2.75765 -0.30014	H -2.84053 -2.75635 -0.3061
H -1.83351 2.070487 0.279556	H -1.83273 2.070252 0.285898	H -1.83204 2.068939 0.292486
H -5.12089 -2.28225 -0.78059	H -5.12241 -2.27761 -0.78458	H -5.12586 -2.2724 -0.7901
H -5.76491 -0.86535 0.074742	H -5.76613 -0.86049 0.070692	H -5.7675 -0.85905 0.072957
H -5.38381 -0.74821 -1.63592	H -5.38247 -0.74289 -1.63943	H -5.38578 -0.73316 -1.63697

H 1.623932 -0.42253 2.375108	H 1.632633 -0.43191 2.366634	H 1.646614 -0.4445 2.351181
H -0.11085 -0.24874 2.688096	H -0.09944 -0.24792 2.693786	H -0.08058 -0.24904 2.699262
H 4.548758 2.024453 -1.34685	H 4.223864 3.076049 -0.47319	H 4.679446 2.030252 0.196037
H 3.273672 -1.30638 -2.16155	H 3.314176 -1.29903 -2.16639	H 3.356845 -1.3024 -2.15522
H 4.407785 0.036494 -2.09545	H 4.377469 0.122655 -2.12098	H 4.358506 0.166742 -2.1418
H 2.788065 0.287205 -2.78339	H 2.744341 0.266689 -2.78831	H 2.715587 0.228823 -2.79126
H 3.365632 -0.52267 1.466224	H 3.396035 -0.4894 1.460148	H 3.463887 -0.38389 1.467251
H 4.027222 -1.52136 0.170264	H 3.991065 -1.53921 0.170864	H 3.904999 -1.56546 0.228004
H 4.691567 0.091542 0.486195	H 4.719979 0.052108 0.431787	H 4.771944 -0.04283 0.330378
H 2.163661 -2.73925 0.541295	H 2.155977 -2.74664 0.550254	H 2.117565 -2.77902 0.563563
H -5.14371 3.313704 -0.37526	H -5.14073 3.317665 -0.36944	H -5.13696 3.321647 -0.36388
H -3.83954 3.084094 0.822279	H -3.83727 3.085317 0.828308	H -3.83353 3.088306 0.833615
H -3.44521 3.218609 -0.91777	H -3.44216 3.221474 -0.9115	H -3.43873 3.222435 -0.90651

Table S6. Conformers and Boltzmann populations of 6β-OH isomer of **2**.

**6β1** (26.78%)	**6β2** (23.81%)	**6β3** (30.93%)
C 2.41495 2.122268 0.732579	C 2.409895 2.128151 0.703814	C 2.403378 2.151761 0.665434
C 3.062606 1.470365 -0.4864	C 3.078819 1.451477 -0.49812	C 3.099936 1.450906 -0.50734
C 2.879224 -0.07758 -0.5172	C 2.886717 -0.08757 -0.51506	C 2.889132 -0.0932 -0.51047
C 1.341606 -0.37033 -0.43396	C 1.344788 -0.36853 -0.4431	C 1.345518 -0.3649 -0.45634
C 0.519659 0.395444 0.676883	C 0.520027 0.39994 0.661932	C 0.52012 0.408768 0.641595
C 0.900373 1.882055 0.73384	C 0.895054 1.888075 0.701283	C 0.890448 1.898443 0.660869
C 0.971197 -1.887 -0.39181	C 0.963323 -1.88253 -0.40824	C 0.952438 -1.87661 -0.43024
C -0.09996 -2.0937 0.699515	C -0.09646 -2.08879 0.694772	C -0.09046 -2.08237 0.689294
C -1.2832 -1.21951 0.383165	C -1.2833 -1.21731 0.385016	C -1.28227 -1.21455 0.389771
C -0.95029 0.141486 0.34906	C -0.95121 0.143424 0.342459	C -0.9524 0.146073 0.333095
C -2.59225 -1.62953 0.126384	C -2.59375 -1.62916 0.13878	C -2.5945 -1.63022 0.159255
C -3.596 -0.70381 -0.16328	C -3.60005 -0.70541 -0.14827	C -3.6045 -0.71002 -0.12572
C -3.24891 0.664282 -0.18141	C -3.25378 0.662663 -0.17523	C -3.26013 0.658356 -0.168
C -1.93843 1.089643 0.075805	C -1.94193 1.089828 0.071259	C -1.94659 1.089105 0.062749
C -5.01593 -1.12586 -0.44355	C -5.02146 -1.12963 -0.41749	C -5.02785 -1.13802 -0.37799
C 0.701502 -0.29787 2.05435	C 0.711827 -0.2876 2.040777	C 0.722637 -0.2716 2.022158
O 4.431452 1.874144 -0.49419	O 4.480329 1.724563 -0.53555	O 4.47812 1.818792 -0.58064
H 0.933855 0.00552 -1.38304	H 0.949157 0.011356 -1.39533	H 0.965076 0.020122 -1.41249
C 3.381466 -0.60574 -1.88174	C 3.404148 -0.62828 -1.86768	C 3.424878 -0.65386 -1.84637
C 3.738216 -0.73889 0.582011	C 3.731628 -0.73934 0.602921	C 3.711117 -0.73732 0.630977

O 0.502639 -1.70909 1.952231
O 0.550747 -2.39549 -1.654
O -4.28174 1.51598 -0.46451
C -4.02609 2.910272 -0.49547
H 2.623677 3.197651 0.702464
H 2.882978 1.742124 1.648069
H 2.559905 1.872945 -1.3852
H 0.476461 2.386612 -0.14526
H 0.438377 2.35318 1.611869
H 1.836097 -2.49454 -0.11934
H -0.35842 -3.15089 0.794456
H -2.84278 -2.68831 0.155747
H -1.69662 2.145266 0.066664
H -5.11586 -2.21405 -0.38781
H -5.71447 -0.67861 0.273587
H -5.34207 -0.80005 -1.43854
H 1.700117 -0.13855 2.46941
H -0.02487 0.128635 2.762495
H 4.827638 1.563828 -1.32278
H 3.176119 -1.67389 -1.99835
H 4.466338 -0.47226 -1.98387
H 2.891192 -0.08861 -2.71626
H 3.42641 -0.48133 1.595676
H 3.722252 -1.82995 0.500866
H 4.775614 -0.41044 0.471247
H -0.28284 -1.94655 -1.87548
H -4.97969 3.383773 -0.73657
H -3.67456 3.278777 0.477347
H -3.28813 3.169382 -1.26632

6β4 (4.42%)

C 2.397297 2.12492 0.749566
C 3.038484 1.489432 -0.48048
C 2.876214 -0.06061 -0.51353
C 1.341977 -0.37545 -0.42504
C 0.51017 0.384762 0.683905
C 0.885383 1.872422 0.754031
C 0.9999 -1.88722 -0.36382

O 0.513672 -1.69933 1.942546
O 0.520041 -2.37804 -1.66721
O -4.28913 1.51266 -0.45558
C -4.03407 2.906488 -0.49639
H 2.604082 3.20976 0.661782
H 2.872215 1.769915 1.63057
H 2.603871 1.847582 -1.41466
H 0.474815 2.376808 -0.18864
H 0.427491 2.372609 1.56902
H 1.827077 -2.49936 -0.1548
H -0.35294 -3.14591 0.795175
H -2.84306 -2.68803 0.173717
H -1.70127 2.145663 0.055369
H -5.12024 -2.21762 -0.35606
H -5.71579 -0.67951 0.301962
H -5.35409 -0.80878 -1.41195
H 1.714318 -0.12861 2.446003
H -0.00933 0.139405 2.754162
H 4.58976 2.686782 -0.58003
H 3.278016 -1.71327 -1.9381
H 4.46527 -0.39202 -1.9871
H 2.858272 -0.18139 -2.70763
H 3.50124 -0.36218 1.601722
H 3.598744 -1.82488 0.623668
H 4.789302 -0.53556 0.417577
H -0.31082 -1.91787 -1.875
H -4.98934 3.378561 -0.73378
H -3.67602 3.28092 0.471865
H -3.30137 3.161366 -1.27375

6β5 (3.81%)

C 2.392497 2.13196 0.717998
C 3.056073 1.469135 -0.49364
C 2.884558 -0.07214 -0.51008
C 1.345306 -0.37344 -0.43308
C 0.510763 0.389942 0.669295
C 0.880398 1.879274 0.719571
C 0.9901 -1.88233 -0.38186

O 0.538306 -1.68598 1.92648
O 0.485127 -2.35651 -1.68586
O -4.29877 1.504335 -0.44617
C -4.04563 2.898247 -0.50466
H 2.602537 3.227161 0.592031
H 2.846749 1.820894 1.615105
H 2.671859 1.843328 -1.43983
H 0.471952 2.370987 -0.23835
H 0.416758 2.394702 1.518743
H 1.815805 -2.50339 -0.19948
H -0.34374 -3.13937 0.798545
H -2.84198 -2.6891 0.204538
H -1.7072 2.144928 0.035457
H -5.12487 -2.22554 -0.30649
H -5.71578 -0.68248 0.344152
H -5.37001 -0.82579 -1.37192
H 1.725655 -0.10437 2.423186
H 0.001359 0.150063 2.738343
H 4.887142 1.584623 0.267464
H 3.354568 -1.74588 -1.88312
H 4.472413 -0.36681 -1.97785
H 2.856066 -0.26102 -2.69736
H 3.573185 -0.24884 1.60008
H 3.462695 -1.79278 0.769323
H 4.778601 -0.69173 0.387187
H -0.34948 -1.89392 -1.87238
H -5.00363 3.366497 -0.73836
H -3.67922 3.283486 0.456131
H -3.32047 3.14509 -1.29148

6β6 (2.07%)

C 2.389043 2.145933 0.692129
C 3.059722 1.465235 -0.50605
C 2.885895 -0.07536 -0.49505
C 1.346594 -0.37255 -0.43193
C 0.509586 0.396857 0.663077
C 0.877381 1.887366 0.699915
C 0.982554 -1.88968 -0.38414

C	-0.1138	-2.09666	0.686462	C	-0.11034	-2.09126	0.682831	C	-0.10771	-2.09144	0.682126
C	-1.29108	-1.22292	0.366394	C	-1.29132	-1.2203	0.369902	C	-1.28873	-1.21913	0.36806
C	-0.95799	0.135377	0.345669	C	-0.95887	0.137709	0.339541	C	-0.95953	0.139351	0.334337
C	-2.59808	-1.63137	0.108249	C	-2.59974	-1.6308	0.12296	C	-2.59642	-1.63293	0.12321
C	-3.60088	-0.70422	-0.17741	C	-3.6051	-0.70585	-0.16074	C	-3.60408	-0.71015	-0.15902
C	-3.25327	0.663272	-0.18842	C	-3.25819	0.661577	-0.1819	C	-3.26083	0.658154	-0.18161
C	-1.94428	1.0866	0.075886	C	-1.94771	1.086949	0.071183	C	-1.95071	1.086887	0.066938
C	-5.02033	-1.1242	-0.46332	C	-5.02617	-1.12826	-0.43484	C	-5.02439	-1.13654	-0.43125
C	0.683696	-0.31538	2.059673	C	0.693968	-0.30388	2.046772	C	0.692851	-0.29294	2.041907
O	4.402085	1.911484	-0.50582	O	4.453579	1.761756	-0.54867	O	4.457953	1.753555	-0.55812
H	0.91784	-0.02853	-1.37603	H	0.934365	-0.02041	-1.38731	H	0.941549	-0.01048	-1.38635
C	3.384065	-0.57755	-1.87997	C	3.406788	-0.60491	-1.86378	C	3.426412	-0.6343	-1.83182
C	3.75071	-0.70866	0.582052	C	3.744971	-0.70926	0.604811	C	3.73475	-0.69403	0.639277
O	0.472221	-1.72645	1.953514	O	0.485733	-1.71575	1.943943	O	0.493232	-1.70486	1.938307
O	0.61366	-2.3032	-1.67424	O	0.576087	-2.28012	-1.6889	O	0.478245	-2.38417	-1.62255
O	-4.28649	1.517897	-0.46908	O	-4.29398	1.514342	-0.46036	O	-4.29971	1.508726	-0.45786
C	-4.02575	2.910229	-0.50371	C	-4.03368	2.906071	-0.50683	C	-4.04363	2.900853	-0.50132
H	2.597412	3.202185	0.728615	H	2.577499	3.215478	0.682527	H	2.569415	3.229379	0.636992
H	2.873809	1.738799	1.658261	H	2.863575	1.770554	1.639163	H	2.86122	1.803834	1.619878
H	2.518773	1.888358	-1.3708	H	2.564528	1.860288	-1.40324	H	2.57356	1.842394	-1.42472
H	0.456071	2.382403	-0.11894	H	0.454507	2.371965	-0.16513	H	0.446927	2.371139	-0.18747
H	0.421662	2.331611	1.637366	H	0.411253	2.353433	1.592068	H	0.41077	2.369012	1.569573
H	1.865639	-2.46827	-0.02188	H	1.855656	-2.47579	-0.06286	H	1.847045	-2.4857	-0.06732
H	-0.38016	-3.15495	0.779611	H	-0.37448	-3.14942	0.782252	H	-0.36678	-3.14801	0.778994
H	-2.84976	-2.69028	0.128014	H	-2.85032	-2.68981	0.149554	H	-2.84141	-2.69282	0.145914
H	-1.70262	2.142236	0.074563	H	-1.70723	2.142847	0.062206	H	-1.71293	2.143445	0.056799
H	-5.12156	-2.21256	-0.40999	H	-5.12623	-2.21638	-0.37478	H	-5.12042	-2.225	-0.37258
H	-5.72206	-0.67769	0.251313	H	-5.72353	-0.67828	0.281918	H	-5.72198	-0.68964	0.287273
H	-5.34174	-0.79705	-1.45945	H	-5.35436	-0.80698	-1.43067	H	-5.35513	-0.81519	-1.42622
H	1.682723	-0.16651	2.478392	H	1.696195	-0.15398	2.456846	H	1.693037	-0.13767	2.45544
H	-0.04163	0.113305	2.767036	H	-0.0275	0.124209	2.7587	H	-0.03243	0.133653	2.751036
H	4.787846	1.616586	-1.34486	H	4.548006	2.725621	-0.59079	H	4.556825	2.717292	-0.59415
H	3.187413	-1.64686	-1.99961	H	3.29002	-1.69101	-1.93447	H	3.385845	-1.73104	-1.8508
H	4.467746	-0.43323	-1.98081	H	4.465678	-0.35889	-1.98362	H	4.470669	-0.34181	-1.97179
H	2.887224	-0.06373	-2.7123	H	2.854715	-0.1637	-2.70218	H	2.853169	-0.25304	-2.68694
H	3.410856	-0.49611	1.59727	H	3.48405	-0.36835	1.609142	H	3.503126	-0.28765	1.625738
H	3.789532	-1.79671	0.470822	H	3.663062	-1.80016	0.597705	H	3.602539	-1.7784	0.693548
H	4.772209	-0.32723	0.495359	H	4.794185	-0.45271	0.437643	H	4.792099	-0.4929	0.448257

H 0.472913 -3.26345 -1.65103
H -4.97677 3.387213 -0.74886
H -3.67537 3.281432 0.468792
H -3.28438 3.164507 -1.27305

6β7 (1.47%)

C 2.39374 2.142518 0.710214
C 3.055623 1.477677 -0.49443
C 2.884014 -0.07226 -0.49257
C 1.345875 -0.37423 -0.43049
C 0.510197 0.39433 0.667645
C 0.882738 1.883379 0.715477
C 0.981524 -1.89202 -0.38261
C -0.10938 -2.09395 0.683195
C -1.28911 -1.21973 0.36833
C -0.95836 0.138497 0.334983
C -2.59686 -1.63201 0.121071
C -3.60279 -0.70788 -0.16293
C -3.25786 0.660126 -0.18503
C -1.9476 1.087311 0.065698
C -5.02324 -1.13239 -0.43734
C 0.685622 -0.29823 2.046352
O 4.422377 1.891531 -0.49563
H 0.938029 -0.01056 -1.38356
C 3.419445 -0.62848 -1.83295
C 3.735981 -0.69679 0.633641
O 0.492901 -1.71062 1.939225
O 0.474688 -2.38821 -1.61973
O -4.29503 1.511776 -0.46306
C -4.03765 2.904022 -0.5037
H 2.58883 3.219646 0.659918
H 2.866015 1.786656 1.633072
H 2.557741 1.860597 -1.40403
H 0.452101 2.377365 -0.16612
H 0.417642 2.356848 1.590454
H 1.846495 -2.48755 -0.06484
H -0.36966 -3.15048 0.77746
H -2.84338 -2.69156 0.143495

H 0.449798 -3.24249 -1.68006
H -4.98639 3.381405 -0.74881
H -3.67659 3.28453 0.460524
H -3.29765 3.154957 -1.28313

6β8 (4.47%)

C 2.386029 2.155687 0.677909
C 3.077885 1.467584 -0.50411
C 2.887635 -0.07929 -0.50457
C 1.346329 -0.37046 -0.44499
C 0.51105 0.398239 0.649895
C 0.876075 1.889241 0.678838
C 0.978087 -1.87704 -0.40444
C -0.10541 -2.0851 0.678138
C -1.29109 -1.2175 0.375723
C -0.96006 0.140247 0.330936
C -2.6014 -1.63138 0.14465
C -3.61002 -0.70966 -0.13755
C -3.2643 0.657884 -0.17453
C -1.95193 1.086425 0.063045
C -5.03326 -1.13534 -0.39464
C 0.704791 -0.28842 2.029088
O 4.450476 1.853901 -0.59407
H 0.950854 -0.01021 -1.40295
C 3.426808 -0.6339 -1.84139
C 3.726264 -0.70748 0.633193
O 0.510501 -1.70298 1.92851
O 0.53945 -2.25546 -1.70834
O -4.3029 1.507084 -0.45135
C -4.04378 2.89862 -0.51613
H 2.575072 3.233327 0.609458
H 2.838748 1.822445 1.622449
H 2.632985 1.854446 -1.43088
H 0.451255 2.365075 -0.2153
H 0.401431 2.375831 1.541598
H 1.843773 -2.48324 -0.10921
H -0.36637 -3.14314 0.786588
H -2.8508 -2.69035 0.182411

H 1.186318 -2.30331 -2.27963
H -4.99826 3.374438 -0.73934
H -3.68467 3.278054 0.465929
H -3.31046 3.154425 -1.27902

6β9 (2.47%)

C 2.384377 2.164814 0.661537
C 3.080005 1.465629 -0.51174
C 2.886535 -0.08027 -0.49504
C 1.346082 -0.36982 -0.44332
C 0.50949 0.404635 0.646062
C 0.874245 1.896089 0.665668
C 0.972857 -1.88547 -0.40055
C -0.10364 -2.08536 0.680063
C -1.28869 -1.21615 0.374145
C -0.96089 0.142115 0.327221
C -2.59792 -1.63309 0.143029
C -3.60834 -0.7133 -0.13861
C -3.26622 0.65515 -0.17566
C -1.95457 1.086857 0.059444
C -5.03042 -1.14272 -0.39625
C 0.702113 -0.2779 2.026848
O 4.454178 1.846103 -0.60187
H 0.9519 -0.00319 -1.40054
C 3.437324 -0.64981 -1.82169
C 3.718755 -0.69893 0.65292
O 0.514124 -1.69209 1.926974
O 0.449874 -2.36968 -1.63469
O -4.30746 1.502335 -0.45129
C -4.05226 2.894322 -0.51205
H 2.571898 3.241906 0.581627
H 2.837031 1.842604 1.609855
H 2.63854 1.84419 -1.44379
H 0.447276 2.366501 -0.23033
H 0.401452 2.388201 1.526209
H 1.838193 -2.48967 -0.10079
H -0.36017 -3.1417 0.785589
H -2.84181 -2.69298 0.17566

H	-1.70794	2.143419	0.056248	H	-1.71237	2.142304	0.042359	H	-1.71742	2.143347	0.03836
H	-5.1209	-2.22071	-0.37878	H	-5.13215	-2.22293	-0.32382	H	-5.12541	-2.2307	-0.32787
H	-5.72127	-0.68449	0.280118	H	-5.72415	-0.67919	0.324452	H	-5.72261	-0.69018	0.32394
H	-5.35208	-0.81054	-1.43277	H	-5.37072	-0.82315	-1.39023	H	-5.36893	-0.82964	-1.39121
H	1.680495	-0.13852	2.470663	H	1.707629	-0.13046	2.43506	H	1.702959	-0.11512	2.436584
H	-0.04764	0.125136	2.749028	H	-0.01692	0.134259	2.743783	H	-0.02295	0.144993	2.738192
H	4.814932	1.630536	-1.34227	H	4.867214	1.646263	0.257084	H	4.863954	1.663076	0.258343
H	3.32672	-1.72055	-1.8795	H	3.366483	-1.72677	-1.87641	H	3.424136	-1.74739	-1.82608
H	4.488358	-0.41366	-1.95561	H	4.471449	-0.33741	-1.97513	H	4.472994	-0.32941	-1.968
H	2.886417	-0.20146	-2.6925	H	2.851204	-0.24838	-2.69069	H	2.855115	-0.29436	-2.68161
H	3.44438	-0.36699	1.631808	H	3.553986	-0.2494	1.611311	H	3.556062	-0.21452	1.619493
H	3.6822	-1.78943	0.62114	H	3.529518	-1.77723	0.742815	H	3.501063	-1.7613	0.789543
H	4.781921	-0.40848	0.494376	H	4.792795	-0.605	0.402388	H	4.7864	-0.61928	0.418313
H	1.174105	-2.2963	-2.28424	H	0.392769	-3.21502	-1.70657	H	1.151958	-2.29547	-2.29908
H	-4.99148	3.378676	-0.74264	H	-4.99894	3.370525	-0.75493	H	-5.00893	3.364717	-0.74798
H	-3.68012	3.279077	0.464828	H	-3.67836	3.288161	0.443634	H	-3.68625	3.282095	0.448302
H	-3.30287	3.158208	-1.2796	H	-3.31493	3.138736	-1.30178	H	-3.32517	3.139317	-1.29802

Table S7. DP4+ analysis table for compound **6** (isomer **1**: 2α,10α-OH; isomer **2**: 2β,10α-OH; isomer **3**: 2α,10β-OH; isomer **4**: 2β,10β-OH).

Functional	Solvent?	Basis Set
mPW1PW91	PCM	6-31+G(d,p)

Isomer Nº			1	2	3	4
DP4+ (%)		H data	100.00%	0.00%	0.00%	0.00%
		C data	100.00%	0.00%	0.00%	0.00%
		All data	100.00%	0.00%	0.00%	0.00%
Type	sp2?	Exp	1	2	3	4
C		72.7	120.7953331	127.020847	126.211219	124.442926
C		77.7	116.5362693	116.694796	114.929367	119.014128
C		37.3	153.9252959	152.490488	155.170259	154.171779
C		50	143.3648261	142.043119	140.297437	140.103775
C		79.7	112.4088318	123.48581	103.278112	108.419972
C		39.1	156.0973405	152.299242	152.504518	155.529593
C	x	127.8	68.74998702	69.7097748	64.2909436	66.5446592
C	x	131.4	65.66996402	67.949571	66.301696	66.5728773
C	x	128.5	69.9068492	71.40063	67.3098119	68.9206392
C	x	134.2	61.83062617	63.0448967	60.1248338	61.1021805
C		51	143.6634072	143.901219	144.300485	145.487762
C	x	132.2	66.47120351	64.8270532	68.3394601	66.909215
C	x	124.9	72.13857427	72.4637203	72.0923715	72.0763857
C	x	157.1	41.93049285	41.8105599	42.7036606	42.2558289
C	x	112.1	88.01266816	87.1747742	88.9495896	87.9801737
C		15.7	177.3512926	177.510522	177.233878	177.381132
C		22.6	173.8083769	174.504133	169.285563	169.615151
C		28.3	168.20238	168.965692	173.345005	169.563666
C		55.4	142.0480025	141.99879	142.029988	142.002335
H		2.39	28.98027259	28.7309236	29.9403773	29.4400876
H		4	27.65355522	27.2137399	27.5696957	27.5766669
H		3.63	27.98681306	27.9937538	28.0491576	28.0410469
H		2.25	29.26460226	29.6443504	29.9015043	29.3582693
H		1.95	29.74291214	29.8047291	29.5093268	29.7069854
H	x	5.85	25.35061201	25.6049567	24.9943284	25.2235858
H	x	6.61	24.488405	24.7195182	24.4952257	24.5718205
H		2.95	28.65257945	28.3044752	28.8461367	28.9933741
H		2.68	28.88944676	28.8944254	29.2406099	28.6411592
H	x	6.96	24.29783461	24.3481814	24.2194392	24.2537939
H	x	6.58	24.78408735	24.84541	24.6651408	24.735873
H		2.18	29.36601359	29.3816894	29.343001	29.338541
H		1.25	30.41075391	30.5438934	30.6497439	30.6399623
H		1.03	30.63006629	30.423395	30.4301909	30.4725995
H		3.84	27.63462954	27.6419699	27.6233845	27.6472857

Table S8. Conformers and Boltzmann populations of 2α,10α-OH isomer of **6**.

**2α10α1** (21.64%)	**2α10α2** (36.78%)	**2α10α3** (11.89%)
C -3.16462 -1.77871 0.386831	C -3.1668 -1.79014 0.377929	C 2.885733 -1.74389 0.546028
C -3.93889 -0.48279 0.057025	C -3.9383 -0.49699 0.049076	C 3.042049 -0.44262 1.371303
C -3.1218 0.833814 0.161924	C -3.11862 0.825041 0.171262	C 2.72213 0.874681 0.608689
C -1.75105 0.662447 -0.60737	C -1.75089 0.658389 -0.60123	C 1.346458 0.709815 -0.12276
C -0.97302 -0.63909 -0.26787	C -0.97167 -0.64403 -0.26553	C 1.207388 -0.58222 -0.98163
C -1.88425 -1.86133 -0.45051	C -1.8818 -1.86707 -0.45189	C 1.502069 -1.81193 -0.11545
C -0.922 1.926428 -0.55061	C -0.92381 1.923745 -0.54861	C 0.874181 1.950583 -0.85179
C 0.402445 2.147337 -0.4273	C 0.400376 2.146818 -0.4263	C -0.42957 2.218508 -1.05778
C 1.553877 1.241658 -0.31841	C 1.552786 1.242668 -0.31789	C -1.51851 1.300499 -0.69204
C 1.526589 -0.12578 -0.65635	C 1.526626 -0.12469 -0.65671	C -1.38321 -0.09338 -0.86827
C 0.275419 -0.78658 -1.18568	C 0.275838 -0.7867 -1.18538	C -0.20315 -0.6524 -1.63402
C 2.775568 1.789206 0.128073	C 2.774011 1.791681 0.12822	C -2.71762 1.7978 -0.15059
C 3.941039 1.048175 0.270742	C 3.940256 1.051869 0.270229	C -3.75512 0.973069 0.268851
C 3.882823 -0.32243 -0.05587	C 3.883298 -0.31881 -0.0568	C -3.58347 -0.42014 0.122927
C 2.690275 -0.89053 -0.50915	C 2.691246 -0.88808 -0.51004	C -2.42 -0.93826 -0.45063
C 5.230606 1.660584 0.754187	C 5.229382 1.665317 0.753477	C -5.02661 1.521445 0.864485
C -2.94288 1.231586 1.644681	C -2.94678 1.219832 1.654966	C 3.90318 1.235619 -0.32223
C -3.96015 1.953611 -0.50361	C -3.95445 1.948812 -0.49197	C 2.595409 2.021053 1.638647
H -2.05244 0.536415 -1.65872	H -2.05776 0.527741 -1.65087	H 0.618234 0.54363 0.682832
O -0.56329 -0.69389 1.115971	O -0.55975 -0.69979 1.117301	O 2.171687 -0.6457 -2.06154
O -4.41597 -0.58005 -1.2953	O -4.40574 -0.70428 -1.29402	O 2.147647 -0.51551 2.49253
O -2.92403 -1.94502 1.776737	O -2.92859 -1.94312 1.771176	O 3.943422 -1.93934 -0.38306
O 5.051633 -1.01179 0.101615	O 5.05264 -1.00684 0.100243	O -4.63048 -1.18432 0.558575
C 5.069524 -2.39759 -0.20263	C 5.071801 -2.39301 -0.2033	C -4.53553 -2.59448 0.442506
H -3.82146 -2.61559 0.1006	H -3.8261 -2.61536 0.084454	H 2.96848 -2.57636 1.262576
H -4.78973 -0.40651 0.752055	H -4.79328 -0.43323 0.739492	H 4.08396 -0.38826 1.723691
H -2.15192 -1.94327 -1.50937	H -2.14539 -1.95343 -1.51151	H 0.731799 -1.88154 0.659978
H -1.3278 -2.764 -0.17173	H -1.32398 -2.76797 -0.16947	H 1.442823 -2.71601 -0.7335
H -1.51778 2.831964 -0.63226	H -1.51952 2.82974 -0.62993	H 1.609242 2.693654 -1.1533
H 0.69026 3.198277 -0.38792	H 0.686755 3.198195 -0.38761	H -0.7136 3.181965 -1.48135
H 0.031582 -0.3843 -2.1782	H 0.03005 -0.38323 -2.17696	H -0.12801 -0.08764 -2.57255
H 0.48007 -1.85429 -1.32225	H 0.481671 -1.85376 -1.32413	H -0.39561 -1.6935 -1.9175
H 2.80529 2.848277 0.377345	H 2.802675 2.85071 0.377864	H -2.83187 2.874319 -0.03771
H 2.650065 -1.94384 -0.76371	H 2.652028 -1.94132 -0.76494	H -2.31272 -2.00685 -0.6029
H 6.029827 1.545249 0.012421	H 6.028504 1.550814 0.011482	H -5.18154 1.151248 1.885008
H 5.102238 2.727767 0.958881	H 5.100147 2.732345 0.95847	H -5.00135 2.614913 0.895933

H	5.58475	1.173849	1.670732
H	-2.49175	0.448939	2.250543
H	-2.31934	2.129501	1.72221
H	-3.91905	1.468667	2.084392
H	-3.56175	2.946225	-0.26898
H	-4.01166	1.841219	-1.58922
H	-4.98681	1.922047	-0.12069
H	-0.01437	0.085224	1.304508
H	-4.97273	-1.37243	-1.34983
H	-2.04533	-1.55882	1.946906
H	6.087925	-2.73466	-0.0007
H	4.369123	-2.95819	0.430057
H	4.828937	-2.58186	-1.25789

H	5.584155	1.17864	1.669801
H	-2.48669	0.43782	2.254153
H	-2.33511	2.125437	1.740444
H	-3.92638	1.439835	2.096903
H	-3.56927	2.943062	-0.24642
H	-3.97976	1.863024	-1.58395
H	-4.98722	1.919365	-0.1179
H	0.00332	0.070126	1.301837
H	-5.09082	-0.04529	-1.47677
H	-2.04552	-1.56837	1.940693
H	6.090552	-2.72888	-0.00123
H	4.371972	-2.95375	0.429806
H	4.831316	-2.57794	-1.25842

H	-5.905	1.212655	0.285342
H	4.151844	0.45108	-1.03366
H	3.695094	2.156229	-0.87992
H	4.798709	1.426447	0.280752
H	2.466718	2.983846	1.131518
H	1.749798	1.868565	2.312422
H	3.504113	2.085382	2.249401
H	2.126478	0.195645	-2.54561
H	2.382667	-1.30837	2.999275
H	3.638103	-1.55408	-1.22458
H	-4.4472	-2.90877	-0.60592
H	-3.68215	-2.98936	1.009399
H	-5.46209	-2.99234	0.860751

2α10α4 (24.00%)

C	2.877282	-1.75026	0.560856
C	3.026565	-0.45009	1.381388
C	2.719136	0.8701	0.603745
C	1.347829	0.705597	-0.13225
C	1.209908	-0.59048	-0.98547
C	1.500554	-1.81762	-0.11383
C	0.877655	1.944782	-0.86487
C	-0.4258	2.214339	-1.07146
C	-1.51524	1.298335	-0.70264
C	-1.37982	-0.09642	-0.87343
C	-0.20078	-0.6593	-1.63815
C	-2.71406	1.79811	-0.16268
C	-3.7508	0.975079	0.261838
C	-3.57837	-0.41893	0.123227
C	-2.4157	-0.93936	-0.45002
C	-5.02215	1.525627	0.855758
C	3.909084	1.228961	-0.31515
C	2.583913	2.023026	1.626314
H	0.618271	0.538304	0.672425
O	2.17486	-0.65658	-2.06402
O	2.131094	-0.63608	2.487842
O	3.943214	-1.93169	-0.36356
O	-4.62357	-1.18123	0.564985

2α10α14 (1.78%)

C	2.873925	-1.74938	0.569382
C	3.032847	-0.44276	1.386639
C	2.720967	0.875234	0.605483
C	1.350056	0.708895	-0.13629
C	1.20852	-0.5899	-0.98772
C	1.498864	-1.81795	-0.11484
C	0.878525	1.946457	-0.871
C	-0.4256	2.214222	-1.07511
C	-1.51398	1.298425	-0.70184
C	-1.38044	-0.0965	-0.87404
C	-0.20232	-0.65898	-1.64048
C	-2.71066	1.798548	-0.15697
C	-3.74715	0.976032	0.269061
C	-3.57773	-0.41826	0.126457
C	-2.41642	-0.93939	-0.44948
C	-5.01624	1.527259	0.867024
C	3.90507	1.224905	-0.32576
C	2.593282	2.03119	1.623041
H	0.602081	0.555534	0.658467
O	2.173716	-0.65952	-2.06206
O	2.259569	-0.56005	2.589855
O	3.933815	-1.95189	-0.35873
O	-4.62316	-1.17927	0.567907

2α10α15 (1.32%)

C	2.853166	-1.74052	0.594148
C	3.022577	-0.42171	1.388017
C	2.722418	0.881474	0.59103
C	1.353237	0.710072	-0.15062
C	1.204251	-0.59686	-0.97373
C	1.466649	-1.80817	-0.06445
C	0.886572	1.937971	-0.90201
C	-0.4153	2.213897	-1.09656
C	-1.50517	1.303533	-0.70709
C	-1.37685	-0.09073	-0.88166
C	-0.19452	-0.65113	-1.64441
C	-2.69775	1.802942	-0.15487
C	-3.73515	0.98083	0.271902
C	-3.56989	-0.41248	0.12452
C	-2.41161	-0.93417	-0.4563
C	-5.0003	1.5336	0.87706
C	3.911745	1.223222	-0.33582
C	2.591045	2.049054	1.596822
H	0.623051	0.559985	0.656184
O	2.190758	-0.59475	-2.04726
O	2.122488	-0.45911	2.507153
O	3.903078	-1.96534	-0.33699
O	-4.61684	-1.17395	0.56778

C -4.52497 -2.59266 0.462667	C -4.53495 -2.59032 0.449222	C -4.52637 -2.58374 0.451238
H 2.954618 -2.56919 1.285333	H 2.947853 -2.56462 1.298592	H 2.93453 -2.55953 1.324991
H 4.065575 -0.40382 1.742331	H 4.069357 -0.39702 1.736441	H 4.063378 -0.37212 1.743663
H 0.72493 -1.8911 0.655982	H 0.703512 -1.90678 0.637181	H 0.694527 -1.84444 0.712249
H 1.448054 -2.72186 -0.73258	H 1.451934 -2.72233 -0.73311	H 1.393095 -2.73587 -0.64853
H 1.614836 2.683725 -1.17231	H 1.615921 2.685365 -1.17595	H 1.630734 2.650583 -1.24766
H -0.70851 3.176275 -1.49951	H -0.70978 3.175956 -1.50216	H -0.69849 3.164859 -1.54761
H -0.12558 -0.09746 -2.57852	H -0.12659 -0.09631 -2.58007	H -0.09385 -0.07043 -2.56988
H -0.39486 -1.70095 -1.91796	H -0.396 -1.70046 -1.92129	H -0.40221 -1.69118 -1.93196
H -2.82878 2.875206 -0.05528	H -2.82384 2.875491 -0.0476	H -2.80813 2.879728 -0.04114
H -2.30785 -2.00864 -0.59635	H -2.31184 -2.00843 -0.60061	H -2.30761 -2.00352 -0.60635
H -5.17657 1.159706 1.877904	H -5.16902 1.159202 1.888594	H -5.14946 1.163052 1.898327
H -4.99749 2.619276 0.882266	H -4.98985 2.620696 0.895947	H -4.97047 2.62691 0.909272
H -5.90069 1.213913 0.278442	H -5.89617 1.217897 0.290668	H -5.88401 1.228815 0.303878
H 4.155949 0.442683 -1.02475	H 4.154609 0.431417 -1.02671	H 4.122158 0.462154 -1.0835
H 3.713581 2.152537 -0.87309	H 3.704763 2.140681 -0.89487	H 3.729618 2.165205 -0.86432
H 4.802662 1.409819 0.294549	H 4.797826 1.41771 0.280475	H 4.817123 1.3619 0.267051
H 2.489294 2.989043 1.119603	H 2.463473 2.990608 1.110063	H 2.461752 2.999556 1.067623
H 1.701127 1.907133 2.264229	H 1.748979 1.890068 2.30262	H 1.744413 1.910007 2.272141
H 3.475335 2.080561 2.266145	H 3.499526 2.097873 2.235973	H 3.498559 2.128275 2.207625
H 2.120834 0.177918 -2.55894	H 2.128678 0.176951 -2.55463	H 1.912345 -1.26749 -2.69117
H 2.325912 0.044589 3.14802	H 1.32003 -0.57553 2.349889	H 2.354606 -1.2359 3.039404
H 3.63623 -1.56168 -1.21057	H 3.638842 -1.56776 -1.2034	H 3.633085 -1.49826 -1.14946
H -4.43857 -2.91679 -0.58286	H -4.45171 -2.90267 -0.60003	H -4.44552 -2.89881 -0.59774
H -3.66912 -2.97916 1.031345	H -3.68165 -2.98981 1.012869	H -3.67049 -2.98138 1.012724
H -5.44951 -2.98857 0.887089	H -5.46183 -2.98422 0.870128	H -5.45125 -2.97955 0.87526

2α10α17 (2.57%)

C 2.821348 -1.72873 0.626719

C 3.029133 -0.40243 1.377941

C 2.721045 0.891384 0.555836

C 1.344526 0.715282 -0.16531

C 1.203173 -0.60287 -0.99531

C 1.449246 -1.79626 -0.05278

C 0.873422 1.93978 -0.91445

C -0.42961 2.21243 -1.1063

C -1.51481 1.300045 -0.71113

C -1.37927 -0.09502 -0.88098

C -0.20254 -0.6628 -1.64593

C -2.70817 1.799486 -0.16036

C -3.74347 0.977247 0.271326

C -3.57324 -0.41577 0.130175

C -2.41374 -0.93642 -0.44875

C -5.01067 1.529554 0.872986

C 3.908322 1.213546 -0.38061

C 2.607748 2.07643 1.544859

H 0.623204 0.568942 0.651439

O 2.100084 -0.67043 -2.10935

O 2.170826 -0.54675 2.515609

O 3.895158 -1.87694 -0.3302

O -4.61852 -1.17917 0.577643

C -4.52517 -2.5883 0.460611

H 2.904464 -2.5231 1.379534

H 4.080946 -0.36372 1.702946

H 0.678916 -1.8212 0.72617

H 1.366587 -2.73158 -0.62464

H 1.613603 2.650138 -1.27454

H -0.71587 3.159596 -1.56361

H -0.09894 -0.10105 -2.58143

H -0.40461 -1.70357 -1.92592

H -2.82205 2.876878 -0.0536

H -2.30787 -2.00604 -0.59574

H -5.16088 1.163815 1.895979

H -4.98365 2.623249 0.899915

H -5.89341 1.220491 0.300441

H 4.153971 0.408457 -1.0693

H 3.702376 2.109478 -0.97498

H 4.804384 1.420232 0.218331

H 2.532151 3.024943 1.004367

H 1.718523 2.003153 2.181006

H 3.498575 2.140589 2.185665

H 2.939404 -1.03087 -1.7702

H 2.318977 0.212957 3.097042

H 3.888026 -2.80063 -0.6276

H -4.44331 -2.90314 -0.58837

H -3.66833 -2.98483 1.02161

H −5. 44922 −2. 9865 0. 884605

Table S9. Conformers and Boltzmann populations of 2β,10α-OH isomer of **6**.

**2β10α1** (20.69%)	**2β10α2** (26.62%)	**2β10α3** (29.01%)
C 3. 188918 1. 524535 0. 644379	C 3. 176744 1. 535417 0. 63175	C 3. 185267 1. 555423 0. 637164
C 3. 912973 0. 264903 0. 161706	C 3. 903277 0. 26186 0. 173736	C 3. 901848 0. 278277 0. 188516
C 3. 039611 −1. 01239 0. 221838	C 3. 030907 −1. 01658 0. 227927	C 3. 024975 −1. 00248 0. 241223
C 1. 677732 −0. 73931 −0. 52915	C 1. 676556 −0. 74054 −0. 53487	C 1. 67426 −0. 73209 −0. 52848
C 0. 944596 0. 55841 −0. 07908	C 0. 944976 0. 559631 −0. 08957	C 0. 941114 0. 570092 −0. 089
C 1. 906054 1. 753262 −0. 15194	C 1. 900463 1. 757848 −0. 16744	C 1. 901337 1. 76515 −0. 17117
C 0. 801932 −1. 97127 −0. 56507	C 0. 800005 −1. 97175 −0. 57857	C 0. 801465 −1. 9663 −0. 57522
C −0. 53274 −2. 14589 −0. 48828	C −0. 53465 −2. 14629 −0. 50158	C −0. 53298 −2. 14331 −0. 50176
C −1. 65265 −1. 20561 −0. 34692	C −1. 65336 −1. 20574 −0. 3539	C −1. 65374 −1. 20526 −0. 3564
C −1. 56516 0. 182992 −0. 57085	C −1. 56489 0. 182964 −0. 57697	C −1. 5676 0. 183642 −0. 58012
C −0. 2761 0. 839646 −1. 00684	C −0. 27647 0. 837721 −1. 01703	C −0. 28003 0. 841225 −1. 01868
C −2. 9084 −1. 73963 0. 013394	C −2. 90836 −1. 73886 0. 010366	C −2. 90795 −1. 74138 0. 00631
C −4. 05213 −0. 96948 0. 176591	C −4. 05027 −0. 96721 0. 179123	C −4. 05147 −0. 97212 0. 174222
C −3. 93428 0. 419412 −0. 03499	C −3. 93129 0. 421925 −0. 03092	C −3. 93491 0. 417457 −0. 03526
C −2. 70673 0. 976175 −0. 39978	C −2. 70458 0. 977562 −0. 40021	C −2. 7091 0. 97568 −0. 40395
C −5. 37897 −1. 57021 0. 56447	C −5. 3765 −1. 56646 0. 571357	C −5. 37682 −1. 57383 0. 565579
C 2. 838655 −1. 43581 1. 692962	C 2. 817822 −1. 43575 1. 698572	C 2. 81165 −1. 41609 1. 713301
C 3. 833462 −2. 13369 −0. 48931	C 3. 829461 −2. 1401 −0. 47396	C 3. 821471 −2. 13359 −0. 45269
H 1. 986565 −0. 54464 −1. 56764	H 1. 995082 −0. 54672 −1. 57068	H 1. 995823 −0. 53743 −1. 564
O 0. 521953 0. 516075 1. 289992	O 0. 523237 0. 518823 1. 28044	O 0. 518078 0. 530958 1. 279818
O 4. 341986 0. 472271 −1. 18417	O 4. 361383 0. 464388 −1. 16061	O 4. 35453 0. 572934 −1. 1504
O 4. 129435 2. 594173 0. 439491	O 4. 028104 2. 671642 0. 377028	O 4. 063512 2. 668883 0. 52443
O −5. 08372 1. 138075 0. 138702	O −5. 07886 1. 141912 0. 148729	O −5. 08354 1. 134953 0. 14412
C −5. 04207 2. 542657 −0. 05501	C −5. 03548 2. 547051 −0. 04203	C −5. 04196 2. 541218 −0. 04123
H 2. 956721 1. 435536 1. 712004	H 2. 939322 1. 477824 1. 699804	H 2. 935057 1. 474948 1. 697659
H 4. 793863 0. 107683 0. 806608	H 4. 773621 0. 098873 0. 838504	H 4. 779496 0. 129623 0. 835911
H 2. 173239 1. 940965 −1. 19765	H 2. 167912 1. 941768 −1. 21386	H 2. 164141 1. 945095 −1. 2208
H 1. 379728 2. 638904 0. 229336	H 1. 385787 2. 648175 0. 208584	H 1. 388099 2. 657816 0. 201729
H 1. 362574 −2. 89397 −0. 69078	H 1. 360102 −2. 89395 −0. 7112	H 1. 362373 −2. 8887 −0. 70594
H −0. 86159 −3. 18483 −0. 52452	H −0. 86419 −3. 18483 −0. 54393	H −0. 86019 −3. 18259 −0. 54456
H −0. 02374 0. 524638 −2. 02836	H −0. 02528 0. 518004 −2. 03742	H −0. 02682 0. 520713 −2. 03847
H −0. 43913 1. 922666 −1. 04582	H −0. 43821 1. 92053 −1. 05998	H −0. 44405 1. 92345 −1. 06331
H −2. 98416 −2. 81335 0. 174834	H −2. 98499 −2. 81274 0. 170493	H −2. 98247 −2. 81539 0. 166594
H −2. 62073 2. 044299 −0. 56626	H −2. 61746 2. 045726 −0. 56556	H −2. 62393 2. 044032 −0. 5688

H -6.14569 -1.36683 -0.19259
H -5.29478 -2.65433 0.686453
H -5.75029 -1.14528 1.504674
H 2.331209 -0.67301 2.28503
H 2.232914 -2.34782 1.743254
H 3.807276 -1.65852 2.157438
H 3.37862 -3.11681 -0.33128
H 3.92034 -1.95205 -1.56359
H 4.849775 -2.18123 -0.08049
H -0.11396 -0.21281 1.380281
H 4.709225 1.373943 -1.19019
H 3.663886 3.43289 0.580024
H -6.05496 2.901266 0.137695
H -4.34673 3.025789 0.643912
H -4.75574 2.799924 -1.08331

H -6.14595 -1.36062 -0.18228
H -5.2936 -2.65093 0.691251
H -5.7434 -1.14246 1.513726
H 2.314616 -0.66665 2.286251
H 2.201957 -2.34092 1.747135
H 3.781623 -1.66918 2.168444
H 3.372274 -3.12238 -0.31735
H 3.924499 -1.9609 -1.54804
H 4.842756 -2.18826 -0.05733
H -0.1232 -0.20098 1.368971
H 4.638253 1.399144 -1.1843
H 4.737626 2.660853 1.039593
H -6.04685 2.906977 0.156078
H -4.33604 3.027233 0.654734
H -4.75337 2.806027 -1.07103

H -6.14607 -1.36975 -0.18874
H -5.29189 -2.65809 0.686021
H -5.74522 -1.15006 1.507429
H 2.305251 -0.64316 2.291989
H 2.198652 -2.323 1.766976
H 3.775312 -1.64098 2.188072
H 3.37948 -3.11766 -0.27145
H 3.883616 -1.99057 -1.53738
H 4.843477 -2.17524 -0.05122
H -0.1453 -0.17362 1.365192
H 5.076496 -0.03493 -1.36468
H 4.474186 2.57084 -0.35257
H -6.0538 2.898834 0.158447
H -4.343 3.019269 0.657313
H -4.76043 2.804327 -1.06929

2β10α4 (20.69%)

C 3.188844 1.524588 0.644413
C 3.912954 0.264903 0.161878
C 3.039553 -1.01238 0.221933
C 1.677754 -0.73928 -0.52925
C 0.944601 0.558407 -0.07928
C 1.906038 1.75327 -0.15203
C 0.80196 -1.97123 -0.56527
C -0.53271 -2.14588 -0.48836
C -1.65262 -1.2056 -0.347
C -1.56514 0.182972 -0.57097
C -0.27609 0.839557 -1.00708
C -2.90837 -1.73964 0.013367
C -4.05207 -0.96947 0.176603
C -3.93423 0.419439 -0.03495
C -2.7067 0.976168 -0.39985
C -5.37889 -1.57017 0.564613
C 2.838423 -1.43581 1.693009
C 3.833414 -2.13371 -0.48912
H 1.986776 -0.54464 -1.56768
O 0.521846 0.51611 1.289791
O 4.342216 0.472227 -1.18391

2β10α6 (1.50%)

C 3.186204 1.554034 0.644
C 3.910438 0.275615 0.1961
C 3.032907 -1.00567 0.228523
C 1.672917 -0.73414 -0.53076
C 0.939669 0.569919 -0.09075
C 1.902264 1.764505 -0.16747
C 0.800017 -1.96933 -0.57048
C -0.53469 -2.14235 -0.49494
C -1.65523 -1.20365 -0.35309
C -1.56995 0.184927 -0.57866
C -0.28281 0.843002 -1.01834
C -2.90935 -1.73979 0.010256
C -4.05309 -0.97079 0.176852
C -3.93719 0.418686 -0.03464
C -2.71141 0.977 -0.40411
C -5.37817 -1.5724 0.569046
C 2.81181 -1.4262 1.698502
C 3.829696 -2.13334 -0.4688
H 1.967205 -0.55173 -1.58107
O 0.521192 0.527966 1.276867
O 4.506308 0.524436 -1.09566

2β10α9 (1.51%)

C 3.186025 1.55408 0.644103
C 3.910376 0.275641 0.196414
C 3.032762 -1.00564 0.228594
C 1.672965 -0.73406 -0.53103
C 0.939675 0.569985 -0.09102
C 1.902232 1.764544 -0.16759
C 0.800069 -1.96923 -0.57119
C -0.53462 -2.14228 -0.49536
C -1.65511 -1.20361 -0.35323
C -1.56992 0.184949 -0.57881
C -0.28283 0.843021 -1.01859
C -2.9092 -1.73981 0.010268
C -4.05294 -0.97087 0.177029
C -3.93709 0.418669 -0.03446
C -2.7114 0.976999 -0.4041
C -5.37791 -1.57239 0.569599
C 2.811333 -1.42625 1.698524
C 3.829706 -2.13336 -0.46851
H 1.967564 -0.55152 -1.58123
O 0.521182 0.527965 1.276651
O 4.506745 0.524362 -1.0951

O	4.129374	2.59422	0.439505	O	4.061935	2.667411	0.525153	O	4.061671	2.667491	0.525336
O	-5.08366	1.138077	0.138907	O	-5.08545	1.135503	0.14347	O	-5.08532	1.135435	0.144
C	-5.04213	2.542627	-0.05498	C	-5.04521	2.542174	-0.04195	C	-5.04547	2.541966	-0.04252
H	2.956582	1.435665	1.712023	H	2.925352	1.477229	1.702743	H	2.925035	1.477285	1.702827
H	4.793727	0.107686	0.806947	H	4.777179	0.120558	0.847879	H	4.776839	0.120574	0.848562
H	2.173323	1.941039	-1.19771	H	2.158788	1.959235	-1.21924	H	2.159006	1.959303	-1.21928
H	1.379648	2.638902	0.229179	H	1.389948	2.659322	0.200347	H	1.389899	2.659401	0.200132
H	1.362606	-2.89391	-0.69113	H	1.362052	-2.89123	-0.69492	H	1.362064	-2.89108	-0.69614
H	-0.86153	-3.18482	-0.52466	H	-0.86215	-3.18172	-0.53024	H	-0.86208	-3.18164	-0.53095
H	-0.02378	0.524381	-2.02856	H	-0.03347	0.524556	-2.04002	H	-0.03354	0.524405	-2.04024
H	-0.4391	1.922574	-1.04626	H	-0.44647	1.925362	-1.06038	H	-0.44648	1.925372	-1.0608
H	-2.98416	-2.81335	0.174766	H	-2.98341	-2.81345	0.172452	H	-2.98316	-2.8135	0.17234
H	-2.62067	2.044282	-0.56638	H	-2.62699	2.04515	-0.57052	H	-2.62702	2.045163	-0.57046
H	-6.14611	-1.3653	-0.19152	H	-6.14699	-1.37049	-0.18622	H	-6.14786	-1.36708	-0.18355
H	-5.2951	-2.65452	0.684917	H	-5.29265	-2.65625	0.692174	H	-5.29337	-2.65673	0.689062
H	-5.74927	-1.14657	1.505803	H	-5.74704	-1.14636	1.509611	H	-5.74459	-1.14912	1.512312
H	2.331059	-0.67295	2.285076	H	2.31765	-0.65121	2.28506	H	2.317074	-0.65125	2.284995
H	2.232597	-2.34776	1.743233	H	2.187085	-2.32526	1.746035	H	2.18658	-2.32529	1.745884
H	3.806986	-1.65862	2.15757	H	3.773658	-1.66752	2.167428	H	3.773087	-1.66758	2.167608
H	3.37854	-3.11682	-0.3311	H	3.377224	-3.11524	-0.29916	H	3.377274	-3.11525	-0.29872
H	3.920345	-1.95211	-1.56341	H	3.91801	-1.9769	-1.54845	H	3.918015	-1.97713	-1.5482
H	4.849706	-2.18127	-0.08025	H	4.847055	-2.17175	-0.06331	H	4.847055	-2.17162	-0.06301
H	-0.11352	-0.21323	1.380178	H	-0.14229	-0.17639	1.364026	H	-0.14277	-0.17597	1.363592
H	4.709552	1.373865	-1.18992	H	3.79921	0.524536	-1.76041	H	3.800026	0.523931	-1.76024
H	3.663845	3.432942	0.580112	H	4.564603	2.49955	-0.2927	H	4.564652	2.499479	-0.29228
H	-6.05502	2.901195	0.13779	H	-6.0573	2.898553	0.158208	H	-6.05767	2.898259	0.157197
H	-4.34679	3.025908	0.643834	H	-4.3465	3.020531	0.656503	H	-4.34702	3.021081	0.655673
H	-4.75598	2.799802	-1.08335	H	-4.76461	2.805187	-1.07022	H	-4.76477	2.804244	-1.07095

Table S10. Conformers and Boltzmann populations of 2α,10β-OH isomer of **6**.

**2α10β1** (28.98%)				**2α10β2** (23.10%)				**2α10β3** (15.17%)			
C	3.479124	-1.19501	-0.28603	C	3.482507	-1.22152	-0.29927	C	3.486109	-1.20517	-0.3245
C	3.655695	0.300564	-0.0243	C	3.650964	0.274401	-0.03216	C	3.653679	0.287416	-0.01751
C	2.512832	0.898151	0.827743	C	2.514883	0.879696	0.830686	C	2.505862	0.887017	0.834274
C	1.137269	0.585178	0.128397	C	1.137456	0.577145	0.132019	C	1.135005	0.573259	0.124874
C	0.946928	-0.92152	-0.27918	C	0.944501	-0.93113	-0.27241	C	0.952424	-0.93333	-0.28314
C	2.172088	-1.46425	-1.02855	C	2.16479	-1.48423	-1.01936	C	2.170315	-1.46506	-1.04836
C	0.80218	1.528888	-1.01496	C	0.808419	1.523484	-1.01055	C	0.810634	1.513913	-1.02308

C	-0.45754	1.780027	-1.41437	C	-0.45027	1.779631	-1.41025	C	-0.44799	1.765833	-1.4253
C	-1.62725	1.049693	-0.90085	C	-1.62223	1.052117	-0.89814	C	-1.61979	1.042523	-0.90592
C	-1.55262	-0.34528	-0.70425	C	-1.55166	-0.34357	-0.70317	C	-1.5501	-0.35184	-0.70087
C	-0.32286	-1.10355	-1.15415	C	-0.32377	-1.10599	-1.15141	C	-0.3243	-1.11866	-1.1479
C	-2.83585	1.707159	-0.61302	C	-2.82912	1.713241	-0.61135	C	-2.82564	1.706479	-0.62157
C	-3.94482	1.049266	-0.09108	C	-3.94065	1.05843	-0.09113	C	-3.93703	1.056161	-0.09527
C	-3.83676	-0.33923	0.138022	C	-3.83703	-0.33067	0.136816	C	-3.83451	-0.33149	0.141585
C	-2.65981	-1.02415	-0.17627	C	-2.66181	-1.01902	-0.17708	C	-2.66017	-1.0229	-0.16898
C	-5.22911	1.771144	0.228448	C	-5.22329	1.783763	0.227153	C	-5.21855	1.784809	0.219833
C	2.732752	2.422377	0.958592	C	2.73604	2.404233	0.967992	C	2.719269	2.410311	0.976531
C	2.540165	0.3293	2.266511	C	2.544031	0.304476	2.266025	C	2.510053	0.309817	2.271529
H	0.36143	0.762931	0.889264	H	0.362539	0.758853	0.892826	H	0.352456	0.752065	0.878323
O	0.814009	-1.74828	0.888993	O	0.796688	-1.74712	0.90069	O	0.830712	-1.75462	0.890416
O	4.904454	0.543379	0.608949	O	4.950663	0.416332	0.564033	O	4.960774	0.511667	0.536702
O	4.623145	-1.57642	-1.07201	O	4.537177	-1.68519	-1.13617	O	4.549942	-1.64164	-1.16282
O	-4.9538	-0.93192	0.659276	O	-4.95606	-0.92023	0.656181	O	-4.95357	-0.91669	0.66587
C	-4.9291	-2.32855	0.903704	C	-4.93669	-2.31782	0.897887	C	-4.93691	-2.31331	0.912673
H	3.497121	-1.73282	0.669374	H	3.501039	-1.75912	0.659124	H	3.501327	-1.77066	0.6217
H	3.645524	0.79626	-1.01311	H	3.65577	0.77384	-1.01554	H	3.6907	0.811863	-0.98093
H	2.235484	-0.98566	-2.01324	H	2.223613	-1.01613	-2.00979	H	2.222923	-0.9733	-2.02745
H	2.038157	-2.54119	-1.1942	H	2.037924	-2.56119	-1.17462	H	2.049702	-2.53906	-1.22823
H	1.612638	2.075801	-1.48816	H	1.620621	2.065438	-1.48746	H	1.624312	2.050825	-1.5028
H	-0.63668	2.559045	-2.15542	H	-0.62621	2.55848	-2.15227	H	-0.62425	2.53802	-2.17404
H	-0.08294	-0.78699	-2.17546	H	-0.07979	-0.78948	-2.17156	H	-0.08859	-0.81385	-2.17354
H	-0.53194	-2.17859	-1.18515	H	-0.53648	-2.18006	-1.18492	H	-0.536	-2.19331	-1.16846
H	-2.90312	2.778958	-0.79019	H	-2.89324	2.785313	-0.78831	H	-2.88904	2.777348	-0.80571
H	-2.59715	-2.09916	-0.04372	H	-2.60314	-2.09451	-0.04678	H	-2.60287	-2.09772	-0.03235
H	-5.14861	2.834592	-0.01616	H	-5.1395	2.847179	-0.01662	H	-5.134	2.846534	-0.0308
H	-6.07337	1.350987	-0.33101	H	-6.06789	1.3664	-0.33385	H	-6.06415	1.364674	-0.33757
H	-5.48505	1.680543	1.290923	H	-5.4811	1.693058	1.289166	H	-5.47526	1.701114	1.28269
H	1.876686	2.895896	1.454038	H	1.87089	2.882451	1.441193	H	1.876435	2.872664	1.503875
H	3.626927	2.613317	1.558646	H	3.604645	2.603574	1.607267	H	3.631225	2.606009	1.548863
H	2.878981	2.917187	-0.00644	H	2.90523	2.905062	0.008405	H	2.83053	2.916305	0.013312
H	3.524613	0.50011	2.710691	H	3.538043	0.445047	2.701493	H	3.44729	0.555303	2.788364
H	1.795093	0.848706	2.882729	H	1.820235	0.838953	2.894574	H	1.708305	0.775469	2.857446
H	2.315792	-0.73698	2.302838	H	2.293552	-0.75547	2.295655	H	2.355156	-0.7688	2.298472
H	0.047758	-1.41593	1.386361	H	0.006776	-1.42881	1.369811	H	0.032644	-1.4588	1.360523
H	5.538544	-0.01574	0.127419	H	5.223649	1.341653	0.479325	H	4.97484	0.10203	1.417479

H 4.690481 -2.54289 -1.05329
H -5.90673 -2.57688 1.321133
H -4.77484 -2.89838 -0.02212
H -4.14718 -2.59847 1.625934

2α10β4 (17.23%)

C 3.478164 -1.20117 -0.28657
C 3.654699 0.297045 -0.01901
C 2.513273 0.897201 0.833645
C 1.138685 0.585179 0.131322
C 0.947661 -0.92206 -0.27663
C 2.171625 -1.47208 -1.02585
C 0.803812 1.530903 -1.01054
C -0.45585 1.781699 -1.4109
C -1.62584 1.050395 -0.89995
C -1.55138 -0.34453 -0.70311
C -0.32179 -1.10314 -1.15255
C -2.8349 1.7079 -0.61371
C -3.94422 1.050009 -0.09276
C -3.83626 -0.33857 0.136869
C -2.65897 -1.02349 -0.17627
C -5.22892 1.771691 0.225367
C 2.734137 2.420674 0.968891
C 2.541722 0.322813 2.269952
H 0.361731 0.761543 0.891266
O 0.814967 -1.74725 0.890683
O 4.907945 0.535684 0.60294
O 4.624294 -1.69368 -1.00188
O -4.95338 -0.93097 0.657133
C -4.92904 -2.32774 0.902457
H 3.499987 -1.74707 0.659383
H 3.637096 0.801121 -1.00821
H 2.217847 -0.99046 -2.01379
H 2.046579 -2.54826 -1.18858
H 1.613417 2.082764 -1.47974
H -0.63477 2.563419 -2.14919
H -0.08214 -0.78601 -2.17389
H -0.53077 -2.17806 -1.18414

H 5.348445 -1.32995 -0.73412
H -5.91569 -2.56314 1.313809
H -4.78362 -2.88607 -0.02897
H -4.15653 -2.59181 1.620365

2α10β5 (3.84%)

C 3.489638 -1.19881 -0.33347
C 3.650434 0.291948 -0.03478
C 2.515537 0.872332 0.847454
C 1.132734 0.561319 0.15918
C 0.952092 -0.93198 -0.269
C 2.170892 -1.4463 -1.05659
C 0.801967 1.512215 -0.98133
C -0.45304 1.76999 -1.38948
C -1.63025 1.044771 -0.88903
C -1.5601 -0.34745 -0.67995
C -0.33223 -1.11309 -1.12285
C -2.84015 1.70901 -0.62264
C -3.95696 1.058231 -0.11009
C -3.85263 -0.32808 0.133415
C -2.6743 -1.01786 -0.16119
C -5.2438 1.784807 0.187772
C 2.718479 2.399287 0.994695
C 2.569474 0.290778 2.27926
H 0.360172 0.714047 0.924965
O 0.797676 -1.67709 0.952649
O 4.954632 0.425674 0.552712
O 4.546298 -1.64604 -1.17547
O -4.97676 -0.91401 0.648265
C -4.94835 -2.30344 0.92891
H 3.507462 -1.75286 0.616292
H 3.646292 0.811484 -1.00775
H 2.218752 -0.94596 -2.03186
H 2.060615 -2.52 -1.26501
H 1.615422 2.058574 -1.45079
H -0.62104 2.552412 -2.12984
H -0.09945 -0.81742 -2.15245
H -0.55646 -2.18794 -1.15082

H 5.341823 -1.19237 -0.81774
H -5.91628 -2.55524 1.329679
H -4.78509 -2.88529 -0.01211
H -4.15721 -2.5863 1.636108

2α10β7 (2.90%)

C 3.492783 -1.18047 -0.36069
C 3.652493 0.306179 -0.0198
C 2.505462 0.878476 0.852092
C 1.129963 0.556275 0.152468
C 0.960517 -0.93495 -0.28106
C 2.176274 -1.42493 -1.08824
C 0.804672 1.50195 -0.99328
C -0.4501 1.756158 -1.404
C -1.62736 1.035239 -0.89654
C -1.55833 -0.35563 -0.67762
C -0.33268 -1.12588 -1.11958
C -2.83622 1.702291 -0.63269
C -3.95305 1.056025 -0.11434
C -3.85 -0.32875 0.138053
C -2.67255 -1.0216 -0.15311
C -5.23878 1.78593 0.180127
C 2.698718 2.403814 1.008126
C 2.535412 0.291013 2.284698
H 0.3494 0.70629 0.910419
O 0.835475 -1.68905 0.939266
O 4.962264 0.523017 0.530323
O 4.559896 -1.598 -1.20292
O -4.97427 -0.91025 0.657542
C -4.94812 -2.29836 0.944538
H 3.506733 -1.76377 0.575215
H 3.68118 0.851411 -0.97188
H 2.216781 -0.90017 -2.0508
H 2.072121 -2.49416 -1.32173
H 1.620003 2.042903 -1.46538
H -0.61802 2.532254 -2.15084
H -0.1087 -0.84202 -2.15443
H -0.55665 -2.20112 -1.13462

H -2.90208 2.779668 -0.79104
H -2.5963 -2.09844 -0.0435
H -5.14837 2.835177 -0.01902
H -6.07243 1.351373 -0.33507
H -5.48596 1.680836 1.287529
H 1.879945 2.893167 1.468557
H 3.629966 2.608921 1.567542
H 2.87776 2.919281 0.00529
H 3.525503 0.495464 2.714753
H 1.794635 0.837137 2.887872
H 2.321514 -0.7444 2.3022
H 0.049564 -1.41455 1.389058
H 5.513492 -0.11095 0.197925
H 4.582579 -1.32781 -1.9019
H -5.90715 -2.57553 1.318983
H -4.77396 -2.89792 -0.02294
H -4.14794 -2.5971 1.625684

2α10β8 (2.59%)

C 3.490859 -1.17363 -0.32114
C 3.655656 0.318178 -0.02857
C 2.515542 0.887521 0.846832
C 1.134545 0.56799 0.159072
C 0.953472 -0.92573 -0.26655
C 2.177004 -1.43617 -1.05448
C 0.800033 1.513765 -0.9849
C -0.45559 1.767201 -1.39374
C -1.63185 1.041514 -0.89093
C -1.56027 -0.34984 -0.67852
C -0.32986 -1.11255 -1.1194
C -2.84299 1.703867 -0.62588
C -3.95918 1.052087 -0.11295
C -3.85344 -0.33365 0.132353
C -2.67375 -1.02193 -0.16014
C -5.24697 1.777569 0.183549
C 2.715325 2.41373 0.993044
C 2.570989 0.30582 2.279281
H 0.361718 0.72033 0.92429

H -2.90153 2.779635 -0.8093
H -2.61174 -2.0892 -0.00463
H -5.15854 2.846069 -0.06511
H -6.08251 1.36159 -0.37783
H -5.51226 1.704009 1.247963
H 1.850581 2.861182 1.478533
H 3.588491 2.604759 1.630399
H 2.874668 2.911498 0.039055
H 3.566244 0.445343 2.703973
H 1.842521 0.809379 2.915992
H 2.33005 -0.77084 2.309591
H 0.768826 -2.61737 0.712083
H 5.212394 1.358248 0.508944
H 5.354051 -1.27848 -0.77685
H -5.92885 -2.54537 1.343663
H -4.784 -2.8966 0.019375
H -4.17153 -2.5506 1.664451

2α10β11 (3.85%)

C 3.489769 -1.19851 -0.33389
C 3.65047 0.292224 -0.03453
C 2.515291 0.872281 0.847647
C 1.132656 0.561241 0.159166
C 0.952148 -0.93206 -0.26911
C 2.170971 -1.44606 -1.05685
C 0.801988 1.512158 -0.98137
C -0.45304 1.770017 -1.38945
C -1.63024 1.04481 -0.88905
C -1.56003 -0.34746 -0.68007
C -0.33222 -1.11308 -1.12302
C -2.84018 1.708975 -0.62271
C -3.95702 1.058184 -0.11022
C -3.85262 -0.32814 0.133239
C -2.67427 -1.01787 -0.1614
C -5.24388 1.784675 0.187592
C 2.718004 2.3992 0.995281
C 2.569193 0.290437 2.279299
H 0.359934 0.713868 0.924809

H -2.89676 2.771665 -0.82643
H -2.61132 -2.09213 0.009727
H -5.15277 2.845318 -0.08013
H -6.07853 1.359621 -0.38156
H -5.50602 1.712677 1.241174
H 1.856054 2.846582 1.551888
H 3.614702 2.606172 1.571786
H 2.79018 2.923625 0.050311
H 3.470073 0.558627 2.795996
H 1.723949 0.727906 2.877554
H 2.404457 -0.79017 2.308066
H 0.787332 -2.6267 0.691542
H 4.972502 0.124838 1.416339
H 5.347165 -1.14579 -0.85037
H -5.9287 -2.53663 1.361193
H -4.78558 -2.896 0.037614
H -4.1712 -2.54364 1.680662

2α10β14 (2.34%)

C 3.487442 -1.17882 -0.32186
C 3.656332 0.314128 -0.02128
C 2.515451 0.888182 0.851355
C 1.13521 0.569063 0.160118
C 0.954573 -0.92439 -0.26866
C 2.177262 -1.43597 -1.05934
C 0.799687 1.517677 -0.98128
C -0.45616 1.770219 -1.39057
C -1.63202 1.042482 -0.89045
C -1.55957 -0.34892 -0.67868
C -0.32953 -1.1113 -1.12077
C -2.84376 1.704025 -0.62569
C -3.95941 1.051352 -0.11299
C -3.8526 -0.33446 0.132354
C -2.67247 -1.02193 -0.16047
C -5.24778 1.775689 0.183593
C 2.717439 2.413764 0.999277
C 2.569418 0.304326 2.282818
H 0.361498 0.718509 0.925008

O	0.808414	-1.67393	0.954444	O	0.797788	-1.6773	0.952389	O	0.813862	-1.67426	0.949625
O	4.90925	0.557903	0.595847	O	4.954422	0.425428	0.553638	O	4.912369	0.545778	0.597524
O	4.629887	-1.52917	-1.12594	O	4.546215	-1.64513	-1.17634	O	4.631141	-1.65479	-1.05154
O	-4.97759	-0.9207	0.646768	O	-4.97671	-0.91421	0.647955	O	-4.97593	-0.92215	0.646752
C	-4.94713	-2.30909	0.930317	C	-4.94764	-2.30336	0.929781	C	-4.94406	-2.31035	0.932525
H	3.522703	-1.72891	0.624363	H	3.507925	-1.75278	0.615748	H	3.514821	-1.74229	0.613976
H	3.631718	0.832851	-1.00727	H	3.646844	0.812154	-1.00725	H	3.630468	0.838507	-0.99963
H	2.225017	-0.93485	-2.02906	H	2.218646	-0.94552	-2.03203	H	2.210534	-0.92301	-2.03217
H	2.059891	-2.51176	-1.261	H	2.060939	-2.51974	-1.26544	H	2.069625	-2.50868	-1.27321
H	1.612407	2.063309	-1.45159	H	1.615466	2.058405	-1.45092	H	1.610648	2.073161	-1.44366
H	-0.62586	2.548243	-2.13497	H	-0.62106	2.552492	-2.12975	H	-0.62694	2.554477	-2.12833
H	-0.09726	-0.81679	-2.14901	H	-0.09939	-0.81733	-2.15259	H	-0.09886	-0.81508	-2.15087
H	-0.5522	-2.18865	-1.14899	H	-0.55651	-2.18791	-1.15108	H	-0.55097	-2.18723	-1.14952
H	-2.90571	2.774126	-0.81379	H	-2.90153	2.779617	-0.8093	H	-2.90719	2.774306	-0.81321
H	-2.61053	-2.09323	-0.00263	H	-2.6118	-2.08924	-0.00496	H	-2.60824	-2.09313	-0.00308
H	-5.16257	2.838643	-0.07021	H	-5.15873	2.84594	-0.06529	H	-5.16451	2.836801	-0.07036
H	-6.08499	1.352947	-0.38204	H	-6.08256	1.361387	-0.37801	H	-6.08545	1.350067	-0.38173
H	-5.51569	1.697514	1.243715	H	-5.51237	1.703875	1.247786	H	-5.51615	1.69548	1.243821
H	1.858846	2.867083	1.50593	H	1.850154	2.860763	1.479553	H	1.862208	2.868147	1.513331
H	3.614038	2.609987	1.584557	H	3.58813	2.604613	1.630869	H	3.616605	2.607591	1.591162
H	2.84159	2.924142	0.033346	H	2.873868	2.911715	0.039786	H	2.844074	2.925793	0.040362
H	3.556668	0.494998	2.713436	H	3.565882	0.445058	2.704181	H	3.554022	0.494695	2.718666
H	1.819382	0.800775	2.906065	H	1.842039	0.808676	2.916099	H	1.81572	0.796906	2.90885
H	2.364771	-0.76347	2.311362	H	2.329999	-0.77125	2.309417	H	2.365256	-0.76532	2.312724
H	0.66819	-2.60201	0.706	H	0.768949	-2.61755	0.711699	H	0.714332	-2.60826	0.703318
H	5.545624	0.025771	0.087797	H	5.212872	1.357783	0.509243	H	5.521153	-0.08445	0.172515
H	4.717265	-2.49404	-1.11701	H	5.354196	-1.27905	-0.77686	H	4.592076	-1.25966	-1.93923
H	-5.92764	-2.55209	1.344458	H	-5.92782	-2.5453	1.345287	H	-5.92407	-2.55334	1.347783
H	-4.78071	-2.90418	0.022286	H	-4.7836	-2.89728	0.020678	H	-4.77796	-2.90655	0.025231
H	-4.1708	-2.55354	1.667424	H	-4.17031	-2.54962	1.665097	H	-4.16695	-2.55277	1.669361

Table S11. Conformers and Boltzmann populations of 2β,10β-OH isomer of **6**.

**2β10β1** (55.19%)				**2β10β2** (4.00%)				**2β10β3** (5.94%)			
C	3.538389	-1.05381	-0.78035	C	3.532959	-1.028	-0.81102	C	3.035186	1.621854	0.364075
C	3.737156	0.342326	-0.15577	C	3.704526	0.360306	-0.18065	C	3.806451	0.295728	0.409955
C	2.566948	0.853018	0.745243	C	2.555945	0.829494	0.762901	C	2.885599	-0.93833	0.564121
C	1.178772	0.611296	0.039975	C	1.166614	0.582172	0.06374	C	1.727746	-0.88085	-0.51931

C	1.004042	-0.81758	-0.5714	C	1.001032	-0.85646	-0.5468	C	0.98992	0.479233	-0.68674
C	2.184597	-1.18366	-1.47901	C	2.175457	-1.17411	-1.49124	C	2.022903	1.615891	-0.77585
C	0.790714	1.682342	-0.96645	C	0.788056	1.66181	-0.93618	C	0.817656	-2.07887	-0.41665
C	-0.48797	1.961467	-1.27709	C	-0.4865	1.948579	-1.25837	C	-0.51225	-2.20533	-0.23216
C	-1.62679	1.163418	-0.79551	C	-1.63245	1.154948	-0.79192	C	-1.59716	-1.2295	-0.06191
C	-1.53425	-0.24389	-0.75973	C	-1.53872	-0.25144	-0.73882	C	-1.42598	0.145731	0.199189
C	-0.32783	-0.93478	-1.35933	C	-0.33527	-0.9551	-1.32924	C	-0.0653	0.768353	0.419502
C	-2.824	1.771002	-0.37893	C	-2.83527	1.768987	-0.4004	C	-2.91734	-1.71894	-0.17766
C	-3.9029	1.04635	0.116541	C	-3.92004	1.050513	0.089635	C	-4.04787	-0.9221	-0.06641
C	-3.77606	-0.35801	0.181903	C	-3.7897	-0.35233	0.178463	C	-3.84931	0.453009	0.173384
C	-2.61107	-0.99008	-0.26151	C	-2.62075	-0.98928	-0.24345	C	-2.55785	0.966441	0.300562
C	-5.17487	1.713188	0.574583	C	-5.1986	1.721467	0.523013	C	-5.44235	-1.47853	-0.19829
C	2.797284	2.359005	1.01042	C	2.755479	2.33883	1.055829	C	2.38195	-1.00971	2.023407
C	2.585478	0.187639	2.143545	C	2.592973	0.134201	2.146361	C	3.747066	-2.2004	0.322051
H	0.416587	0.672057	0.8332	H	0.411968	0.618119	0.860401	H	2.259596	-0.99093	-1.47548
O	1.002349	-1.8216	0.473271	O	0.922638	-1.843	0.486743	O	0.357741	0.517554	-1.97244
O	4.981493	0.357163	0.53155	O	4.982619	0.298375	0.466873	O	4.570315	0.168256	-0.78473
O	3.762043	-2.07741	0.19535	O	3.669412	-2.06294	0.17401	O	4.040312	2.62953	0.16274
O	-4.86354	-1.01759	0.683169	O	-4.88155	-1.0069	0.680416	O	-4.9884	1.201853	0.268791
C	-4.82245	-2.43316	0.762209	C	-4.82285	-2.41798	0.80986	C	-4.8672	2.596122	0.500047
H	4.339163	-1.18693	-1.51774	H	4.334882	-1.14745	-1.55542	H	2.539424	1.796923	1.329776
H	3.842683	1.052624	-0.98869	H	3.757935	1.074884	-1.01912	H	4.485865	0.326126	1.279027
H	2.058828	-2.20662	-1.85405	H	2.071769	-2.19417	-1.88062	H	2.568389	1.491911	-1.71637
H	2.170418	-0.51249	-2.34647	H	2.147975	-0.49187	-2.34984	H	1.490047	2.575418	-0.82925
H	1.576118	2.288466	-1.40866	H	1.576914	2.26768	-1.37541	H	1.341809	-3.02296	-0.55248
H	-0.70741	2.818076	-1.91427	H	-0.69417	2.809407	-1.89443	H	-0.8797	-3.2316	-0.25443
H	-0.52653	-2.0046	-1.48501	H	-0.54579	-2.02317	-1.44155	H	-0.19926	1.85394	0.489346
H	-0.16351	-0.51682	-2.35912	H	-0.17099	-0.54582	-2.33305	H	0.31872	0.440337	1.390508
H	-2.90578	2.855054	-0.43093	H	-2.91581	2.852622	-0.4669	H	-3.05569	-2.78119	-0.36995
H	-2.53645	-2.07242	-0.25408	H	-2.53555	-2.06939	-0.20305	H	-2.40968	2.023638	0.491199
H	-5.11228	2.798259	0.44871	H	-5.13637	2.804983	0.382135	H	-5.41567	-2.55715	-0.38029
H	-6.04182	1.349396	0.010427	H	-6.05909	1.348641	-0.04544	H	-6.03218	-1.2961	0.707897
H	-5.38003	1.500114	1.630512	H	-5.41594	1.5237	1.579618	H	-5.98555	-1.00299	-1.02365
H	1.976384	2.785869	1.599296	H	1.883881	2.746349	1.579282	H	1.612688	-1.78112	2.129922
H	3.727609	2.487191	1.572032	H	3.620582	2.499077	1.714539	H	3.213106	-1.27011	2.689276
H	2.890732	2.946341	0.091967	H	2.91251	2.938659	0.1541	H	1.963544	-0.06887	2.392813
H	3.552574	0.365264	2.622434	H	3.587776	0.221327	2.596487	H	4.654058	-2.15569	0.936177
H	1.814246	0.642122	2.778374	H	1.881766	0.630202	2.81706	H	3.206887	-3.11015	0.603267

H 2.409713 -0.88662 2.12186
H 0.32528 -1.57141 1.123816
H 5.041682 -0.52695 0.939208
H 2.897111 -2.25238 0.610074
H -5.77938 -2.7368 1.190699
H -4.00802 -2.77774 1.413183
H -4.70783 -2.89003 -0.22955

2β10β6 (9.56%)

C 3.030227 1.645602 0.357773
C 3.794362 0.316652 0.417439
C 2.878313 -0.92768 0.564714
C 1.724943 -0.87576 -0.52165
C 0.988748 0.485215 -0.69919
C 2.017847 1.624734 -0.78914
C 0.817499 -2.07617 -0.41579
C -0.51172 -2.20264 -0.22691
C -1.59732 -1.22774 -0.05806
C -1.42785 0.150106 0.19183
C -0.06907 0.780197 0.402674
C -2.91665 -1.72159 -0.16501
C -4.04868 -0.9267 -0.05606
C -3.85234 0.450864 0.171732
C -2.56159 0.968408 0.290339
C -5.44235 -1.48725 -0.17874
C 2.375166 -1.00033 2.024062
C 3.742256 -2.18897 0.3257
H 2.258357 -0.98727 -1.47747
O 0.358514 0.504905 -1.98651
O 4.569332 0.293897 -0.7956
O 3.945775 2.725434 0.22707
O -4.9923 1.197801 0.264493
C -4.87332 2.594724 0.484156
H 2.522793 1.810502 1.314328
H 4.47944 0.351757 1.278689
H 2.561431 1.50375 -1.73258
H 1.497274 2.586811 -0.83901
H 1.340265 -3.0215 -0.55233

H 2.320064 -0.91654 2.108995
H 1.828103 -2.17748 0.629992
H 5.118021 1.120732 0.96138
H 4.399983 -1.77867 0.751757
H -4.0071 -2.7285 1.475633
H -4.69691 -2.90845 -0.16454
H -5.77873 -2.71875 1.24337

2β10β8 (4.84%)

C 3.529193 -1.01608 -0.81632
C 3.70141 0.368457 -0.1672
C 2.553092 0.831264 0.769541
C 1.165811 0.579803 0.066907
C 0.999657 -0.85678 -0.54788
C 2.172306 -1.16698 -1.49726
C 0.787258 1.659209 -0.93327
C -0.48659 1.9465 -1.25755
C -1.63371 1.153795 -0.79214
C -1.54097 -0.2525 -0.73858
C -0.33766 -0.9564 -1.3289
C -2.83645 1.76844 -0.40157
C -3.92201 1.050536 0.087734
C -3.79249 -0.35226 0.177097
C -2.62357 -0.98991 -0.24377
C -5.20042 1.722392 0.520243
C 2.760385 2.335987 1.069172
C 2.592053 0.131662 2.151947
H 0.410352 0.614651 0.862622
O 0.925589 -1.84934 0.47957
O 4.929425 0.363071 0.585104
O 3.683461 -2.05218 0.162043
O -4.88518 -1.0063 0.678546
C -4.82604 -2.41698 0.810649
H 4.326648 -1.12994 -1.57252
H 3.777812 1.099682 -0.98724
H 2.071864 -2.18513 -1.8928
H 2.140536 -0.47963 -2.35197
H 1.576376 2.267371 -1.36887

H 4.060524 -2.28143 -0.72169
H -0.35724 -0.1398 -1.96813
H 4.962681 1.048655 -0.92337
H 3.598975 3.435554 -0.14667
H -5.88717 2.983842 0.53124
H -4.37059 2.804901 1.456837
H -4.31454 3.092439 -0.3084

2β10β10 (5.39 %)

C 3.032259 1.625254 0.350364
C 3.801572 0.294135 0.402903
C 2.884207 -0.94209 0.560722
C 1.725556 -0.88272 -0.52064
C 0.989988 0.479051 -0.68845
C 2.016511 1.620846 -0.78159
C 0.814986 -2.08031 -0.41482
C -0.51459 -2.20574 -0.22704
C -1.59878 -1.22921 -0.05686
C -1.42632 0.146656 0.200327
C -0.06536 0.769409 0.417941
C -2.91931 -1.71831 -0.16982
C -4.0492 -0.9203 -0.06033
C -3.84946 0.455534 0.174414
C -2.55758 0.968428 0.299577
C -5.44419 -1.47606 -0.1898
C 2.382793 -1.00973 2.020957
C 3.745685 -2.20397 0.319172
H 2.256138 -0.99406 -1.47736
O 0.356292 0.512498 -1.97346
O 4.57234 0.169283 -0.78536
O 3.960327 2.688552 0.064861
O -4.98783 1.205688 0.267243
C -4.86499 2.601493 0.489208
H 2.541664 1.817515 1.314827
H 4.479557 0.319248 1.278824
H 2.555588 1.503053 -1.72652
H 1.495569 2.582854 -0.82508
H 1.337864 -3.02513 -0.55112

H -0.87827 -3.22938 -0.24531
H -0.20541 1.865736 0.459253
H 0.31534 0.466015 1.37838
H -3.05325 -2.78563 -0.3487
H -2.41501 2.027442 0.471215
H -5.41392 -2.56721 -0.3526
H -6.02941 -1.29915 0.72809
H -5.98949 -1.01918 -1.00573
H 1.607739 -1.77329 2.131898
H 3.205846 -1.25561 2.693312
H 1.953977 -0.05918 2.387723
H 4.653228 -2.14838 0.938769
H 3.211373 -3.10097 0.614189
H 4.037298 -2.29021 -0.72431
H -0.37054 -0.13659 -1.9686
H 5.294775 -0.33602 -0.67876
H 4.547274 2.453727 -0.48837
H -5.89413 2.980071 0.516078
H -4.37354 2.811951 1.43726
H -4.32483 3.085426 -0.33033

H -0.69326 2.808107 -1.89291
H -0.54793 -2.02467 -1.4402
H -0.17481 -0.5478 -2.33327
H -2.91621 2.85213 -0.46788
H -2.53882 -2.07005 -0.20287
H -5.13737 2.805825 0.379111
H -6.06091 1.349958 -0.0485
H -5.41835 1.525112 1.576814
H 1.910979 2.734067 1.63548
H 3.665547 2.472025 1.669326
H 2.87036 2.943497 0.165432
H 3.5742 0.262702 2.615673
H 1.848535 0.600939 2.806531
H 2.356273 -0.92866 2.112131
H 1.833343 -2.17472 0.626833
H 5.664936 0.392004 -0.04727
H 4.357836 -1.71767 0.782277
H -5.78202 -2.71741 1.244246
H -4.01047 -2.72598 1.477363
H -4.6994 -2.90937 -0.16275

H -0.88256 -3.2319 -0.24741
H -0.19823 1.855044 0.485052
H 0.31838 0.443036 1.389827
H -3.05856 -2.78102 -0.35901
H -2.4083 2.026193 0.485942
H -5.41844 -2.5553 -0.36833
H -6.03359 -1.29022 0.715986
H -5.98731 -1.00277 -1.0165
H 1.611867 -1.77897 2.129879
H 3.213784 -1.27106 2.687033
H 1.965239 -0.06745 2.388336
H 4.652413 -2.16056 0.934178
H 3.205297 -3.11401 0.598944
H 4.059444 -2.28399 -0.7246
H -0.36263 -0.14035 -1.96443
H 4.874717 1.074494 -0.98431
H 4.514796 2.814687 0.852138
H -5.88455 2.990514 0.517796
H -4.36819 2.816042 1.444605
H -4.31165 3.091637 -0.32243

2β10β13 (1.93%)

C 3.536821 -0.9995 -0.80997
C 3.716756 0.38007 -0.15941
C 2.552399 0.844944 0.771438
C 1.163765 0.58957 0.07158
C 1.001632 -0.84509 -0.54553
C 2.177798 -1.14858 -1.49401
C 0.78228 1.664785 -0.93271
C -0.49202 1.946847 -1.25939
C -1.63747 1.152382 -0.79236
C -1.5412 -0.25335 -0.73566
C -0.33509 -0.95461 -1.32408
C -2.84214 1.76429 -0.40379
C -3.92632 1.044285 0.085684
C -3.79342 -0.35795 0.177291
C -2.6223 -0.99317 -0.24119
C -5.20673 1.713653 0.516071

2β10β19 (9.57%)

C 3.03024 1.645495 0.358409
C 3.794289 0.316461 0.417659
C 2.878235 -0.9279 0.564291
C 1.724903 -0.87551 -0.52207
C 0.988718 0.48558 -0.69907
C 2.0179 1.625079 -0.78853
C 0.817501 -2.07598 -0.41665
C -0.51166 -2.20256 -0.22749
C -1.59724 -1.22775 -0.05809
C -1.42782 0.150054 0.19196
C -0.06904 0.780141 0.402898
C -2.91661 -1.72166 -0.16478
C -4.04864 -0.92684 -0.05557
C -3.85234 0.450759 0.172206
C -2.56161 0.9683 0.290771
C -5.44231 -1.48743 -0.178

2β10β20 (3.59%)

C -3.52761 1.311417 -0.47511
C -3.55687 0.325782 0.723978
C -2.90619 -1.03813 0.376455
C -1.37383 -0.84257 0.046214
C -1.02281 0.580646 -0.55792
C -2.20549 1.223816 -1.28766
C -0.78309 -2.04485 -0.67308
C 0.52768 -2.29113 -0.85043
C 1.615313 -1.3542 -0.54401
C 1.466462 0.022606 -0.80122
C 0.21529 0.540354 -1.4749
C 2.838347 -1.81225 -0.02128
C 3.885645 -0.95813 0.306135
C 3.701854 0.423418 0.080743
C 2.511041 0.900789 -0.47421
C 5.183496 -1.46384 0.882092

C	2.761383	2.350814	1.059883
C	2.598532	0.159055	2.159167
H	0.405283	0.622283	0.864612
O	0.930479	-1.84541	0.477007
O	4.99019	0.442176	0.472027
O	3.726515	-1.99051	0.236595
O	-4.88518	-1.01408	0.678479
C	-4.82264	-2.42407	0.813591
H	4.347541	-1.1206	-1.54103
H	3.772622	1.094754	-0.99122
H	2.068836	-2.16167	-1.90567
H	2.148557	-0.45394	-2.34341
H	1.569907	2.273611	-1.36926
H	-0.70069	2.805549	-1.89794
H	-0.54161	-2.02427	-1.43158
H	-0.17393	-0.54933	-2.32998
H	-2.9246	2.847617	-0.4719
H	-2.53515	-2.07311	-0.19855
H	-5.14634	2.796932	0.372899
H	-6.06586	1.337839	-0.05247
H	-5.42471	1.51785	1.572899
H	1.92902	2.75075	1.650264
H	3.686057	2.489381	1.62861
H	2.846033	2.954617	0.151412
H	3.57323	0.328999	2.628626
H	1.839264	0.60901	2.808985
H	2.407498	-0.91121	2.13657
H	1.846407	-2.09778	0.690773
H	5.060856	-0.37651	0.991769
H	3.962467	-2.83036	-0.18706
H	-5.77829	-2.72611	1.246794
H	-4.00703	-2.72972	1.481918
H	-4.69373	-2.91835	-0.15862

C	2.37504	-1.00138	2.023591
C	3.742199	-2.18902	0.324547
H	2.258314	-0.9867	-1.47793
O	0.358598	0.505829	-1.9864
O	4.569326	0.293962	-0.79538
O	3.945829	2.725298	0.228097
O	-4.99234	1.197587	0.265195
C	-4.87335	2.594914	0.482256
H	2.522764	1.810151	1.314977
H	4.479352	0.35116	1.278919
H	2.561484	1.50449	-1.73203
H	1.49736	2.587203	-0.83811
H	1.340253	-3.02124	-0.55372
H	-0.87819	-3.2293	-0.24623
H	-0.20547	1.865659	0.459895
H	0.315405	0.465702	1.378509
H	-3.05319	-2.7857	-0.34847
H	-2.41502	2.027276	0.471979
H	-5.41391	-2.56756	-0.35078
H	-6.02957	-1.29839	0.728486
H	-5.98922	-1.02022	-1.00565
H	1.607669	-1.77444	2.131014
H	3.205713	-1.25701	2.692733
H	1.953853	-0.06044	2.387811
H	4.65301	-2.1489	0.937884
H	3.211192	-3.10121	0.612208
H	4.037568	-2.28938	-0.72545
H	-0.37157	-0.13441	-1.96829
H	5.295766	-0.33469	-0.67794
H	4.547315	2.453585	-0.48735
H	-5.89416	2.980427	0.512633
H	-4.37438	2.813951	1.435372
H	-4.32418	3.083966	-0.33274

C	-3.71301	-1.65817	-0.79282
C	-3.03273	-1.97457	1.594299
H	-0.8639	-0.82386	1.019875
O	-0.71192	1.481241	0.542859
O	-2.99536	0.94796	1.882053
O	-3.78923	2.626155	-0.01492
O	4.758837	1.21612	0.427021
C	4.657048	2.615861	0.215484
H	-4.34928	1.052782	-1.15336
H	-4.60326	0.146859	0.996468
H	-1.93114	2.240793	-1.58476
H	-2.37439	0.651702	-2.20811
H	-1.47193	-2.82823	-0.97292
H	0.820191	-3.26439	-1.24503
H	0.385648	1.558918	-1.84087
H	-0.01929	-0.08973	-2.34078
H	2.966046	-2.87956	0.148915
H	2.389417	1.957063	-0.68973
H	5.165616	-2.55291	0.984949
H	6.034559	-1.1903	0.247289
H	5.379101	-1.0273	1.868746
H	-3.52663	-2.73264	-0.88474
H	-4.78731	-1.54338	-0.60568
H	-3.49375	-1.2022	-1.76302
H	-4.08514	-2.21024	1.794938
H	-2.50531	-2.91998	1.419913
H	-2.61912	-1.50371	2.490608
H	0.153139	1.205163	0.895858
H	-2.0669	1.179999	1.655915
H	-3.53028	2.616669	0.926277
H	5.599333	3.040241	0.566543
H	3.827336	3.050815	0.787967
H	4.525316	2.854499	-0.84793

Table S12. ^{1}H NMR spectroscopic data of compounds **1–6**.

	**1** [a]	**2** [b]	**3** [b]	**4** [c]	**5** [b]	**6** [a]
No.	δ_H (*J* in Hz)	δ_H (*J* in Hz)	δ_H (*J* in Hz)	δ_H (*J* in Hz)	δ_H (*J* in Hz)	δ_H (*J* in Hz)
1	2.42 br d (13.2)	2.16 td (14.2, 4.4)	2.52 td (14.0, 4.3)	2.39 m	1.98 m	2.25 dd (14.5, 3.7)
	2.28 td (13.2, 5.4)	1.88 m	1.59 m	2.25 m	1.98 m	1.95 d (14.5, 2.0)
2	2.10 m	1.88 m	2.01 m	1.97 m	2.24 m	4.00 ddd (3.7, 3.0, 2.0)
	2.05 m	1.73 m	1.85 m	1.97 m	1.50 m	
3	3.81 br s	3.40 dd (11.7, 3.4)	3.62 br s	3.62 m	3.88 t (8.0)	3.63 br d (3.0)
5	2.94 br s	0.91 d (2.4)	1.29 d (3.6)	2.37 m	1.56 d (6.2)	2.39 dd (4.6, 2.2)
6	5.90 br d (9.8)	4.21 m	4.15 m	1.75 m	4.44 t (6.2)	5.85 dd (12.0, 4.6)
				1.56 m		
7	6.60 dd (9.8, 2.9)	4.72 d (4.4)	4.71 d (4.3)	2.89 m	4.72 d (6.2)	6.61 dd (12.0, 2.2)
				2.37 m		
11	6.93 s	6.67 s	6.73 s	4.31 br d (8.0)	6.58 s	6.58 s
12				4.05 br s		
14	6.89 s	7.12 s	7.11 s	5.35 s	6.85 s	6.96 s
15	2.18 s	2.22 s	2.21 s	5.91 dd (17.4, 10.4)	2.18 s	2.18 s
16				5.21 d (17.4)		
				5.11 d (10.4)		
17				1.36 s		
18	1.19 s	1.14 s	1.11 s	1.29 s	1.03 s	1.03 s
19	4.37 d (12.2)	1.14 s	1.17 s	1.16 s	1.10 s	1.25 s
	4.04 dd (12.2, 1.7)					
20		4.07 d (8.5)	4.11 d (8.5)	1.08 s	2.98 d (16.3)	2.95 d (14.0)
		2.72 d (8.5)	2.75 d (8.5)		2.55 d (16.3)	2.68 d (14.0)
OMe	3.88 s	3.86 s	3.86 s		3.80 s	3.84 s
3-O*H*				5.75 br d (4.6)		
9-O*H*				4.79 br s		
11-O*H*				5.15 overlapped		
12-O*H*				6.87 br s		

[a] Spectra recorded at 400 MHz in $CDCl_3$; [b] Spectra recorded at 500 MHz in $CDCl_3$; [c] Spectra recorded at 400 MHz in pyridine-d_5.
